# Supplementary material for: Country adoption of WHO 2019 guidance on HIV testing strategies and algorithms: a policy review across the WHO African region
Source: BMJ Open. 2023 Dec 28;13(12):e071198. doi: 10.1136/bmjopen-2022-071198 (PMC10759095; doi:10.1136/bmjopen-2022-071198)
Supplement: Supplementary data [file bmjopen-2022-071198supp001.pdf]

## Supplementary Appendix

Country adoption of WHO 2019 guidance on HIV testing strategies and algorithms: a policy review across the WHO African region

Emmanuel Fajardo,[1] Céline Lastrucci,[1] Nayé Bah,[2] Casimir Manzengo Mingiedi,[3] Ndoungou Salla Ba,[4] Fausta Shakiwa Mosha,[5] Frank John Lule,[6] Margaret Alia Sampson Paul,[6] Lago Hughes,[6] Magdalena Barr-DiChiara,[1] Muhammad Shahid Jamil,[1] Anita Sands,[7] Rachel Baggeley,[1] Cheryl Johnson[1]

**Supplementary Figure 1.** Countries in the WHO African Region and grouping by subregion

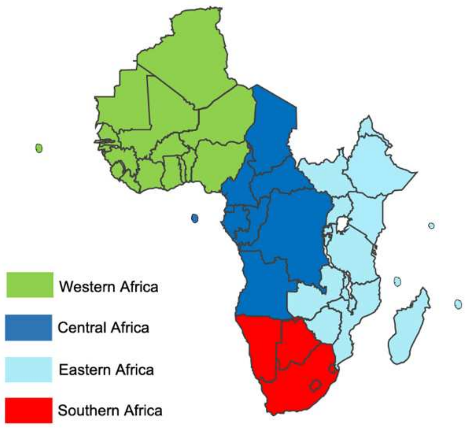


| WHO African Region (n=47) | | | |
|---|---|---|---|
| Western Africa (n=17) | Central Africa (n=9) | Eastern Africa (n=16) | Southern Africa (n=5) |
| Algeria | Angola | Burundi | Botswana |
| Cabo Verde | Cameroon | Comoros | eSwatini |
| Burkina Faso | CAR | Eritrea | Lesotho |
| Benin | Chad | Ethiopia | Namibia |
| Ghana | DRC | Kenya | South Africa |
| Guinea | Equotorial Guinea | Madagascar | |
| Guinea-Bissau | Gabon | Malawi | |
| Ivory Coast | Republic of the Congo | Mauritius | |
| Liberia | Sao Tome and Principe | Mozambique | |
| Mauritania | | Rwanda | |
| Mali | | Seychelles | |
| Niger | | South Sudan | |
| Nigeria | | Tanzania | |
| Senegal | | Uganda | |
| Sierra Leone | | Zambia | |
| The Gambia | | Zimbabwe | |
| Togo | | | |



Fajardo E, *et al. BMJ Open* 2023; 13:e071198. doi: 10.1136/bmjopen-2022-071198

**Supplementary Table 1.** Categories for data extraction

| | |
|---|---|
| **1. Policy Information** | |
| ▪ Country<br>▪ WHO region<br>▪ HIV testing policy identified? | ▪ Year of policy publication<br>▪ Type of policy document<br>▪ National HIV prevalence |
| **2. HIV testing algorithm (after 18 months of age)** | |
| ▪ HIV testing algorithm provided?<br>▪ Image or text format?<br>▪ Type of scenario in which algorithm is used<br>▪ Number of assays used in algorithm<br>▪ Serial or parallel strategy<br>▪ Use of tiebreaker?<br>▪ Use of Western Blot or LIA?<br>▪ Mention of test order relating to sens/spec?<br>▪ High or low prevalence strategy used?<br>▪ Type of tests mentioned?<br>▪ Brand of tests mentioned?<br>▪ Are assays pre-qualified by WHO?<br>▪ A1 test name<br>▪ A1 type of assay | ▪ A1 sens-/spec<br>▪ A2 test name<br>▪ A2 type of assay<br>▪ A2 sens-/spec<br>▪ A3 test name<br>▪ A3 type of assay<br>▪ A3 sens-/spec<br>▪ Source of assay sens/spec<br>▪ Mention of in-country assay validation<br>▪ Alignment with WHO policy?<br>▪ Primary reason(s) algorithm fails to meet WHO recommendations<br>▪ Other notes about testing strategy |
| **3. HIV testing algorithm among pregnant women using dual HIV/Syphilis RDTs** | |
| ▪ HIV testing algorithm provided?<br>▪ Image or text format?<br>▪ Type of scenario in which algorithm is used<br>▪ Type of scenario in which algorithm should not be used (women on ART, already diagnosed/treated for syphilis, retesting for HIV)<br>▪ What is there syphilis testing strategy for women with HIV?<br>▪ Is dual test used as A1 or A0?<br>▪ Is the dual test algorithm aligned with the national HIV testing algorithm?<br>▪ Is the dual test algorithm aligned with the national syphilis testing algorithm?<br>▪ Brand of dual test mentioned? | ▪ Is dual test prequalified by WHO?<br>▪ Dual test name<br>▪ Mention of penicillin treatment for reactive TP?<br>▪ Mention of further testing for reactive TP?<br>▪ Mention of in-country assay validation<br>▪ Alignment with WHO policy?<br>▪ Primary reason(s) algorithm fails to meet WHO recommendations<br>▪ Other notes about testing strategy |
| **4. Retesting prior to ART initiation** | |
| ▪ Retest required before starting ART?<br>▪ Retest strategy same as national algorithm?<br>▪ Notes about retesting strategy | |



Fajardo E, *et al. BMJ Open* 2023; 13:e071198. doi: 10.1136/bmjopen-2022-071198

**Supplementary Table 2.** List of national policies on HIV testing services in the WHO African Region collected in 2018 and 2021 by subregion

| Subregion | Count | Country | Policy reviewed in 2018? | New policy or algorithm identified? | Publication year | Type of document | Language | Algorithm provided? | National HIV prevalence |
|---|---|---|---|---|---|---|---|---|---|
| Central Africa | 1 | Angola | Yes, 2015 | Yes | 2020 | HTS | Portuguese | Yes | Low |
| | 2 | Cameroon | Yes, 2015 | Yes | 2019 | ART | French | Yes | Low |
| | 3 | CAR | Yes, 2010 | Yes | 2018 | ART | French | Yes | Low |
| | 4 | Chad | Yes, 2011 | Yes | 2017 | HTS | French | Yes | Low |
| | 5 | DRC | Yes, 2017 | Yes | 2020 | ART | French | Yes | Low |
| | 6 | Equatorial Guinea | No | Yes | 2018 | ART | Spanish | Yes | Low |
| | 7 | Gabon | No | Yes | 2017 | HTS | French | Yes | Low |
| | 8 | Republic of the Congo | No | **No** | – | – | – | – | – |
| | 9 | São Tomé e Principe | No | Yes | 2018 | Algorithm | Portuguese | Yes | Low |
| Eastern Africa | 10 | Burundi | Yes, 2016 | Yes | 2020 | HTS | French | Yes | Low |
| | 11 | Comoros | Yes, 2007 | Yes | 2016 | HTS | French | Yes | Low |
| | 12 | Eritrea | No | Yes | 2019 | HTS | English | Yes | Low |
| | 13 | Ethiopia | Yes, 2017 | Yes | 2018 | ART | English | Yes | Low |
| | 14 | Kenya | Yes, 2017 | Yes | 2021 | HTS | English | Yes | Low |
| | 15 | Madagascar | Yes, 2011 | Yes | 2018 | HTS | French | Yes | Low |
| | 16 | Malawi* | Yes, 2016 | Yes* | 2016 | HTS | English | Yes | High |
| | 17 | Mauritius | No | Yes | 2020 | HTS | English | Yes | Low |
| | 18 | Mozambique | Yes, 2016 | Yes | 2020 | PMTCT | Portuguese | Yes | High |
| | 19 | Rwanda | Yes, 2016 | Yes | 2018 | HTS | English | Yes | Low |
| | 20 | Seychelles | No | Yes | 2019 | HTS | English | Yes | Low |
| | 21 | South Sudan | Yes, 2017 | Yes | 2020 | ART | English | Yes | Low |
| | 22 | Tanzania | Yes, 2017 | Yes | 2021 | HTS | English | Yes | High |
| | 23 | Uganda | Yes, 2016 | Yes | 2020 | ART | English | Yes | High |
| | 24 | Zambia | Yes, 2018 | Yes | 2020 | HTS | English | Yes | High |
| | 25 | Zimbabwe* | Yes, 2016 | Yes* | 2016 | Other | English | Yes | High |
| Southern Africa | 26 | Botswana** | Yes, 2016 | **No** | 2016 | ART | English | Yes | High |
| | 27 | eSwatini** | Yes, 2018 | **No** | 2018 | ART | English | Yes | High |
| | 28 | Lesotho | Yes, 2016 | Yes | 2021 | PMTCT | English | Yes | High |

| | | | | | | | | | |
|---|---|---|---|---|---|---|---|---|---|
| | 29 | Namibia | Yes, 2016 | Yes | 2018 | HTS | English | Yes | High |
| | 30 | South Africa | Yes, 2016 | Yes | 2020 | ART | English | Yes | High |
| Western Africa | 31 | Algeria** | Yes, 2013 | **No** | 2013 | HTS | French | Yes | Low |
| | 32 | Benin | No | Yes | 2017 | HTS | French | Yes | Low |
| | 33 | Burkina Faso | Yes, 2008 | Yes | 2021 | ART | French | Yes | Low |
| | 34 | Cabo Verde | No | **No** | – | – | – | – | – |
| | 35 | Ghana | Yes, 2014 | Yes | 2019 | ART | English | Yes | Low |
| | 36 | Guinea | No | Yes | 2019 | HTS | French | Yes | Low |
| | 37 | Guinea-Bissau | No | Yes | 2021 | ART | Portuguese | Yes | Low |
| | 38 | Ivory Coast** | Yes, 2016 | **No** | 2016 | HTS | French | Yes | Low |
| | 39 | Liberia | Yes, 2015 | Yes | 2020 | HTS | English | Yes | Low |
| | 40 | Mauritania | No | Yes | 2020 | HTS | French | Yes | Low |
| | 41 | Mali | No | Yes | 2017 | HTS | French | Yes | Low |
| | 42 | Niger | No | Yes | 2020 | HTS | French | Yes | Low |
| | 43 | Nigeria | Yes, 2016 | Yes | 2020 | ART | English | Yes | Low |
| | 44 | Senegal | Yes, 2017 | Yes | 2018 | HTS | French | Yes | Low |
| | 45 | Sierra Leone | Yes, 2017 | Yes | 2020 | ART | English | Yes | Low |
| | 46 | The Gambia | Yes, 2014 | Yes | 2019 | ART | English | Yes | Low |
| | 47 | Togo | No | Yes | 2019 | HTS | French | Yes | Low |

*These countries provided an updated HIV testing algorithm despite having an old HIV testing guideline

** These countries did not provide an update HIV testing guideline but were included in the analysis

**Supplementary Table 3.** Progress on policy adoption in the WHO African Region from 2018 to 2021

| Subregion | Count | Country | Policy reviewed in 2018? | Adherence in 2018 | Policy reviewed in 2021 | Adherence in 2021 |
|---|---|---|---|---|---|---|
| Central Africa | 1 | Angola | Yes, 2015 | Not adopted | 2020 | Mostly adopted |
| | 2 | Cameroon | Yes, 2015 | Not adopted | 2019 | Mostly adopted |
| | 3 | CAR | Yes, 2010 | Not adopted | 2018 | Not adopted |
| | 4 | Chad | Yes, 2011 | Not adopted | 2017 | Mostly adopted |
| | 5 | DRC | Yes, 2017 | Adopted | 2020 | Adopted |
| | 6 | Equatorial Guinea | No | – | 2018 | Not adopted |
| | 7 | Gabon | No | – | 2017 | Mostly adopted |
| | 8 | Republic of the Congo | No | – | – | – |
| | 9 | São Tomé e Principe | No | – | 2018 | Not adopted |
| Eastern Africa | 10 | Burundi | Yes, 2016 | Not adopted | 2020 | Mostly adopted |
| | 11 | Comoros | Yes, 2007 | No information | 2016 | Not adopted |
| | 12 | Eritrea | No | – | 2019 | Not adopted |
| | 13 | Ethiopia | Yes, 2017 | No information | 2018 | Mostly adopted |
| | 14 | Kenya | Yes, 2017 | Mostly adopted | 2021 | Adopted |
| | 15 | Madagascar | Yes, 2011 | No information | 2018 | Mostly adopted |
| | 16 | Malawi | Yes, 2016 | Not adopted | 2016 | Mostly adopted |
| | 17 | Mauritius | No | – | 2020 | Not adopted |
| | 18 | Mozambique | Yes, 2016 | Not adopted | 2020 | Mostly adopted |
| | 19 | Rwanda | Yes, 2016 | Not adopted | 2018 | Mostly adopted |
| | 20 | Seychelles | No | – | 2019 | Not adopted |
| | 21 | South Sudan | Yes, 2017 | Not adopted | 2020 | Adopted |
| | 22 | Tanzania | Yes, 2017 | Not adopted | 2021 | Mostly adopted |
| | 23 | Uganda | Yes, 2016 | Mostly adopted | 2020 | Mostly adopted |
| | 24 | Zambia | Yes, 2018 | Not adopted | 2020 | Not adopted |
| | 25 | Zimbabwe | Yes, 2016 | Adopted | 2016 | Adopted |
| Southern Africa | 26 | Botswana | Yes, 2016 | Mostly adopted | 2016 | Not adopted |
| | 27 | eSwatini | Yes, 2018 | Not adopted | 2018 | Not adopted |
| | 28 | Lesotho | Yes, 2016 | Adopted | 2021 | Mostly adopted |
| | 29 | Namibia | Yes, 2016 | No information | 2018 | Not adopted |
| | 30 | South Africa | Yes, 2016 | Mostly adopted | 2020 | Not adopted |
| Western Africa | 31 | Algeria | Yes, 2013 | Adopted | 2013 | Not adopted |
| | 32 | Benin | No | – | 2017 | Not adopted |
| | 33 | Burkina Faso | Yes, 2008 | Not adopted | 2021 | Mostly adopted |
| | 34 | Cabo Verde | No | – | – | – |
| | 35 | Ghana | Yes, 2014 | Not adopted | 2019 | Mostly adopted |
| | 36 | Guinea | No | – | 2019 | Mostly adopted |
| | 37 | Guinea-Bissau | No | – | 2021 | Not adopted |
| | 38 | Ivory Coast | Yes, 2016 | Not adopted | 2016 | Not adopted |
| | 39 | Liberia | Yes, 2015 | Not adopted | 2020 | Mostly adopted |
| | 40 | Mauritania | No | – | 2020 | Not adopted |
| | 41 | Mali | No | – | 2017 | Not adopted |
| | 42 | Niger | No | – | 2020 | Not adopted |
| | 43 | Nigeria | Yes, 2016 | Not adopted | 2020 | Mostly adopted |
| | 44 | Senegal | Yes, 2017 | Mostly adopted | 2018 | Not adopted |
| | 45 | Sierra Leone | Yes, 2017 | Not adopted | 2020 | Not adopted |
| | 46 | The Gambia | Yes, 2014 | Not adopted | 2019 | Not adopted |
| | 47 | Togo | No | – | 2019 | Not adherent |

A total of 32 countries had policies reviewed in 2018 and 2021. Those highlighted in red are policies reviewed in 2018 that were updated (n=28). Number of policies deemed adopted in 2018 (n=7) and number deemed adopted in 2021 (n=20)



Fajardo E, *et al. BMJ Open* 2023; 13:e071198. doi: 10.1136/bmjopen-2022-071198

**Supplementary Figure 2.** Comparing policy adoption across countries, 2018 vs 2021

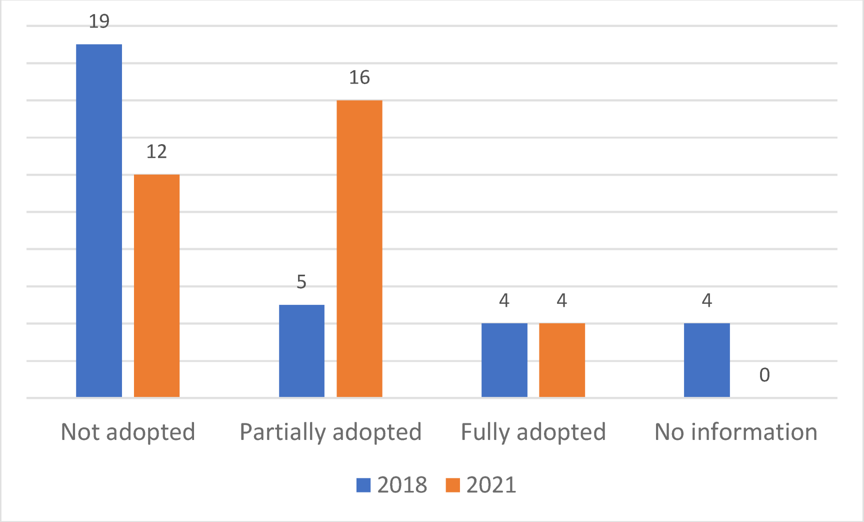




Fajardo E, *et al. BMJ Open* 2023; 13:e071198. doi: 10.1136/bmjopen-2022-071198

**Supplementary Table 4.** Policy adoption of 2019 WHO-recommended HIV testing strategies in the WHO African region, 2014-2021

| **AFRO subregions (countries)** | **Policies reviewed** | | | **Serial testing strategy** | | | **Discontinuation of tiebreaker* testing strategy** | | | **Retesting prior to ART initiation** | | **Universal 3-test strategy** | **Use of dual HIV/syphilis test**** | **No use of Western Blot** |
|---|---|---|---|---|---|---|---|---|---|---|---|---|---|---|
| Year | 2014 | 2018 | 2021 | 2014 | 2018 | 2021 | 2014 | 2018 | 2021 | 2018 | 2021 | 2021 | 2021 | 2021 |
| All (n=47) | 25 (53%) | 32 (68%) | 45 (96%) | 21 (84%) | 26 (81%) | 29 (64%) | 15 (60%) | 26 (81%) | 37 (82%) | 16 (50%) | 23 (51%) | 21 (47%) | 21 (47%) | 35 (78%) |
| Western (n=17) | 6 (35%) | 9 (53%) | 16 (94%) | 5 (83%) | 7 (78%) | 9 (56%) | 4 (67%) | 5 (56%) | 9 (56%) | 1 (11%) | 5 (31%) | 8 (50%) | 7 (44%) | 10 (63%) |
| Central (n=9) | 4 (44%) | 5 (56%) | 8 (89%) | 4 (100%) | 5 (100%) | 7 (88%) | 3 (75%) | 4 (80%) | 7 (88%) | 1 (20%) | 5 (63%) | 3 (38%) | 2 (25%) | 6 (75%) |
| Eastern (n=16) | 11 (69%) | 13 (81%) | 16 (100%) | 10 (91%) | 10 (77%) | 12 (75%) | 5 (45%) | 12 (92%) | 16 (100%) | 9 (69%) | 9 (56%) | 9 (56%) | 9 (56%) | 14 (88%) |
| Southern (n=5) | 4 (80%) | 5 (100%) | 5 (100%) | 2 (40%) | 4 (80%) | 1 (20%) | 3 (75%) | 5 (100%) | 5 (100%) | 5 (100%) | 4 (80%) | 1 (20%) | 3 (60%) | 5 (100%) |

*Use of a third assay to rule-in HIV infection

**Dual HIV/syphilis RDT as first test in ANC

**Supplementary Table 5.** Short name of products used as part of the 2-assay or 3-assay HIV testing algorithm in 28 HIV national testing policy documents reviewed in 2021

| Strategy | Count | Country | Assay 1 | Assay 2 | Assay 3 |
|---|---|---|---|---|---|
| **2-assay** | 1 | Angola | Determine | Uni-Gold | – |
| | 2 | Cameroon | Determine or Uni-Gold | OraQuick or Shanghai | – |
| | 3 | CAR | Determine | Uni-Gold | – |
| | 4 | Cote D'Ivoire | Determine | SD Bioline or GenieFast | StatPak or EIA (as tiebreaker) |
| | 5 | Equatorial Guinea | Determine | Hexagon | Uni-Gold (for discordants) |
| | 6 | eSwatini | Determine | Uni-Gold | – |
| | 7 | Gambia | Determine | SD Bioline or First Response | – |
| | 8 | Guinea Bissau | Determine | First Response | PCR (as tiebreaker) |
| | 9 | Mali | Alere Combo | SD Bioline | First Response (as tiebreaker) |
| | 10 | Mozambique | Determine | Uni-Gold | – |
| | 11 | Rwanda | Alere Combo | StatPak | – |
| | 12 | São Tomé e Principe | Determine | SD Bioline | PCR (as tiebreaker) |
| | 13 | Sierra Leone | Determine | SD Bioline | Uni-Gold (as tiebreaker) |
| | 14 | Uganda | Determine | StatPak | SD Bioline (for discordants) |
| | 15 | Nigeria | Determine<br>Uni-Gold<br>Stat-Pak<br>Double Check Gold<br>Sure Check<br>HIV Quick Check | Determine<br>Uni-Gold<br>Stat-Pak<br>Double Check Gold<br>Sure Check<br>HIV Quick Check | Determine<br>Uni-Gold<br>Stat-Pak<br>Double Check Gold<br>Sure Check<br>HIV Quick Check<br>(as tiebreaker) |
| | 16 | Togo | Murex HIV Ag/Ab<br>Apdia HIV Ag/Ab<br>Determine<br>Alere Combo<br>Vikia HIV 1/2<br>Wanta Rapid Test<br>ABON HIV 1/2/0<br>Standard Q<br>Hexagon<br>Genie Fast<br>SD Bioline<br>First Response<br>SD Bioline HIV/Syphilis<br>OraQuick HIV Self-Test | ABON HIV 1/2/0<br>Standard Q<br>SD Bioline<br>First Response<br>HIV Tri-Dot | INNOLIA or Genius (as tiebreaker) |
| **3-assay** | 17 | Burundi | Alere Combo or Determine | SD Bioline or Uni-Gold | Wondfo One Step |
| | 18 | Gabon | Alere Combo | Determine | SD Bioline |
| | 19 | Ghana | First Response | OraQuick | SD Bioline |
| | 20 | Guinea | Determine | SD Bioline | Multisure |
| | 21 | Kenya | INSTI | Uni-Gold | SD Bioline |
| | 22 | Liberia | Determine | SD Bioline | Uni-Gold |
| | 23 | Madagascar | Determine | Uni-Gold | SD Bioline |
| | 24 | Mauritius | GenScreen Ultra Ag/Ab | Alere Combo | Western Blot |
| | 25 | Malawi | INSTI | Uni-Gold | SD Bioline |



Fajardo E, *et al. BMJ Open* 2023; 13:e071198. doi: 10.1136/bmjopen-2022-071198

| | | | | | |
|---|---|---|---|---|---|
| | 26 | Niger | Alere Combo | Wondfo One Step | SD Bioline |
| | 27 | Senegal | Determine | SD Bioline | Multisure |
| | 28 | Zimbabwe | Determine | Chembio | INSTI |
| | 29 | Burkina Faso | 3rd generation:<br>Determine<br>Double check Gold Ultra<br>Onsite HIV1+2 Plus Combo<br>VIKIA HIV 1/2<br>4th generation:<br>Alere Ag/Ab Combo<br>OnSite HIV Ab/Ag (CTK) | HIV TriDot<br>ImmunoFlow<br>OnSite HIV 1/2 Ab Plus (CTK)<br>SD Bioline | Any of those not selected as A1 or A2 |



Fajardo E, *et al. BMJ Open* 2023; 13:e071198. doi: 10.1136/bmjopen-2022-071198
