# Supplementary material for: Country adoption of WHO 2019 guidance on HIV testing strategies and algorithms: a policy review across the WHO African region
Source: BMJ Open. 2023 Dec 28;13(12):e071198. doi: 10.1136/bmjopen-2022-071198 (PMC10759095; doi:10.1136/bmjopen-2022-071198)
Supplement: Author's manuscript [file bmjopen-2022-071198.draft_revisions.pdf]

BMJ Open is committed to open peer review. As part of this commitment we make the peer review history of every article we publish publicly available.

When an article is published we post the peer reviewers' comments and the authors' responses online. We also post the versions of the paper that were used during peer review. These are the versions that the peer review comments apply to.

The versions of the paper that follow are the versions that were submitted during the peer review process. They are not the versions of record or the final published versions. They should not be cited or distributed as the published version of this manuscript.

BMJ Open is an open access journal and the full, final, typeset and author-corrected version of record of the manuscript is available on our site with no access controls, subscription charges or pay-per-view fees (http://bmjopen.bmj.com).

If you have any questions on BMJ Open's open peer review process please email info.bmjopen@bmj.com

BMJ Open

Country adoption of WHO 2019 guidance on HIV testing strategies and algorithms: a policy review across the WHO African region

Journal:	*BMJ Open*
Manuscript ID	bmjopen-2022-071198
Article Type:	Original research
Date Submitted by the Author:	22-Dec-2022
Complete List of Authors:	Fajardo, Emmanuel; World Health Organization, Global HIV, Hepatitis, and STI Programmes<br>Lastrucci, Céline; WHO, Global HIV, Hepatitis, and STI Programmes<br>Bah, Nayé; World Health Organization Regional Office for Africa<br>Mingiedi, Casimir Manzengo; World Health Organization Regional Office for Africa, Inter-country support team for Central Africa<br>Ba, Ndoungou Salla; World Health Organization Regional Office for Africa, Inter-country support team for Western and Central Africa<br>Mosha, Fausta; World Health Organization Regional Office for Africa, Inter-country support team for Eastern and Southern Africa<br>Lule, Frank John; World Health Organization Regional Office for Africa<br>Paul, Margaret Alia Sampson; World Health Organization Regional Office for Africa<br>Hughes, Lago; World Health Organization Regional Office for Africa<br>Barr-DiChiara, Magdalena; World Health Organization, Department of Global Programmes of HIV, Hepatitis and HIV<br>Jamil, Muhammad; World Health Organization, Global HIV, Hepatitis, and STI Programmes<br>Sands, Anita ; World Health Organization, Regulation and Prequalification<br>Baggaley, R; World Health Organization<br>Johnson, Cheryl; WHO
Keywords:	Health policy < HEALTH SERVICES ADMINISTRATION & MANAGEMENT, HIV & AIDS < INFECTIOUS DISEASES, Diagnostic microbiology < INFECTIOUS DISEASES

SCHOLARONE™
Manuscripts

I, the Submitting Author has the right to grant and does grant on behalf of all authors of the Work (as defined in the below author licence), an exclusive licence and/or a non-exclusive licence for contributions from authors who are: i) UK Crown employees; ii) where BMJ has agreed a CC-BY licence shall apply, and/or iii) in accordance with the terms applicable for US Federal Government officers or employees acting as part of their official duties; on a worldwide, perpetual, irrevocable, royalty-free basis to BMJ Publishing Group Ltd ("BMJ") its licensees and where the relevant Journal is co-owned by BMJ to the co-owners of the Journal, to publish the Work in this journal and any other BMJ products and to exploit all rights, as set out in our licence.

The Submitting Author accepts and understands that any supply made under these terms is made by BMJ to the Submitting Author unless you are acting as an employee on behalf of your employer or a postgraduate student of an affiliated institution which is paying any applicable article publishing charge ("APC") for Open Access articles. Where the Submitting Author wishes to make the Work available on an Open Access basis (and intends to pay the relevant APC), the terms of reuse of such Open Access shall be governed by a Creative Commons licence – details of these licences and which Creative Commons licence will apply to this Work are set out in our licence referred to above.

Other than as permitted in any relevant BMJ Author's Self Archiving Policies, I confirm this Work has not been accepted for publication elsewhere, is not being considered for publication elsewhere and does not duplicate material already published. I confirm all authors consent to publication of this Work and authorise the granting of this licence.

For peer review only

Title: Country adoption of WHO 2019 guidance on HIV testing strategies and algorithms: a policy review across the WHO African region

Authors: Emmanuel Fajardo,[1] Céline Lastrucci,[1] Nayé Bah,[2] Casimir Manzengo Mingiedi,[3] Ndoungou Salla Ba,[4] Fausta Shakiwa Mosha,[5] Frank John Lule,[6] Margaret Alia Sampson Paul,[6] Lago Hughes,[6] Magdalena Barr-DiChiara,[1] Muhammad Shahid Jamil,[1] Anita Sands,[7] Rachel Baggaley,[1] Cheryl Johnson[1]

Authors affiliations:

[1]Global HIV, Hepatitis and STI Programmes, WHO, Switzerland

[2]Regional Office for Africa, WHO Country Office, Mali

[3]Regional Office for Africa, Inter-country support team for Central Africa, WHO, Gabon

[4]Regional Office for Africa, Inter-country support team for Western and Central Africa, WHO, Burkina Faso

[5]Regional Office for Africa, Inter-country support team for Eastern and Southern Africa, WHO Zimbabwe

[6]Regional Office for Africa, WHO Congo Brazzaville

[7]Incidents and Substandard/Falsified Medical Products Team, Regulation and Prequalification Department, WHO, Switzerland

Corresponding author: Cheryl Johnson, Global HIV, Hepatitis and STI Programmes, WHO, Avenue Appia 20, 1211 Geneva, Switzerland. johnsonc@who.int

For peer review only

ABSTRACT

Introduction: In 2019, the World Health Organization (WHO) released updated guidelines on HIV testing services (HTS) recommending: use of serial testing, a standard 3-assay testing strategy requiring three consecutive reactive serology test results to make an HIV-positive diagnosis; discontinuation of the use of a tie-breaker to rule-in HIV infection, discontinuation of western blotting (WB) for HIV diagnosis, retesting prior to ART initiation, and dual HIV/syphilis rapid diagnostic tests (RDTs) as the first test in antenatal care (ANC). Here we update previous policy reviews conducted in 2014 and 2018 to assess policy adoption of updated recommendations in the WHO African region.

Methods: Between May and December 2021, we undertook a comprehensive desk review of national HTS policies. Adoption of WHO recommendations was assessed based on the number of WHO 2019 HTS recommendations adopted and then compared to 2014 and 2018 baseline information.

Results: National policies were reviewed for 96% (n=45/47) of countries in the WHO African region, 40% (n=18) were published before 2019, and 62% (n=28) adopted WHO guidance. Between 2018 and 2021, adoption in the region increased from 28% (n=9/32) to 62% (n=28/45). Using a 2-assay testing strategy was the most common form of non-adoption: 31% (n=14) and 22% (n=10) in low (<5%) and high (≥5%) prevalence countries. Ten policies (22%) recommended the use of WB in their HIV testing algorithm, and 51% (n=23) recommended retesting before ART initiation. Dual HIV/syphilis RDTs were recommended in 47% (n=21/45) of policies.

For peer review only - http://bmjopen.bmj.com/site/about/guidelines.xhtml

Conclusions: Adoption of WHO-recommended HIV testing strategies has improved in the African region. While WB was only used in a few countries, concerted efforts are needed to phase out this technology in favour of RDTs. Countries should plan to accelerate their transition to WHO recommendations by streamlining efforts to adopt and implement a 3-asssay testing strategy and dual HIV/syphilis RDTs.

Word count/limit: 299

Keywords: HIV, diagnosis, rapid test, dual HIV/syphilis, policy, guideline, Africa, algorithm

For peer review only

STRENGTHS AND LIMITATIONS OF THIS STUDY

⇒ Compared to the 2018 global review where six WHO Regions were included, by focusing only on the WHO African region, we were able to include thirteen additional national policy documents in this review (n=45 in 2021 vs n=32 in 2018).

⇒ We employed robust searching methods and a standardized data extraction tool.

⇒ We didn't find out how HTS policies were being implemented in each country. Countries may be executing HIV testing plans that diverge from those specified in official policy papers, resulting in misclassification of adoption status. It's likely that decentralization in some countries has resulted in changes in testing procedures and strategies at the subnational level that this evaluation overlooked even though we did not include subnational policy documents.

⇒ Notably, during our review we also identified countries in the process of adopting WHO recommendations – particularly the use of 3-test strategy and dual HIV/syphilis RDTs for ANC. Thus, our estimates of adoption of WHO testing guidelines may be conservative.

INTRODUCTION

HIV Testing Services (HTS) is the critical gateway to accessing HIV-related care and treatment for those diagnosed as HIV-positive and as a means to accessing prevention services for those who test HIV-negative.[1, 2] Despite considerable progress, reaching the first UNAIDS 95 HIV target by 2025 will require increased testing uptake, particularly among populations with testing gaps such as key population, youth and men.[3] Further, the global emergency caused by the COVID-19 pandemic in 2020 has disrupted many health services including HIV testing services, and supply chains of key commodities such as rapid diagnostic tests (RDTs) have

For peer review only

been stretched.[4] In 2021, it was estimated that 85% of people living with HIV globally were aware of their serostatus.[5]

In 2019, the World Health Organization (WHO) published consolidated guidelines on HIV testing services (HTS),[6] bringing together existing recommendations specific to testing strategies and algorithms e.g., use of serial testing rather than parallel testing (WHO, 2012),[7] discontinuation of a tiebreaker strategy to rule-in HIV infection (WHO, 2015)[1] and, retesting prior to antiretroviral treatment (ART) initiation, recommended since 2014;[1] and released three new recommendations related to testing strategies namely: (moving toward a universal 3-assay testing strategy using three consecutive HIV-reactive tests to provide and HIV-positive diagnosis, (2) discontinuation of western blot (WB) and line immunoassays (LIA) and, (3) use of dual HIV/Syphilis RDTs as first assay in HTS in antenatal care (ANC) settings. In light of these testing modalities, ensuring that HIV testing is accessible and adheres to the "5 C's" including voluntary consent, confidentiality, counselling, correct results, and linkage to care, is of upmost importance.

National guidelines outlining HIV testing strategies are critical to ensuring a correct and timely HIV diagnosis. Many countries still use the 2015 WHO recommendation of using the national HIV prevalence to determine whether a two-assay (≥5%) or three-assay testing strategy (<5%) should be used.[1] However, as ART coverage expands and HIV positivity declines, WHO now recommends countries to use the national HTS positivity and the treatment-adjusted prevalence, which accounts for both the national HIV prevalence and ART coverage, as an indicator to determine when to transition to a three-assay testing strategy.[8] WHO also recommends that countries planning to update their HIV testing algorithms undertake a

For peer review only

verification study to select appropriate HIV serology products and ensure they don't cross-react in order to minimize the risk of misdiagnosis.[9]

A global review of national HIV testing policies was conducted by WHO in 2018 to assess adoption of its testing recommendations and policies.[10] Of 91 policies reviewed only 24 (25%) adhered to WHO guidance. This policy review seeks to update results of the 2018 review and ascertain adoption of six 2019 WHO recommendations particularly in the WHO African region, which hosts several countries with a high HIV burden that have traditionally used a 2-assay testing strategy. The same policy review across the remaining five WHO regions is underway.

METHODS

Search strategy

We carried out a comprehensive desk review of national HIV testing policies in 47 Member States in the WHO African region (online supplementary figure 1) using a policy repository maintained by WHO, scanning government websites, and by contacting government officials or other relevant informants through December 2021. The most current policy documents containing information on the HTS policy were included, but when this was unavailable, we included the most recent HIV testing algorithm, but if that was also unavailable, we opted to include previous national policies. Documents of all languages were included. Other supporting documents related to algorithm validation and PMTCT guidelines were identified, when possible, either through references provided in the national HTS policy or through contact with key informants.

For peer review only

Data extraction

Data were extracted from each policy document by one reviewer (EF) into standardised coding forms on policy information, HIV testing strategy/algorithm (>18 months of age), dual HIV/syphilis testing strategy/algorithm, and retesting prior to ART initiation (online supplementary table 1). A second reviewer (CL) carried out crosschecking of the data. Differences between coders were resolved through a third reviewer (CJ). To prevent misclassification, items were marked as 'unclear' during data extraction when lack of information prevented complete understanding.

Analysis

We assessed national adoption of WHO HTS guidance using six specific recommendations related to testing strategies and algorithms set forth in the WHO 2019 HIV testing guidelines, namely: (1) use of serial testing, (2) use of a 3-assay testing strategy, (3) discontinuation of a tie-breaker to rule in HIV infection, (4) discontinuation of WB/LIA, (5) retesting prior to ART initiation, and (6) use of dual HIV/syphilis RDT in ANC. Based on the number of recommendations adopted, national policies were categorized as: adopted (6); nearly adopted (5 to 4); somewhat not adopted: (3); and not adopted: (2 or less).

To determine the proportion of countries adopting WHO guidance, country policies categorised as 'fully adopted and 'nearly adopted' were considered adopted and those categorised as 'somewhat not adopted' or 'not adopted' were considered non-adopted. Descriptive analyses disaggregated by subregions (Western, Central, Eastern and Southern Africa; online supplementary figure 1) were also conducted to determine rates of adherence by subregion. All analyses were conducted in Microsoft Excel.

For peer review only

Patient and public involvement

Patients and/or the public were not involved in the design, conduct, reporting, or dissemination plans of this study.

RESULTS

We identified updated national policies or HIV testing algorithms for 41 countries out of 47 (87%) in 2021. Previous policies for 6 countries (Algeria, Botswana, eSwatini, Cote d'Ivoire, Malawi, and Zimbabwe) identified in the 2018 review were included in this current analysis, with Malawi and Zimbabwe providing updated HIV testing algorithms. São Tomé e Principe was included based on the HIV testing algorithm provided but without a national policy document. In total, we included 96% of countries from the WHO African region (n=45/47). Two countries, Republic of the Congo and Cabo Verde, were not included because we were unable to obtain their national policies (figure 1).

Of the 45 policies providing information on HIV testing strategies, 16 were from Western Africa (36%), 16 from Eastern Africa (36%), 8 from Central Africa (18%), and 5 from Southern Africa (11%). Policies were published in English (n=22), French (n=18), Portuguese (n=4) and Spanish (n=1). Policy publication dates ranged from 2013 to 2021 and 40% of policies (n=18/45) were published before 2019. Based on the most recent national HIV prevalence reported by UNAIDS[1], 34 countries (76%) had a low prevalence (<5%) and 11 countries (24%) had a high prevalence (≥5%) (online supplementary table 2).

For peer review only - http://bmjopen.bmj.com/site/about/guidelines.xhtml

Overall adoption of WHO-recommended HIV testing strategies

In 2021, 62% of national testing strategies (n=28/45) adopted WHO 2019 recommendations and 38% (n=17/45) were non-adopted (table 1). This represents a 2.4-fold increase in the number of national policies adopting WHO guidance compared to the 2018 review (28 *vs* 9). The subregions with the highest adoption were Southern Africa (80%) and Eastern Africa (75%) whilst Central Africa (63%) and Western Africa (44%) had the lowest adoption rates (table 1; figure 2).

In low-prevalence countries (n=34), 53% of policies (n=18/34) were fully adopted or mostly adopted and 47% (n=16/34) were somewhat not adopted or not adopted. The most common reasons for non-adoption were not using a dual HIV/syphilis RDT in ANC (n=15/34), no retesting prior to ART initiation (n=14/34) and using only two assays to make an HIV-positive diagnosis (n=10/34). In high-prevalence countries (n=11), 91% of policies (n=10/11) were either fully adopted or nearly adopted and 9% were not adopted (1/11). The most common reasons for non-full adoption were the use of only two assays to make an HIV-positive diagnosis (n=10/11), the use of both serial and parallel testing strategies (n=4/11), no use of dual HIV/syphilis RDTs in ANC (n=4/11) and no retesting prior to ART initiation (n=3/11).

When the analysis was restricted to a subset of countries with policies included in the 2018 review that also had an updated policy in the current review (n=28), the proportion adopting WHO guidance in 2018 was 25% (n=7/28) *vs* 71% (n=20/28) in the current review (see online supplementary table 3).

For peer review only - http://bmjopen.bmj.com/site/about/guidelines.xhtml

Table 1. Policy adoption of 2019 WHO-recommended HIV testing strategies in the WHO African region from 2014 to 2021

**AFRO subregions (countries)**	**Policies reviewed**			**Overall adoption**			**Serial testing strategy**			**Discontinuation of tiebreaker* testing strategy**			**Retesting prior to ART initiation**		**Universal 3-test strategy**	**Use of dual HIV/syphilis test****	**No use of Western Blot**
Year	2014	2018	2021	2014	2018	2021	2014	2018	2021	2014	2018	2021	2018	2021	2021	2021	2021
All (n=47)	25 (53%)	32 (68%)	45 (96%)	2 (8%)	9 (28%)	28 (62%)	21 (84%)	26 (81%)	29 (64%)	15 (60%)	26 (81%)	37 (82%)	16 (50%)	23 (51%)	21 (47%)	21 (47%)	35 (78%)
Western (n=17)	6 (35%)	9 (53%)	16 (94%)	0 (0%)	2 (22%)	7 (44%)	5 (83%)	7 (78%)	9 (56%)	4 (67%)	5 (56%)	9 (56%)	1 (11%)	5 (31%)	8 (50%)	7 (44%)	10 (63%)
Central (n=9)	4 (44%)	5 (56%)	8 (89%)	1 (25%)	1 (20%)	5 (63%)	4 (100%)	5 (100%)	7 (88%)	3 (75%)	4 (80%)	7 (88%)	1 (20%)	5 (63%)	3 (38%)	2 (25%)	6 (75%)
Eastern (n=16)	11 (69%)	13 (81%)	16 (100%)	1 (9%)	3 (23%)	12 (75%)	10 (91%)	10 (77%)	12 (75%)	5 (45%)	12 (92%)	16 (100%)	9 (69%)	9 (56%)	9 (56%)	9 (56%)	14 (88%)
Southern (n=5)	4 (80%)	5 (100%)	5 (100%)	0 (0%)	3 (60%)	4 (80%)	2 (40%)	4 (80%)	1 (20%)	3 (75%)	5 (100%)	5 (100%)	5 (100%)	4 (80%)	1 (20%)	3 (60%)	5 (100%)

*Use of a third assay to rule-in HIV infection

**Dual HIV/syphilis RDT as first test in ANC

For peer review only

Adoption of serial testing strategy

In 2021, 64% of national testing strategies (n=29/45) adopted the use of serial testing and 36% (n=16/45) recommended the mix use of serial and parallel testing (figure 3_1). The subregions with the highest adoption were Central (88%) and Eastern Africa (75%), followed by Western Africa (58%); Southern Africa had the lowest adoption rate (20%) (table 1). Most policies recommending serial or parallel testing (n=15/16) conducted simultaneous testing of assay 1 (A1) and assay 2 (A2) in case of discrepant test results, and the Namibian policy recommended parallel testing of A2 and A3 after a reactive A1.

Only 44% of policies (n=20/45) provided guidance on the assay order in relation to their sensitivity and specificity. The proportion of HIV testing strategies/algorithms using 2 consecutive HIV-reactive tests to make an HIV-positive diagnosis was 53% (n=24/45) whereas 47% (n=21/45) recommended the use 3 consecutive HIV-reactive tests.

Sixty-four percent of the HIV testing strategies reviewed (n=29/45) included testing algorithms, i.e., contained specific product names and all of them included WHO prequalified products. Determine HIV 1/2 (Abbott Diagnostics Medical Co., Ltd, Japan) was the most common product used as A1 and Bioline HIV 1/2 3.0 (Abbott Diagnostics Korea Inc., Republic of Korea) or Uni-Gold HIV (Trinity Biotech, Ireland) as A2 or A3 (see online supplementary table 4). Only 31% of policies (n=14/45) mentioned the need to locally verify or validate the HIV testing algorithm.

For peer review only

Discontinuation of a tiebreaker testing strategy

The discontinuation of a third assay to rule in HIV infection (i.e., tiebreaker) was recommended in 82% of national policies (n=37/45) (figure 3_2). The subregions with the highest adoption were Southern Africa and Eastern Africa (100% each) followed by Central Africa (88%), whereas Western Africa had the lowest adoption (56%) (table 1).

Of the eight policies recommending a tiebreaker testing strategy, seven were from Western Africa and one from Central Africa. Seven policies specified the type of A3 to be used, for example, RDT (n=4), RDT or EIA (n=1), nucleic acid testing (NAT: n=2) and LIA (n=1).

Adoption of WHO standard 3-assay testing strategy

The use of three consecutive HIV-reactive tests for the diagnosis of HIV was recommended by 47% of the policies (n=21/45) (figure 3_3). The adoption of this WHO recommendation was highest in Eastern Africa (56%) and Western Africa (50%) and lowest in Southern Africa (20%) (table 1). Most policies recommending a 3-assay testing strategy (n=20/21) were from low HIV-burden countries. Among high HIV-burden countries, only 18% of policies (n=2/11) recommended the use of a 3-assay testing strategy and this corresponded to two countries, Malawi and Zimbabwe, respectively.

Compared to the 2018 review, 10 policies that previously recommended a 2-assay testing strategy transitioned to a 3-assay testing strategy in 2021 (Burkina Faso, Burundi, Chad, Ethiopia, Ghana, Kenya, Lesotho, Malawi, Madagascar, and Zimbabwe, respectively).
Of policies supporting a 3-assay testing strategy, only 53% (n=11/21) also recommended the use of dual HIV/syphilis RDTs among pregnant women.

For peer review only

Adoption of retesting to verify positive status prior to ART initiation

In 2021, 51% of national strategies (n=23/45) recommended retesting prior to ART initiation compared to 50% in 2018 (figure 3_4). The regions with the highest policy adoption were Southern Africa (80%) followed by Central Africa (63%) and Eastern Africa (56%) (table 1). Sixty-five percent of the policies (n=15/23) recommending retesting prior to ART initiation, conduct retesting using the same national HIV testing algorithm in a new specimen run by a different operator in a different testing site; 26% (n=6/23) did not specify how retesting was carried out and 9% of policies (n=2/23) utilized retesting strategies that differed from the national HIV testing algorithm, which included parallel testing of A1/A2 in Botswana and Malawi, respectively.

Discontinuation of Western Blotting

The proportion of national policies not including WB or LIA as part of the HIV testing algorithm was 78% (n=35/45) (figure 3_5). This proportion was highest in Southern Africa (100%) and Easter Africa (88%) followed by Central Africa (75%) and Western Africa (63%) (table 1). Most countries with a supporting policy in favour of WB or LIA (n=9/10) recommended its use to manage persistent inconclusive/indeterminate test results and one policy used WB as A3 (Mauritius).

Adoption of dual HIV/syphilis RDT in ANC settings

Forty-seven percent (n=21/45) of policies supported the use of dual HIV/syphilis RDTs among pregnant women in ANC (figure 3_6). The region was the highest policy uptake was Southern Africa (60%), followed by Eastern Africa (56%) and Western Africa (44%). Central Africa had the lowest policy uptake (25%) (table 1).

For peer review only

The majority of policies (n=20/21) recommended the use of dual HIV/syphilis RDT as A1 among pregnant women and only one policy (Lesotho) recommended its use as a triage test (A0) as an interim algorithm while waiting for the results of a national verification study. The dual HIV/syphilis testing algorithm was not aligned with the national HIV testing algorithm in one country (Zambia): while Determine HIV-1/2 (Abbott Diagnostics Medical Co., Ltd, Japan) and SD Bioline HIV-1/2 3.0 (Abbott Diagnostics Korea Inc, Republic of Korea) products were used as A1 and A2 in the national testing algorithm, Bioline HIV/Syphilis Duo (Abbott Diagnostics Korea Inc, Republic of Korea) and Rapid Test for Antibody to Human Immunodeficiency Virus (HIV) (Colloidal Gold Device) (Beijing Wantai Biological Pharmacy Enterprise Co., Ltd., China) were used as A1 and A2 in the ANC testing algorithm.

In addition to the use in ANC settings, three countries also recommended testing with dual HIV/syphilis RDTs among other populations such as male partners of pregnant women and key populations. Only 50% of policies (n=11/21) specified the brand name of the dual HIV/syphilis RDT in the testing algorithm, and among the policies mentioning the brand name, Bioline HIV/Syphilis Duo (Abbott Diagnostics Korea Inc, Republic of Korea) was the most employed RDT (100%). Only 57% of policies (n=12/21) mentioned whether syphilis treatment was provided immediately after a reactive syphilis test result.

Use of nucleic acid testing (NAT) to diagnose HIV infection

Twenty-two percent of national policies (n=10) recommended the use of NAT as part of the testing algorithm for the diagnosis of HIV infection. Four policies were from Eastern Africa (Malawi, Mauritius, Mozambique and United Republic of Tanzania); three national policies from Southern Africa (Eswatini, Lesotho and Namibia); two policies from Central Africa

For peer review only

(Angola and Sao Tomé et Principe); and one national policy from Western Africa (Guinea Bissau).

Two policies employed NAT as the third assay (A3) of the algorithm while the remaining 8 policies recommended NAT to resolve persistent inconclusive/indeterminate test results (referring either to viral load, PCR or DNA-PCR).

DISCUSSION

The current review found a significant improvement in the overall policy uptake of the latest WHO-recommended HIV testing strategies with 62% of countries in the African region adopting WHO guidance in 2021 compared to 28% of countries in 2018.[10] This represents a 2.4-fold increase in the number of national policies adopting WHO guidance compared to 2018 (28 *vs* 9). However, because a significant proportion (40%) of national policies were published before 2019, country adoption of latest WHO guidance was less likely. Given the rapid changes in HIV testing landscape, this underscores the need of national programmes to update their national HIV testing policies at more regular intervals to keep up with latest WHO guidance.

For peer review only

The recommendations with highest adoption were the discontinuation of a tiebreaker (82%) and western blotting (78%) as well as the use of serial testing (64%). Half of countries (51%) also recommend retesting prior to ART initiation. One likely explanation for the higher policy adoption is that these WHO´s recommendations were published in 2012, 2014 and 2015, respectively (save for discontinuation of western blotting) providing national HIV programmes ample time to incorporate these recommendations into their national HIV testing guidelines. Although moving away from western blotting was recommended more

For peer review only - http://bmjopen.bmj.com/site/about/guidelines.xhtml

recently in 2019,[11] low resource countries in the WHO African region has been at the forefront of implementing HIV RDTs compared to other WHO regions as a way to rapidly expand and increase access to HIV testing services.[12]

Having said that, our review found that a dozen of countries, mostly in Western Africa, still use western blotting as part of the HIV testing algorithms, hindering same-day diagnoses and rapid access to ART and Pre-exposure prophylaxis (PrEP).[11] Despite observing a 1.43 fold increase in the number of countries recommending retesting prior to ART initiation compared to 2018 (23 vs 16), we noted virtually no progress in the proportion of countries with supportive policy, with 51% in 2021 and 50% in 2018.

The WHO´s recommendations with a lower country uptake were the use of a standard 3-assay testing strategy (47%) and the use of dual HIV/syphilis RDT among pregnant women (47%), both recommendations released in 2019.[6]

Before 2019, WHO recommended the use of a 2-assay testing strategy in high HIV prevalence settings (≥5%) and the use of a 3-assay testing strategy in low HIV prevalence settings (<5%) to maintain at least a 99% positive predictive value (PPV).[1] However, as HTS and ART have been scaled up substantially and fewer people undergoing HTS are HIV-positive, the national HTS positivity has also declined, even in high HIV burden settings.[13] Given this changes in the epidemic, WHO now recommends that all settings move toward using a 3-assay testing strategy to ensure high-quality testing.[13] Specifically, in high HIV burden settings in southern Africa (5 countries) and eastern Africa (6 countries), WHO recommends countries still using a 2-assay testing strategy to move toward using a 3-assay testing strategy as their

For peer review only - http://bmjopen.bmj.com/site/about/guidelines.xhtml

treatment-adjusted prevalence, rather than the national HIV prevalence, and national HTS positivity fall below 5%.[13]

In this review we found that only 18% (n=2/11) of the high-burden countries in the WHO African region has a supportive policy on a 3-assay testing strategy. A likely explanation for this slower policy adoption is that national programmes and donors in high burden countries have focused on implementing high-yielding testing approaches,[14, 15] which may have inadvertently let them thinking that HTS positivity was usually >5%, and therefore moving to a 3-assay testing strategy was considered less of a priority. An additional explanation is that countries may perceive that the addition of a third assay to the national 2-assay testing algorithm can be challenging due to additional training and quality assurance requirements, procurement, inventory management and budget implications to the national programme.[16]. While countries will need to develop a plan and identify the optimal time for the transition, in terms of costs, a modelling study found that the total cost of the 3-assay testing strategy was only 2.5% greater than the 2-assay testing strategy at 5% positivity, reflecting that HTS programme cost is primarily determined by the number of A1 conducted.[17] Also, programmatic experience from Ghana transitioning to a 3-assay testing strategy showed that although there were some logistical hiccups related to kit supply and lack of staff for training, it was found feasible and was considered a significant intervention toward assuring high-quality testing as well as saving costs of unnecessary life-long treatment of false HIV-positive clients.[18]

We found that among low-prevalence countries (n=34), a significant proportion (41%) still use a suboptimal testing strategy with 2 assays to make an HIV positive diagnosis, which will

For peer review only - http://bmjopen.bmj.com/site/about/guidelines.xhtml

increase the likelihood of false-positive diagnosis.[19] A retrospective study in Nigeria using household survey data found that the performance of the 2-assay HIV testing algorithm in a low prevalence setting of about 1.4% was poor with a PPV of 94% and a false-positive rate of 5.5%.[16] This PPV is similar to a modelling study assessing the accuracy of the 2015 and 2019 WHO HIV testing algorithms, which was 95.4% using a 2-assay testing strategy.[17]

Considering that the recommendation to use dual HIV/syphilis RDTs in ANC was released only in 2019,[20] its adoption has been rapid with 21 national policies recommending its use. However, the number of countries adopting dual HIV/syphilis RDTs among pregnant women may be in practice higher considering that preliminary NCPI survey data reported that a total of 26 countries in the WHO African region adopted the use of dual HIV/syphilis RDTs in ANC.[21] This inconsistency with our review underscores the difficulties in aggregating accurate policy data in surveys. Some countries may choose not to introduce dual HIV/syphilis RDT. For example, in Malawi where dual HIV/syphilis RDT introduction has been considered but not adopted, the national programme has opted to retain stand-alone HIV and syphilis RDTs in ANC in an effort to prioritize point-of-care test options for HIV and syphilis while maintaining a single HIV testing algorithm in a resource limited context. Dual HIV/syphilis testing is also now recommended for key populations by WHO and this may further increase its use in HIV testing algorithms in national programmes.[22] It is an important option to increase HIV and syphilis detection and treatment and a recent modelling study in Viet Nam shows that it was cost saving or cost-effective.[23]

As countries update their national HIV testing guidelines to incorporate latest WHO guidance and adapt their national HIV testing strategies and algorithms to shift toward using three

For peer review only - http://bmjopen.bmj.com/site/about/guidelines.xhtml

consecutive reactive serology tests to diagnose HIV, introduce dual HIV/syphilis RDTs among pregnant women and key populations, move away from western blotting, reinforcing retesting prior to ART initiation as well as design flexible algorithms to address kit shortages, WHO encourages national programmes to conduct a verification study to provide objective evidence, before national scale-up, that a specific combination of products work well together without sharing false-reactivity, thus reducing the risk of misdiagnosis.[6, 9] To assist countries achieve this, WHO has put together a practical toolkit to optimize HIV testing algorithms;[24] this toolkit contains different resources to select and shortlist HIV products, checklist of laboratory consumables and reagents, budget template for conducting a verification study, generic verification protocol, Ghantt chart to plan the study as well as a database to analyze results and a proposed methodology to conduct rapid implementation and evaluation of the newly selected HIV testing algorithms. Some components of this toolkit have already been used by more than a dozen of countries undertaking this verification study with support from WHO including Armenia, Burkina Faso, Cameroon, Chad, Central African Republic, Cote D´Ivoire, Democratic Republic of Congo, eSwatini, Kenya, Lao, Lesotho, Mali, South Sudan and Zambia. Preliminary results of the verification study have been published by Chad and Cameroon.[25]

While countries update their national HIV testing strategies and wait for the results of a verification study, a practical option to rapidly start implementation without compromising the quality testing is to select interim testing algorithms using WHO prequalified tests as implementing a 3-assay testing strategy without a verification study will ensure a PPV >99%) as opposed to implementing only a validated 2-assay testing strategy that will not achieve the WHO-recommended PPV.

For peer review only

438 **Conclusions**

439 Adoption of WHO-recommended HIV testing strategies has improved in the African region.
440 While WB was only used in a few countries, concerted efforts are needed to phase out this
441 technology in favour of RDTs. Countries should plan to accelerate their transition to WHO
442 recommendations by streamlining efforts to adopt and implement a 3-asssay testing strategy
443 and dual HIV/syphilis RDTs. Conducting verification of testing algorithms, and using
444 appropriate assays can ensure accurate HIV diagnosis in a cost-efficient and time-efficient
445 manner.

446

447 **Acknowledgements**

448 We would like to express our gratitude to WHO colleagues in the respective countries of the
449 WHO African region for their support in locating updated national policy documents. We
450 would like to thank also staff at the GFATM (Youssouf Sawadogo, Ghislaine Grasser and
451 Jacqueline Papo), CHAI (Katherine Guerra, Gillian Leitch, Megan Ginivan and Christian
452 Stillson), Evidence Action Group (Anna Konstantinova and Emilie Efroson) and staff from
453 Ministry of Health in Mozambique (Helga Guambe) and Guinea Bissau (David da Silva Té) for
454 their support in identifying latest national HIV testing policies.

455

456 **Contributors**

457 CJ devised and supervised the review. EF updated the study protocol and conducted the
458 screening, data extraction and analysis. CL acted as a second reviewer and CJ as third
459 reviewer. All authors reviewed the draft, provided critical review and read and approved the
460 final version of the manuscript.

461

For peer review only

Funding

This review was supported by BMGF (INV-024432)

Map disclaimer

The depiction of boundaries on the map(s) in this article do not imply the expression of any opinion whatsoever on the part of BMJ (or any member of its group) concerning the legal status of any country, territory, jurisdiction or area or of its authorities. The map(s) are provided without any warranty of any kind, either express or implied.

For peer review only

Competing interests

The authors declare no competing interests. The contents in this article are those of the authors and do not necessarily reflect the view of the World Health Organization.

Patient consent for publication

Not required.

Ethics approval

This study does not involve human participants and ethical approval was therefore not required.

Data availability statement

All data generated in the study are included in the article or uploaded as supplementary information. National policies may be publicly available. Some policies included in this review may be available through the following websites: (1)

https://aidsfree.usaid.gov/resources/guidance-data/hts. (2) http://www.hivpolicywatch.org/database.htlm. If information on a policy cannot be found through these resources, please contact the authors of this review for additional information.

REFERENCES

1. Consolidated guidelines on HIV testing services, 2015. Geneva: World Health Organization; 2015. Available from: https://www.who.int/publications/i/item/9789241508926 [cited 2022 Feb 24].

2. Guidelines on when to start antiretroviral therapy and on pre-exposure prophylaxis for HIV, 2015. Geneva: World Health Organization; 2015. Available from: https://www.who.int/publications/i/item/9789241509565 [cited 2022 Feb 24].

3. Frescura L, Godfrey-Faussett P, Feizzadeh A A, El-Sadr W, Syarif O, Ghys PD; on and behalf of the 2025 testing treatment target Working Group. Achieving the 95 95 95 targets for all: A pathway to ending AIDS. PLoS One. 2022 Aug 4;17(8):e0272405.

4. Assessment of HIV testing services and antiretroviral therapy service disruptions in the context of COVID-19: lessons learned and way forward in sub-Saharan Africa. World Health Organization; 2021. Available from: https://www.who.int/publications/i/item/9789240039599 [cited 2022 Feb 24].

5. Fact sheet. Latest global and regional statistics on the status of the AIDS epidemic [internet]. Geneva: United Nations Program on HIV/AIDS (UNAIDS); 2021. Available from: https://www.unaids.org/sites/default/files/media_asset/UNAIDS_FactSheet_en.pdf [cited 2022 Feb 24].

6. Consolidated guidelines on HIV testing services, 2019. Geneva: World Health Organization; 2019. Available from: https://www.who.int/publications/i/item/978-92-4-155058-1 [cited 2022 Feb 24].

7. Service delivery approaches to HIV testing and counselling (HTC): a strategic HTC programme framework, 2012. Geneva: World Health Organization; 2012. Available from: https://apps.who.int/iris/handle/10665/75206 [cited 2022 Oct 10].

8. Tippett Barr BA, Lowrance D, Johnson CC, Baggaley RC, Rogers JH, Balachandra SK, Barker J, Kalua T, Bunga S, Low-Beer D, Payne D, Bulterys MG, Jahn A. Treatment-adjusted prevalence to assess HIV testing programmes. Bull World Health Organ. 2021 Dec 1;99(12):874-882.

9. Optimizing HIV testing algorithms: a generic verification protocol for selecting appropriate HIV serology assays and assessing the level of shared false-reactivity. Geneva: World

For peer review only - http://bmjopen.bmj.com/site/about/guidelines.xhtml

Health Organization; 2021. Available from: https://www.who.int/publications/i/item/9789240039162 [cited 2022 Feb 24].

10. Fonner VA, Sands A, Figueroa C, Baggaley R, Quinn C, Jamil MS, Johnson C. Country adherence to WHO recommendations to improve the quality of HIV diagnosis: a global policy review. BMJ Glob Health. 2020 May;5(5):e001939.

11. WHO recommends countries move away from the use of western blotting and line immunoassays in HIV testing strategies and algorithms. Geneva: World Health Organization; 2019. Available from: https://www.who.int/publications/i/item/WHO-CDS-HIV-19.30 [cited 2022 Oct 10].

12. Flynn DE, Johnson C, Sands A, Wong V, Figueroa C, Baggaley R. Can trained lay providers perform HIV testing services? A review of national HIV testing policies. BMC Res Notes. 2017 Jan 4;10(1):20. doi: 10.1186/s13104-016-2339-1. PMID: 28057054; PMCID: PMC5216526.

13. WHO encourages countries to adapt HIV testing strategies in response to changing epidemic. Geneva: World Health Organization; 2019. Available from: https://www.who.int/publications/i/item/WHO-CDS-HIV-19.34 [cited 2022 Oct 10].

14. Jubilee M, Park FJ, Chipango K, Pule K, Machinda A, Taruberekera N. HIV index testing to improve HIV positivity rate and linkage to care and treatment of sexual partners, adolescents and children of PLHIV in Lesotho. PLoS One. 2019 Mar 27;14(3):e0212762.

15. Ahmed S, Schwarz M, Flick RJ, Rees CA, Harawa M, Simon K, Robison JA, Kazembe PN, Kim MH. Lost opportunities to identify and treat HIV-positive patients: results from a baseline assessment of provider-initiated HIV testing and counselling (PITC) in Malawi. Trop Med Int Health. 2016 Apr;21(4):479-85.

16. Patel HK, Ikpe S, Bronson M, Birhanu S, Abimiku A, Jahun I, Detorio M, Lupoli K, Yavo D, Bassey OO, Jelpe TD, Kagurusi B, Iriemenam NC, et al. (2022) Performance of HIV rapid testing algorithm in Nigeria: Findings from a household-based Nigeria HIV/AIDS Indicator and Impact Survey (NAIIS). PLOS Glob Public Health 2022 2(7): e0000466.

17. Eaton JW, Sands A, Barr-DiChiara M, Jamil MSJ, Baggaley R, Tippett Barr BA, Kalua T, Jahn A, Maheu-Giroux, Johnson. Accuracy and performance of the WHO 2015 and WHO 2019 HIV testing strategies across epidemic settings. medRxiv 2021.03.31.21254700. Available from: https://www.medrxiv.org/content/10.1101/2021.03.31.21254700v1. [cited 2022 Oct 10].

18. Rowland Adukpo. Experience from Ghana in transitioning to a 3-assay HIV testing strategy. WHO satellite session "Improving quality of HIV diagnosis: do you know how to update your HIV national testing algorithms? At the African Society of Laboratory Medicine Conference, 18 Nov 2021. Available from: https://www.who.int/news-room/events/detail/2021/11/18/default-calendar/improving-quality-of-hiv-diagnosis-toolkit-launch [cited 2022 Oct 10].

For peer review only - http://bmjopen.bmj.com/site/about/guidelines.xhtml

19. Johnson CC, Fonner V, Sands A, Ford N, Obermeyer CM, Tsui S, Wong V, Baggaley R. To err is human, to correct is public health: a systematic review examining poor quality testing and misdiagnosis of HIV status. J Int AIDS Soc. 2017 Aug 29;20(Suppl 6):21755.

20. Dual HIV/syphilis rapid diagnostic tests can be used as the first test in antenatal care. Geneva: World Health Organization; 2019. Available from: https://www.who.int/publications/i/item/WHO-CDS-HIV-19.38. [cited 2022 Oct 10].

21. UNAIDS Joint United Nations Programme on HIV/AIDS WH. Laws and policies analytics. Available from: https://lawsandpolicies.unaids.org/topicresult?i=13&o=219,397&lan=en. [cited 2022 Oct 10].

22. Consolidated guidelines on HIV, viral hepatitis and STI prevention, diagnosis, treatment and care for key populations. Geneva: World Health Organization; 2022. Available from: https://www.who.int/publications/i/item/9789240052390. [cited 2022 Oct 10].

23. Coomes D, Green D, Barnabas R, Sharma M, Barr-DiChiara M, Jamil MS, Baggaley R, Owiredu MN, Macdonald V, Nguyen VTT, Vo SH, Taylor M, Wi T, Johnson C, Drake AL. Cost-effectiveness of implementing HIV and HIV/syphilis dual testing among key populations in Viet Nam: a modelling analysis. BMJ Open. 2022 Aug 11;12(8):e056887.

24. Toolkit to optimize HIV testing algorithms. Geneva: World Health Organization; 2021. Available from: https://www.who.int/tools/optimizing-hiv-testing-algorithms-toolkit. [cited 2022 Oct 11].

25. Lastrucci C Abderrazzack AF, Anoubissi JD, Adawaye C, Fokam J, Uwimbabazi JC, Etogo O, Kembou E, Sands A, Djemadji-Oudjiel N, Johnson CC. Développement d´algorithmes de dépistage VIH performants: résultats des études de verification au Tchad et au Cameroun. 11e Conférece international franbcophone, AFRAVIH 2022. 6 - 9 avril 2022. Marseille, France. Poster number PJ163. Available from: http://pslsh.td/wp-content/uploads/2022/04/template-AFRAVIH-2022_poster-developpement-algorithmes-depistage-VIH_Tchad-Cameroun_draft_V2-draftEFAB.pdf [cited 2022 Oct 11].

For peer review only

623 **Figure legends**

624

625 **Figure 1.** Countries from the WHO African Region with HIV testing policies identified (in blue) and included in the analysis (n=45/47). The two countries not included in the review are indicated in the map.

628

629 **Figure 2.** Overall adoption of 2019 WHO HIV testing strategies in Africa by subregion in 2021.

630

631 **Figure 3.** Policy adoption in 2021 of six specific WHO HIV testing recommendations in the WHO African Region.

For peer review only

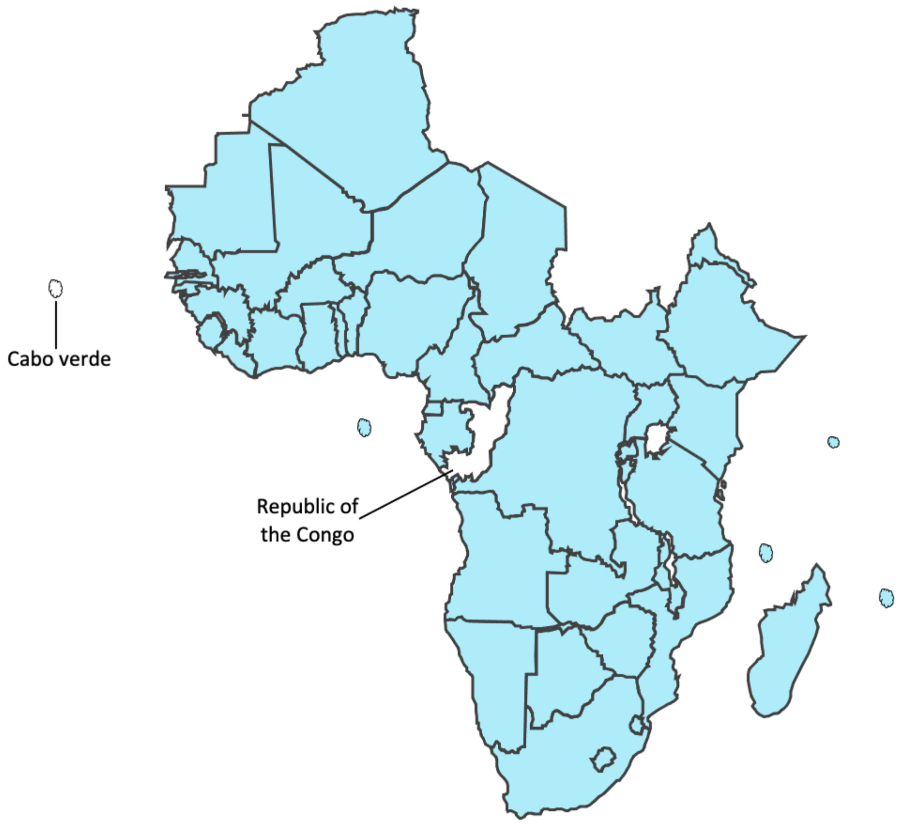

187x162mm (144 x 144 DPI)

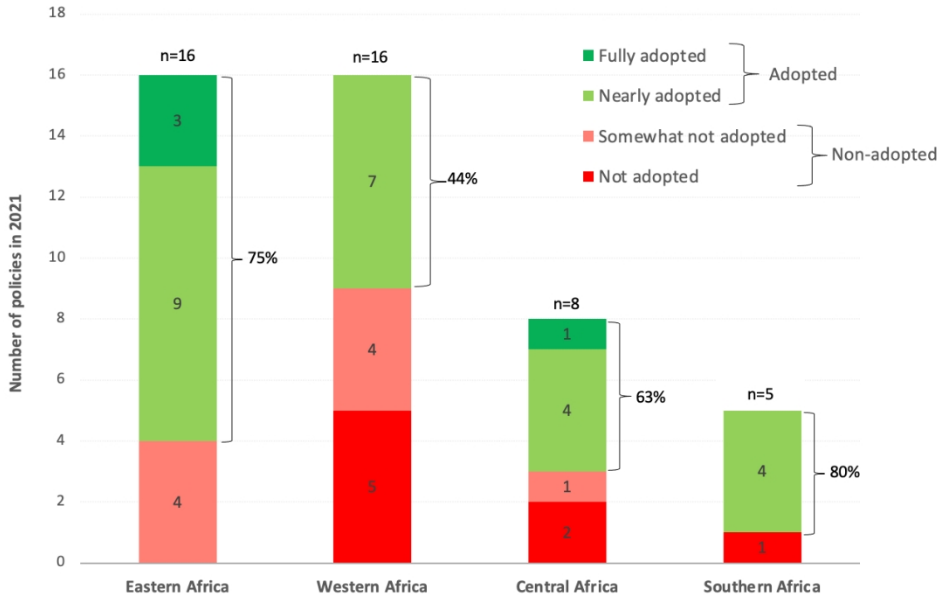

237x154mm (144 x 144 DPI)

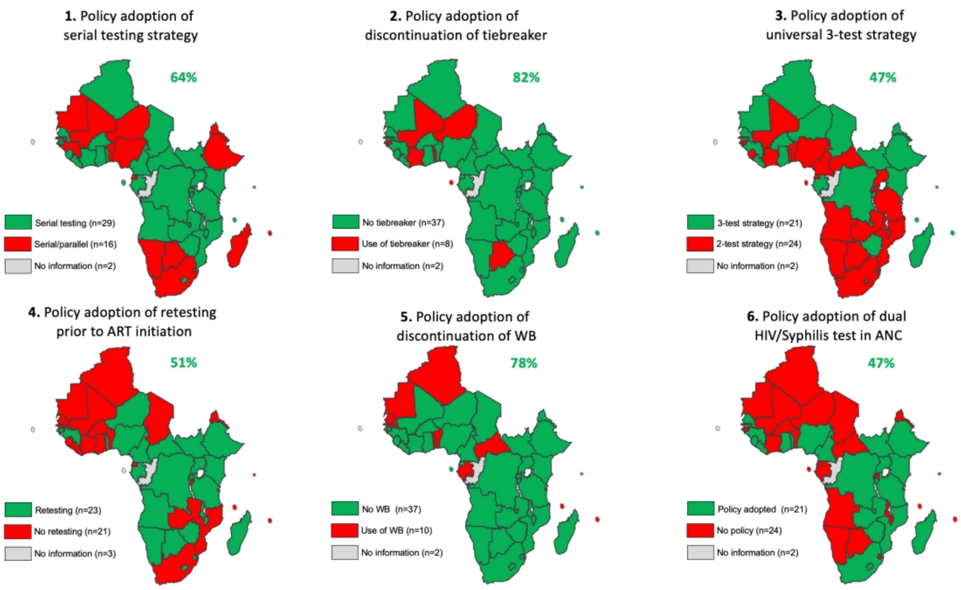

350x207mm (144 x 144 DPI)

Supplementary Appendix

Country adoption of WHO 2019 guidance on HIV testing strategies and algorithms: a policy review across the WHO African region

Emmanuel Fajardo,[1] Céline Lastrucci,[1] Nayé Bah,[2] Casimir Manzengo Mingiedi,[3] Ndoungou Salla Ba,[4] Fausta Shakiwa Mosha,[5] Frank John Lule,[6] Margaret Alia Sampson Paul,[6] Lago Hughes,[6] Magdalena Barr-DiChiara,[1] Muhammad Shahid Jamil,[1] Anita Sands,[7] Rachel Baggeley,[1] Cheryl Johnson[1]

Supplementary Figure 1. Countries in the WHO African Region and grouping by subregion

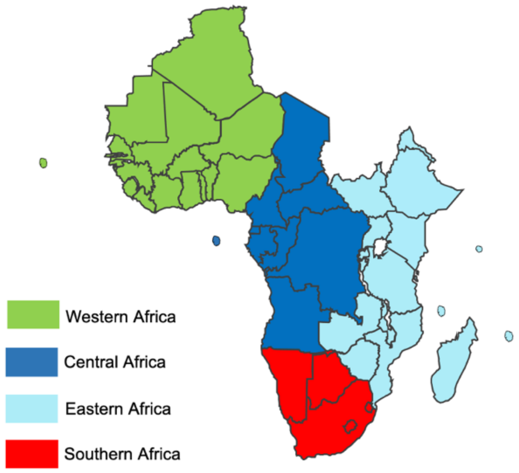

WHO African Region (n=47)			
Western Africa (n=17)	Central Africa (n=9)	Eastern Africa (n=16)	Southern Africa (n=5)
Algeria	Angola	Burundi	Botswana
Cabo Verde	Cameroon	Comoros	eSwatini
Burkina Faso	CAR	Eritrea	Lesotho
Benin	Chad	Ethiopia	Namibia
Ghana	DRC	Kenya	South Africa
Guinea	Equotorial Guinea	Madagascar	
Guinea-Bissau	Gabon	Malawi	
Ivory Coast	Republic of the Congo	Mauritius	
Liberia	Sao Tome and Principe	Mozambique	
Mauritania		Rwanda	
Mali		Seychelles	
Niger		South Sudan	
Nigeria		Tanzania	
Senegal		Uganda	
Sierra Leone		Zambia	
The Gambia		Zimbabwe	
Togo			

For peer review only

Supplementary Table 1. Categories for data extraction

**1. Policy Information**	
▪ Country<br>▪ WHO region<br>▪ HIV testing policy identified?	▪ Year of policy publication<br>▪ Type of policy document<br>▪ National HIV prevalence
**2. HIV testing algorithm (after 18 months of age)**	
▪ HIV testing algorithm provided?<br>▪ Image or text format?<br>▪ Type of scenario in which algorithm is used<br>▪ Number of assays used in algorithm<br>▪ Serial or parallel strategy<br>▪ Use of tiebreaker?<br>▪ Use of Western Blot or LIA?<br>▪ Mention of test order relating to sens/spec?<br>▪ High or low prevalence strategy used?<br>▪ Type of tests mentioned?<br>▪ Brand of tests mentioned?<br>▪ Are assays pre-qualified by WHO?<br>▪ A1 test name<br>▪ A1 type of assay	▪ A1 sens-/spec<br>▪ A2 test name<br>▪ A2 type of assay<br>▪ A2 sens-/spec<br>▪ A3 test name<br>▪ A3 type of assay<br>▪ A3 sens-/spec<br>▪ Source of assay sens/spec<br>▪ Mention of in-country assay validation<br>▪ Alignment with WHO policy?<br>▪ Primary reason(s) algorithm fails to meet WHO recommendations<br>▪ Other notes about testing strategy
**3. HIV testing algorithm among pregnant women using dual HIV/Syphilis RDTs**	
▪ HIV testing algorithm provided?<br>▪ Image or text format?<br>▪ Type of scenario in which algorithm is used<br>▪ Type of scenario in which algorithm should not be used (women on ART, already diagnosed/treated for syphilis, retesting for HIV)<br>▪ What is there syphilis testing strategy for women with HIV?<br>▪ Is dual test used as A1 or A0?<br>▪ Is the dual test algorithm aligned with the national HIV testing algorithm?<br>▪ Is the dual test algorithm aligned with the national syphilis testing algorithm?<br>▪ Brand of dual test mentioned?	▪ Is dual test prequalified by WHO?<br>▪ Dual test name<br>▪ Mention of penicillin treatment for reactive TP?<br>▪ Mention of further testing for reactive TP?<br>▪ Mention of in-country assay validation<br>▪ Alignment with WHO policy?<br>▪ Primary reason(s) algorithm fails to meet WHO recommendations<br>▪ Other notes about testing strategy
**4. Retesting prior to ART initiation**	
▪ Retest required before starting ART?<br>▪ Retest strategy same as national algorithm?<br>▪ Notes about retesting strategy	

For peer review only

Supplementary Table 2. List of national policies on HIV testing services in the WHO African Region collected in 2018 and 2021 by subregion

Subregion	Count	Country	Policy reviewed in 2018?	New policy or algorithm identified?	Publication year	Type of document	Language	Algorithm provided?	National HIV prevalence
Central Africa	1	Angola	Yes, 2015	Yes	2020	HTS	Portuguese	Yes	Low
	2	Cameroon	Yes, 2015	Yes	2019	ART	French	Yes	Low
	3	CAR	Yes, 2010	Yes	2018	ART	French	Yes	Low
	4	Chad	Yes, 2011	Yes	2017	HTS	French	Yes	Low
	5	DRC	Yes, 2017	Yes	2020	ART	French	Yes	Low
	6	Equatorial Guinea	No	Yes	2018	ART	Spanish	Yes	Low
	7	Gabon	No	Yes	2017	HTS	French	Yes	Low
	8	Republic of the Congo	No	**No**	–	–	–	–	–
	9	São Tomé e Principe	No	Yes	2018	Algorithm	Portuguese	Yes	Low
Eastern Africa	10	Burundi	Yes, 2016	Yes	2020	HTS	French	Yes	Low
	11	Comoros	Yes, 2007	Yes	2016	HTS	French	Yes	Low
	12	Eritrea	No	Yes	2019	HTS	English	Yes	Low
	13	Ethiopia	Yes, 2017	Yes	2018	ART	English	Yes	Low
	14	Kenya	Yes, 2017	Yes	2021	HTS	English	Yes	Low
	15	Madagascar	Yes, 2011	Yes	2018	HTS	French	Yes	Low
	16	Malawi*	Yes, 2016	Yes*	2016	HTS	English	Yes	High
	17	Mauritius	No	Yes	2020	HTS	English	Yes	Low
	18	Mozambique	Yes, 2016	Yes	2020	PMTCT	Portuguese	Yes	High
	19	Rwanda	Yes, 2016	Yes	2018	HTS	English	Yes	Low
	20	Seychelles	No	Yes	2019	HTS	English	Yes	Low
	21	South Sudan	Yes, 2017	Yes	2020	ART	English	Yes	Low
	22	Tanzania	Yes, 2017	Yes	2021	HTS	English	Yes	High
	23	Uganda	Yes, 2016	Yes	2020	ART	English	Yes	High
	24	Zambia	Yes, 2018	Yes	2020	HTS	English	Yes	High
	25	Zimbabwe*	Yes, 2016	Yes*	2016	Other	English	Yes	High
Southern Africa	26	Botswana**	Yes, 2016	**No**	2016	ART	English	Yes	High
	27	eSwatini**	Yes, 2018	**No**	2018	ART	English	Yes	High
	28	Lesotho	Yes, 2016	Yes	2021	PMTCT	English	Yes	High

For peer review only - http://bmjopen.bmj.com/site/about/guidelines.xhtml

	29	Namibia	Yes, 2016	Yes	2018	HTS	English	Yes	High
	30	South Africa	Yes, 2016	Yes	2020	ART	English	Yes	High
Western Africa	31	Algeria**	Yes, 2013	**No**	2013	HTS	French	Yes	Low
	32	Benin	No	Yes	2017	HTS	French	Yes	Low
	33	Burkina Faso	Yes, 2008	Yes	2021	ART	French	Yes	Low
	34	Cabo Verde	No	**No**	–	–	–	–	–
	35	Ghana	Yes, 2014	Yes	2019	ART	English	Yes	Low
	36	Guinea	No	Yes	2019	HTS	French	Yes	Low
	37	Guinea-Bissau	No	Yes	2021	ART	Portuguese	Yes	Low
	38	Ivory Coast**	Yes, 2016	**No**	2016	HTS	French	Yes	Low
	39	Liberia	Yes, 2015	Yes	2020	HTS	English	Yes	Low
	40	Mauritania	No	Yes	2020	HTS	French	Yes	Low
	41	Mali	No	Yes	2017	HTS	French	Yes	Low
	42	Niger	No	Yes	2020	HTS	French	Yes	Low
	43	Nigeria	Yes, 2016	Yes	2020	ART	English	Yes	Low
	44	Senegal	Yes, 2017	Yes	2018	HTS	French	Yes	Low
	45	Sierra Leone	Yes, 2017	Yes	2020	ART	English	Yes	Low
	46	The Gambia	Yes, 2014	Yes	2019	ART	English	Yes	Low
	47	Togo	No	Yes	2019	HTS	French	Yes	Low

*These countries provided an updated HIV testing algorithm despite having an old HIV testing guideline

** These countries did not provide an update HIV testing guideline but were included in the analysis

Supplementary Table 3. Progress on policy adoption in the WHO African Region from 2018 to 2021

Subregion	Count	Country	Policy reviewed in 2018?	Adherence in 2018	Policy reviewed in 2021	Adherence in 2021
Central Africa	1	Angola	Yes, 2015	Not adopted	2020	Mostly adopted
	2	Cameroon	Yes, 2015	Not adopted	2019	Mostly adopted
	3	CAR	Yes, 2010	Not adopted	2018	Not adopted
	4	Chad	Yes, 2011	Not adopted	2017	Mostly adopted
	5	DRC	Yes, 2017	Adopted	2020	Adopted
	6	Equatorial Guinea	No	–	2018	Not adopted
	7	Gabon	No	–	2017	Mostly adopted
	8	Republic of the Congo	No	–	–	–
	9	São Tomé e Principe	No	–	2018	Not adopted
Eastern Africa	10	Burundi	Yes, 2016	Not adopted	2020	Mostly adopted
	11	Comoros	Yes, 2007	No information	2016	Not adopted
	12	Eritrea	No	–	2019	Not adopted
	13	Ethiopia	Yes, 2017	No information	2018	Mostly adopted
	14	Kenya	Yes, 2017	Mostly adopted	2021	Adopted
	15	Madagascar	Yes, 2011	No information	2018	Mostly adopted
	16	Malawi	Yes, 2016	Not adopted	2016	Mostly adopted
	17	Mauritius	No	–	2020	Not adopted
	18	Mozambique	Yes, 2016	Not adopted	2020	Mostly adopted
	19	Rwanda	Yes, 2016	Not adopted	2018	Mostly adopted
	20	Seychelles	No	–	2019	Not adopted
	21	South Sudan	Yes, 2017	Not adopted	2020	Adopted
	22	Tanzania	Yes, 2017	Not adopted	2021	Mostly adopted
	23	Uganda	Yes, 2016	Mostly adopted	2020	Mostly adopted
	24	Zambia	Yes, 2018	Not adopted	2020	Not adopted
	25	Zimbabwe	Yes, 2016	Adopted	2016	Adopted
Southern Africa	26	Botswana	Yes, 2016	Mostly adopted	2016	Not adopted
	27	eSwatini	Yes, 2018	Not adopted	2018	Not adopted
	28	Lesotho	Yes, 2016	Adopted	2021	Mostly adopted
	29	Namibia	Yes, 2016	No information	2018	Not adopted
	30	South Africa	Yes, 2016	Mostly adopted	2020	Not adopted
Western Africa	31	Algeria	Yes, 2013	Adopted	2013	Not adopted
	32	Benin	No	–	2017	Not adopted
	33	Burkina Faso	Yes, 2008	Not adopted	2021	Mostly adopted
	34	Cabo Verde	No	–	–	–
	35	Ghana	Yes, 2014	Not adopted	2019	Mostly adopted
	36	Guinea	No	–	2019	Mostly adopted
	37	Guinea-Bissau	No	–	2021	Not adopted
	38	Ivory Coast	Yes, 2016	Not adopted	2016	Not adopted
	39	Liberia	Yes, 2015	Not adopted	2020	Mostly adopted
	40	Mauritania	No	–	2020	Not adopted
	41	Mali	No	–	2017	Not adopted
	42	Niger	No	–	2020	Not adopted
	43	Nigeria	Yes, 2016	Not adopted	2020	Mostly adopted
	44	Senegal	Yes, 2017	Mostly adopted	2018	Not adopted
	45	Sierra Leone	Yes, 2017	Not adopted	2020	Not adopted
	46	The Gambia	Yes, 2014	Not adopted	2019	Not adopted
	47	Togo	No	–	2019	Not adherent

Those highlighted in red are policies reviewed in 2018 that were updated (n=28). Number of policies deemed adopted in 2018 (n=7) and number deemed adopted in 2021 (n=20)

For peer review only - http://bmjopen.bmj.com/site/about/guidelines.xhtml

Supplementary Table 4. Short name of products used as part of the 2-assay or 3-assay HIV testing algorithm in 28 HIV national testing policy documents reviewed in 2021

Strategy	Count	Country	Assay 1	Assay 2	Assay 3
2-assay	1	Angola	Determine	Uni-Gold	–
	2	Cameroon	Determine or Uni-Gold	OraQuick or Shanghai	–
	3	CAR	Determine	Uni-Gold	–
	4	Cote D'Ivoire	Determine	SD Bioline or GenieFast	StatPak or EIA (as tiebreaker)
	5	Equatorial Guinea	Determine	Hexagon	Uni-Gold (for discordants)
	6	eSwatini	Determine	Uni-Gold	–
	7	Gambia	Determine	SD Bioline or First Response	–
	8	Guinea Bissau	Determine	First Response	PCR (as tiebreaker)
	9	Mali	Alere Combo	SD Bioline	First Response (as tiebreaker)
	10	Mozambique	Determine	Uni-Gold	–
	11	Rwanda	Alere Combo	StatPak	–
	12	São Tomé e Principe	Determine	SD Bioline	PCR (as tiebreaker)
	13	Sierra Leone	Determine	SD Bioline	Uni-Gold (as tiebreaker)
	14	Uganda	Determine	StatPak	SD Bioline (for discordants)
	15	Nigeria	Determine<br>Uni-Gold<br>Stat-Pak<br>Double Check Gold<br>Sure Check<br>HIV Quick Check	Determine<br>Uni-Gold<br>Stat-Pak<br>Double Check Gold<br>Sure Check<br>HIV Quick Check	Determine<br>Uni-Gold<br>Stat-Pak<br>Double Check Gold<br>Sure Check<br>HIV Quick Check<br>(as tiebreaker)
	16	Togo	Murex HIV Ag/Ab<br>Apdia HIV Ag/Ab<br>Determine<br>Alere Combo<br>Vikia HIV 1/2<br>Wanta Rapid Test<br>ABON HIV 1/2/0<br>Standard Q<br>Hexagon<br>Genie Fast<br>SD Bioline<br>First Response<br>SD Bioline HIV/Syphilis<br>OraQuick HIV Self-Test	ABON HIV 1/2/0<br>Standard Q<br>SD Bioline<br>First Response<br>HIV Tri-Dot	INNOLIA or Genius (as tiebreaker)
3-assay	17	Burundi	Alere Combo or Determine	SD Bioline or Uni-Gold	Wondfo One Step
	18	Gabon	Alere Combo	Determine	SD Bioline
	19	Ghana	First Response	OraQuick	SD Bioline
	20	Guinea	Determine	SD Bioline	Multisure
	21	Kenya	INSTI	Uni-Gold	SD Bioline
	22	Liberia	Determine	SD Bioline	Uni-Gold
	23	Madagascar	Determine	Uni-Gold	SD Bioline
	24	Mauritius	GenScreen Ultra Ag/Ab	Alere Combo	Western Blot
	25	Malawi	INSTI	Uni-Gold	SD Bioline

For peer review only - http://bmjopen.bmj.com/site/about/guidelines.xhtml

	26	Niger	Alere Combo	Wondfo One Step	SD Bioline
	27	Senegal	Determine	SD Bioline	Multisure
	28	Zimbabwe	Determine	Chembio	INSTI
	29	Burkina Faso	3rd generation:<br>Determine<br>Double check Gold Ultra<br>Onsite HIV1+2 Plus Combo<br>VIKIA HIV 1/2<br>4th generation:<br>Alere Ag/Ab Combo<br>OnSite HIV Ab/Ag (CTK)	HIV TriDot<br>ImmunoFlow<br>OnSite HIV 1/2 Ab Plus (CTK)<br>SD Bioline	Any of those not selected as A1 or A2

For peer review only

BMJ Open

Country adoption of WHO 2019 guidance on HIV testing strategies and algorithms: a policy review across the WHO African region

Journal:	*BMJ Open*
Manuscript ID	bmjopen-2022-071198.R1
Article Type:	Original research
Date Submitted by the Author:	15-Sep-2023
Complete List of Authors:	Fajardo, Emmanuel; World Health Organization, Global HIV, Hepatitis, and STI Programmes<br>Lastrucci, Céline; WHO, Global HIV, Hepatitis, and STI Programmes<br>Bah, Nayé; World Health Organization Regional Office for Africa<br>Mingiedi, Casimir Manzengo; World Health Organization Regional Office for Africa, Inter-country support team for Central Africa<br>Ba, Ndoungou Salla; World Health Organization Regional Office for Africa, Inter-country support team for Western and Central Africa<br>Mosha, Fausta; World Health Organization Regional Office for Africa, Inter-country support team for Eastern and Southern Africa<br>Lule, Frank John; World Health Organization Regional Office for Africa<br>Paul, Margaret Alia Sampson; World Health Organization Regional Office for Africa<br>Hughes, Lago; World Health Organization Regional Office for Africa<br>Barr-DiChiara, Magdalena; World Health Organization, Department of Global Programmes of HIV, Hepatitis and HIV<br>Jamil, Muhammad; World Health Organization, Global HIV, Hepatitis, and STI Programmes<br>Sands, Anita ; World Health Organization, Regulation and Prequalification<br>Baggaley, R; World Health Organization<br>Johnson, Cheryl; WHO
**Primary Subject Heading**:	Health policy
Secondary Subject Heading:	HIV/AIDS
Keywords:	Health policy < HEALTH SERVICES ADMINISTRATION & MANAGEMENT, HIV & AIDS < INFECTIOUS DISEASES, Diagnostic microbiology < INFECTIOUS DISEASES

SCHOLARONE™
Manuscripts

I, the Submitting Author has the right to grant and does grant on behalf of all authors of the Work (as defined in the below author licence), an exclusive licence and/or a non-exclusive licence for contributions from authors who are: i) UK Crown employees; ii) where BMJ has agreed a CC-BY licence shall apply, and/or iii) in accordance with the terms applicable for US Federal Government officers or employees acting as part of their official duties; on a worldwide, perpetual, irrevocable, royalty-free basis to BMJ Publishing Group Ltd ("BMJ") its licensees and where the relevant Journal is co-owned by BMJ to the co-owners of the Journal, to publish the Work in this journal and any other BMJ products and to exploit all rights, as set out in our licence.

The Submitting Author accepts and understands that any supply made under these terms is made by BMJ to the Submitting Author unless you are acting as an employee on behalf of your employer or a postgraduate student of an affiliated institution which is paying any applicable article publishing charge ("APC") for Open Access articles. Where the Submitting Author wishes to make the Work available on an Open Access basis (and intends to pay the relevant APC), the terms of reuse of such Open Access shall be governed by a Creative Commons licence – details of these licences and which Creative Commons licence will apply to this Work are set out in our licence referred to above.

Other than as permitted in any relevant BMJ Author's Self Archiving Policies, I confirm this Work has not been accepted for publication elsewhere, is not being considered for publication elsewhere and does not duplicate material already published. I confirm all authors consent to publication of this Work and authorise the granting of this licence.

For peer review only

Title: Country adoption of WHO 2019 guidance on HIV testing strategies and algorithms: a policy review across the WHO African region

Authors: Emmanuel Fajardo,[1] Céline Lastrucci,[1] Nayé Bah,[2] Casimir Manzengo Mingiedi,[3] Ndoungou Salla Ba,[4] Fausta Shakiwa Mosha,[5] Frank John Lule,[6] Margaret Alia Samson Paul,[6] Lago Hughes,[6] Magdalena Barr-DiChiara,[1] Muhammad Shahid Jamil,[1] Anita Sands,[7] Rachel Baggaley,[1] Cheryl Johnson[1]

Authors affiliations:

[1]Global HIV, Hepatitis and STI Programmes, WHO, Switzerland

[2]Regional Office for Africa, WHO Country Office, Mali

[3]Regional Office for Africa, Inter-country support team for Central Africa, WHO, Gabon

[4]Regional Office for Africa, Inter-country support team for Western and Central Africa, WHO, Burkina Faso

[5]Regional Office for Africa, Inter-country support team for Eastern and Southern Africa, WHO Zimbabwe

[6]Regional Office for Africa, WHO Congo Brazzaville

[7]Incidents and Substandard/Falsified Medical Products Team, Regulation and Prequalification Department, WHO, Switzerland

Corresponding author: Cheryl Johnson, Global HIV, Hepatitis and STI Programmes, WHO, Avenue Appia 20, 1211 Geneva, Switzerland. johnsonc@who.int

ABSTRACT

Objectives: In 2019, the World Health Organization (WHO) released guidelines on HIV testing services (HTS). We aim to understand adoption of these recommendations.

Design: Policy review

Setting: 47 countries within the WHO African region.

Participants: National HTS policies from the WHO African region as of December 2021.

Primary and secondary outcome measures: Uptake of WHO recommendations across national HTS policies including the standard 3-test strategy; discontinuation of a tie-breaker test to rule in HIV infection; discontinuation of western blotting (WB) for HIV diagnosis; use of retesting prior to ART initiation; and the use of dual HIV/syphilis rapid diagnostic tests (RDTs) in antenatal care (ANC). Country policy adoption was assessed on a continuum, based on varying levels of complete adoption.

Results: National policies were reviewed for 96% (n=45/47) of countries in the WHO African region, 40% (n=18) were published before 2019, and 62% (n=28) adopted WHO guidance. As of 2021, adoption in the region was 62% (n=28/45). Among countries that had not fully adopted WHO guidance, using a 2-test strategy was the most common reason for misalignment; 31% (n=14) and 22% (n=10) in low (<5%) and high ([illegible]5%) prevalence countries, had not yet adopted the 3-test strategy. Ten policies (22%) recommended the use of WB in their HIV testing algorithm, and 51% (n=23) recommended retesting before ART initiation. Dual HIV/syphilis RDTs were recommended in 47% (n=21/45) of policies.

Conclusions: Many countries in the African region have adopted WHO-recommended HIV testing strategies. While WB was only used in a few countries, concerted efforts are needed to phase out this technology in favour of RDTs. Countries should accelerate their transition to

For peer review only - http://bmjopen.bmj.com/site/about/guidelines.xhtml

WHO recommendations by streamlining efforts to adopt and implement a 3-test strategy and dual HIV/syphilis RDTs.

Keywords: HIV, diagnosis, rapid test, dual HIV/syphilis, policy, guideline, Africa, algorithm

Strengths and limitations of this study

- This study provided a comprehensive review of existing national HTS policies in the African region and was able to identify current policies for 45 countries. While this represents nearly all countries in the region, we were unable to identify policies for two countries.
- Data was collected and analyses using a robust search and global repository developed by WHO, as well as a standardised extraction tool to synthesise national policy information. Translation of policies was conducted by the review team.
- The review focused only on published policies and did not assess country implementation at the national, subnational, or site level. Thus, it is possible that some practices in country differ than policies reviewed, and level of adoption reported may vary.
- The review focused on WHO recommendations related to HIV testing strategies for individuals ≥18 months of age. HIV testing strategy recommendations, such as early infant diagnosis, was not included.
- The review also identified countries which reported that they were in the process of adopting WHO recommendations – particularly the use of 3-test strategy and dual HIV/syphilis RDTs. Thus, our estimates may be conservative an under-estimate policy adoption in the African region.

INTRODUCTION

HIV Testing Services (HTS) is the critical gateway to accessing HIV-related care and treatment for those diagnosed as HIV-positive and as a means to accessing prevention services for those who test HIV-negative.[1, 2] Despite considerable progress, reaching the UNAIDS 95-95-95 targets by 2025 will require increased testing uptake, particularly among populations with testing gaps such as key population, youth and men.[3] Further, the global emergency caused by the COVID-19 pandemic in 2020 has disrupted many health services including HIV testing services, and supply chains of key commodities such as rapid diagnostic tests (RDTs) have been stretched.[4] In 2021, it was estimated that 85% of people living with HIV globally were aware of their serostatus.[5]

In 2019, the World Health Organization (WHO) published consolidated guidelines on HIV testing services (HTS),[6] bringing together existing recommendations specific to testing strategies and algorithms e.g., use of serial testing rather than parallel testing (WHO, 2012),[7] discontinuation of a tiebreaker strategy to rule-in HIV infection (WHO, 2015)[1] and, retesting prior to antiretroviral treatment (ART) initiation, recommended since 2014;[1] and released three new recommendations related to testing strategies namely: (1) moving toward a universal 3-assay testing strategy using three consecutive HIV-reactive tests to provide and HIV-positive diagnosis, (2) discontinuation of western blot (WB) and line immunoassays (LIA) and, (3) use of dual HIV/syphilis RDTs as first assay in HTS in antenatal care (ANC) settings. In light of these testing modalities, ensuring that HIV testing is accessible and adheres to the "5 C's" including voluntary consent, confidentiality, counselling, correct results, and linkage to care, is of upmost importance.

These recommendations have been prioritised because they are essential to achieving global 95-95-95 goals by making testing accurate, affordable and high impact. [3,6] By moving away from testing services with WB, programmes will no longer have long turnaround times which delay the ability to offer same day ART or PrEP initiation. [6] Adoption of innovative tools such as the dual HIV/syphilis RDTs will enable more people to be tested and treated for syphilis, which is essential for achieving triple elimination goals. [6] Additionally, many countries still use the 2015 WHO recommendation of using the national HIV prevalence to determine whether a two-assay (≥5%) or three-assay testing strategy (<5%) should be used.[1] However, as ART coverage has expanded HIV positivity and the proportion of people with HIV who are undiagnosed and not in care will continue to decline. As a result, the positive predictive value of previous testing strategies will also decline and lead to an increase in false positive diagnoses. In relation to the guideline development process, WHO now recommends all countries use a standard 3-test strategy.[8] WHO also recommends that countries planning to update their HIV testing algorithms undertake a verification study to select appropriate HIV serology products and ensure they don't cross-react in order to minimize the risk of misdiagnosis.[9]

Box 1. WHO recommendations for HIV testing strategies for individuals ≥18 months of age, 2019

- Western blotting and line immunoassays should not be used in national HIV testing strategies and algorithms.
- Dual HIV/syphilis RDTs can be the first test in HIV testing strategies and algorithms in ANC settings.

- In response to changes in the HIV epidemic, WHO recommends countries use three consecutive reactive tests to provide an HIV-positive diagnosis.
- Testing strategies should use tests serially, not in parallel, and should not use a tiebreaker to rule-in HIV infection. Instead, those with discrepant test results should be ruled inconclusive and referred for further testing in 14 days to rule-in or rule-out seroconversion.
- WHO recommends that all HIV testing algorithms use a combination of RDTs and/or EIAs to achieve at least 99% positive predictive value and use a combination of tests with ≥99% sensitivity and ≥98% specificity. WHO does not recommend the use of NAT techniques within HIV testing algorithms for individuals ≥18 months of age.
- The first test in an HIV testing strategy and algorithm should have the highest sensitivity, followed by a second and third test of the highest specificity. Algorithms should be validated and verified to ensure high quality and accurate testing services.
- All people newly diagnosed with HIV should be retested to verify their HIV status prior to starting ART, using the same testing strategy and algorithm as the initial test. To minimize the risk of misdiagnosis, this approach should be maintained in settings in which rapid ART initiation is being implemented.

Source: WHO 2019, [6]

To support the implementation of the 2019 guidelines, WHO launched and disseminated the guidance at the Africa Society of Laboratory Medicine and the International Conference on AIDS and STIs in Africa. Following the initial release, WHO also provided detailed country support to adopt the guidelines along with developing an application to access the guidelines

For peer review only - http://bmjopen.bmj.com/site/about/guidelines.xhtml

more easily. In addition to dissemination and country support, [10] it is critical to monitor and track the implementation of WHO policy uptake to understand policy priorities and challenges overtime. Such tracking not only provides valuable insights but can help guide revisions to future guidance and can help target country policy support [11—12].

A global review of national HIV testing policies was conducted by WHO in 2018 to assess adoption of its testing recommendations and policies.[13] Of 91 policies reviewed only 24 (25%) adhered to WHO guidance. This policy review follows on from the previous review and seeks to assess country uptake of six 2019 WHO recommendations related to HIV testing strategies. Here we focus on the WHO African region, which hosts several countries with a high HIV burden and with large HIV testing programmes. The same policy review across the remaining five WHO regions is underway as part of the update to the 2023 WHO HTS guidelines.

METHODS

Search strategy

We carried out a comprehensive desk review of national HIV testing policies in 47 Member States in the WHO African region (online supplementary figure 1) using a policy repository maintained by WHO, scanning government websites, and by contacting government officials or other relevant informants through December 2021. The most current policy documents containing information on the HTS policy were included, but when this was unavailable, we included the most recent HIV testing algorithm, but if that was also unavailable, we opted to include previous national policies. Documents of all languages were included. Other supporting documents related to algorithm validation and PMTCT guidelines were identified,

when possible, either through references provided in the national HTS policy or through contact with key informants. The full protocol for the review was previously developed and published in 2018. [13]

Data extraction

Data were extracted from each policy document by one reviewer (EF) into standardised coding forms on policy information, HIV testing strategy/algorithm (>18 months of age), dual HIV/syphilis testing strategy/algorithm, and retesting prior to ART initiation (online supplementary table 1). When reported the order, type of test kits and name brand of diagnostics used within the HIV testing algorithm was summarised descriptively, as well as information on whether the algorithm was verified or validated.

A second reviewer (CL) carried out crosschecking of the data. Differences between coders were resolved through a third reviewer (CJ). To prevent misclassification, items were marked as ‘unclear’ during data extraction when lack of information prevented complete understanding. Reviewers than worked to contact key informant to provide further detail and clarity wherever possible.

Analysis

We assessed national adoption of WHO HTS guidance using six specific recommendations related to testing strategies and algorithms for those ≥18 months of age set forth in the WHO 2019 HIV testing guidelines, namely: (1) use of serial testing, (2) use of a 3-test strategy, (3) discontinuation of a tie-breaker to rule in HIV infection, (4) discontinuation of WB/LIA, (5) retesting prior to ART initiation, and (6) use of dual HIV/syphilis RDT in ANC. Additional

For peer review only - http://bmjopen.bmj.com/site/about/guidelines.xhtml

qualitative details about the national testing algorithm, such as the order of and name brand of test kits used in testing algorithms, were also reviewed when reported and assessed according to WHO recommendations (see Box 1).

Based on the number of recommendations adopted, national policies were categorized as: adopted (6); nearly adopted (5 to 4); somewhat not adopted: (3); and not adopted: (2 or less). We then provide simplified reporting (adopted, partially adopted and not adopted) for national policies which reviewed in 2018 and 2021 to assess changes overtime. Notably, these categories were initially developed as part of the 2018 policy review and maintained to assist with future updates and policy tracking.

Descriptive analyses were then stratified by subregions (Western, Central, Eastern and Southern Africa; online supplementary figure 1) were also conducted to determine rates of adherence by subregion. All analyses were conducted in Microsoft Excel. Countries which had policies reviewed in 2018 and 2021 were also compared to assess changes in alignment with WHO recommendations over time.

Patient and public involvement

Patients and/or the public were not involved in the design, conduct, reporting, or dissemination plans of this study.

RESULTS

In total, we were able to identify policy documents from 96% of countries from the WHO African region (n=45/47); 71% of countries had a policy reviewed in both 2018 and 2021

(n=32/45). Two countries, Republic of the Congo and Cabo Verde, were not included because we were unable to obtain their national policies and sufficient information (figure 1). Out of all these policies, 91% (41/45) had updated their national policies as of December 2021. Policy publication dates ranged from 2013 to 2021. Out of the six country policies that had not been fully updated since 2018 (Algeria, Botswana, eSwatini, Cote d'Ivoire, Malawi, and Zimbabwe), two (Malawi and Zimbabwe) provided new HIV testing algorithms that were included in the review.

Of the 45 policies providing information on HIV testing strategies, 16 were from Western Africa (36%), 16 were from Eastern Africa (36%), 8 were from Central Africa (18%), and 5 were from Southern Africa (11%). Policies were published in English (n=22), French (n=18), Portuguese (n=4) and Spanish (n=1). Based on the most recent national HIV prevalence reported by UNAIDS [1], 34 countries (76%) had a low HIV prevalence (<5%) and 11 countries (24%) had a high HIV prevalence (≥5%) (online supplementary table 2).

Overall adoption of WHO-recommended HIV testing strategies

In 2021, 62% of national testing strategies (n=28/45) were either fully or partially aligned with WHO 2019 HTS recommendations and 38% (n=17/45) had not adopted the recommendations (table 1). When the analysis was restricted to a subset of countries with policies in both the 2018 and 2021 review (n=32), the proportion adopting WHO guidance in 2018 was 28% (n=9/32) *vs* 62% (n=20/32) in the current (2021) review (see online supplementary table 3; supplementary figure 2). Across the sub-regions, Southern (80%) and Eastern (75%) Africa had the greatest level of alignment, whilst Central Africa (63%) and Western Africa (44%) had the lowest adoption rates (table 1; figure 2).

For peer review only - http://bmjopen.bmj.com/site/about/guidelines.xhtml

In low-prevalence countries (n=34), 53% of policies (n=18/34) had fully or mostly adopted the guidance and 47% (n=16/34) had generally not adopted guidance. The most common reasons for non-adoption were not using a dual HIV/syphilis RDT in ANC (n=15/34), no retesting prior to ART initiation (n=14/34) and using only two assays to make an HIV-positive diagnosis (n=10/34). In high-prevalence countries (n=11), 91% of policies (n=10/11) had fully or nearly adopted WHO guidance and 9% had not adopted WHO guidance (1/11). The most common reasons for not fully adopting WHO guidance was: continuing to use a 2-test strategy to make an HIV-positive diagnosis (n=10/11), employing both serial and parallel testing strategies (n=4/11), not adopting dual HIV/syphilis RDTs in ANC (n=4/11) and not implementing retesting prior to ART initiation (n=3/11). Overall, only two out of eleven high HIV-burden countries, Malawi and Zimbabwe, recommended the use of a 3-assay testing strategy (18%).

For peer review only - http://bmjopen.bmj.com/site/about/guidelines.xhtml

Table 1. Policy adoption of 2019 WHO-recommended HIV testing strategies in the WHO African region, 2021

AFRO subregions (countries)	Policies reviewed	Serial testing strategy	Discontinuation of tiebreaker* testing strategy	Retesting prior to ART initiation	Recommended 3-test strategy	Use of dual HIV/syphilis test**	No use of Western Blot
**All (n=47)**	45 (96%)	29 (64%)	37 (82%)	23 (51%)	21 (47%)	21 (47%)	35 (78%)
**Western (n=17)**	16 (94%)	9 (56%)	9 (56%)	5 (31%)	8 (50%)	7 (44%)	10 (63%)
**Central (n=9)**	8 (89%)	7 (88%)	7 (88%)	5 (63%)	3 (38%)	2 (25%)	6 (75%)
**Eastern (n=16)**	16 (100%)	12 (75%)	16 (100%)	9 (56%)	9 (56%)	9 (56%)	14 (88%)
**Southern (n=5)**	5 (100%)	1 (20%)	5 (100%)	4 (80%)	1 (20%)	3 (60%)	5 (100%)

*Use of a third assay to rule-in HIV infection

**Dual HIV/syphilis RDT as first test in ANC

Adoption of serial testing strategy

In 2021, 64% of national testing strategies (n=29/45) adopted the use of serial testing and 36% (n=16/45) recommended the mix use of serial and parallel testing (figure 3_1). The subregions with the highest adoption were Central (88%) and Eastern Africa (75%), followed by Western Africa (58%); Southern Africa had the lowest adoption rate (20%) (table 1). However, fewer countries in southern Africa recommended serial testing in 2021 (n=1) than in 2018 (n=4) (supplementary table 4). Most policies recommending serial or parallel testing (n=15/16) conducted simultaneous testing of assay 1 (A1) and assay 2 (A2) in case of discrepant test results, and the Namibian policy recommended parallel testing of A2 and A3 after a reactive A1.

Only 44% of policies (n=20/45) provided guidance on the assay order in relation to their sensitivity and specificity. The proportion of HIV testing strategies/algorithms using two consecutive HIV-reactive tests to make an HIV-positive diagnosis was 53% (n=24/45) whereas 47% (n=21/45) recommended the use 3 consecutive HIV-reactive tests.

Sixty-four percent of the HIV testing strategies reviewed (n=29/45) included information on the testing algorithms, i.e., contained specific product names and all of them included WHO prequalified products. Determine HIV 1/2 (Abbott Diagnostics Medical Co., Ltd, Japan) was the most common product used as A1 and Bioline HIV 1/2 3.0 (Abbott Diagnostics Korea Inc., Republic of Korea) or Uni-Gold HIV (Trinity Biotech, Ireland) as A2 or A3 (see online supplementary table 5). Only 31% of policies (n=14/45) mentioned the need to locally verify or validate the HIV testing algorithm.

Discontinuation of a tiebreaker testing strategy

The discontinuation of a third assay to rule in HIV infection (i.e., tiebreaker) was recommended in 82% of national policies (n=37/45) (figure 3_2). The subregions with the highest adoption were in Southern Africa and Eastern Africa (100%, each) followed by Central Africa (88%), whereas Western Africa had the lowest adoption (56%) (table 1).

Of the eight policies recommending a tiebreaker testing strategy, seven were from Western Africa and one from Central Africa. Seven policies specified the type of A3 to be used, for example, RDT (n=4), RDT or EIA (n=1), nucleic acid testing (NAT: n=2) and LIA (n=1).

Adoption of WHO standard 3-assay testing strategy

The use of three consecutive HIV-reactive tests for the diagnosis of HIV was recommended by 47% of the policies (n=21/45) (figure 3_3). The adoption of this WHO recommendation was highest in Eastern Africa (56%) and Western Africa (50%) and lowest in Southern Africa (20%) (table 1). Most policies recommending a 3-test strategy (n=20/21) were from low HIV-burden countries. Among high HIV-burden countries, only 18% of policies (n=2/11) recommended the use of a 3-test strategy and this corresponded to two countries, Malawi and Zimbabwe, respectively.

Overall, across countries which had policies reviewed in both 2018 and 2021, 10 policies that previously recommended a 2-assay testing strategy transitioned to a 3-test strategy as of 2021 (Burkina Faso, Burundi, Chad, Ethiopia, Ghana, Kenya, Lesotho, Malawi, Madagascar, and Zimbabwe, respectively).

For peer review only - http://bmjopen.bmj.com/site/about/guidelines.xhtml

Adoption of retesting to verify positive status prior to ART initiation

In 2021, 51% of national strategies (n=23/45) recommended retesting prior to ART initiation (figure 3_4). The regions with the highest level of policy adoption were in Southern Africa (80%) followed by Central (63%) and Eastern Africa (56%) (table 1). However, fewer countries in southern Africa recommended retesting before ART initiation in 2021 (n=4) than in 2018 (n=5) (supplementary table 5). Sixty-five percent of the retesting policies (n=15/23) recommended retesting using the same national HIV testing algorithm in a new specimen run by a different operator in a different testing site; 26% (n=6/23) did not specify how retesting was carried out and 9% of policies (n=2/23) utilized retesting strategies that differed from the national HIV testing algorithm, which included parallel testing of A1/A2 in Botswana and Malawi.

Discontinuation of Western Blotting

The proportion of national policies not including WB or LIA as part of the HIV testing algorithm was 78% (n=35/45) (figure 3_5). This proportion was highest in Southern Africa (100%) and Eastern Africa (88%) followed by Central Africa (75%) and Western Africa (63%) (table 1). Most countries with a supporting policy in favour of WB or LIA (n=9/10) recommended its use to manage persistent inconclusive/indeterminate test results and one policy used WB as A3 (Mauritius).

Adoption of dual HIV/syphilis RDT in ANC settings

Forty-seven percent (n=21/45) of policies supported the use of dual HIV/syphilis RDTs among pregnant women in ANC (figure 3_6). The region was the highest policy uptake was Southern Africa (60%), followed by Eastern (56%), Western (44%) and Central Africa (25%) (table 1).

And of policies supporting a 3-test strategy, only 53% (n=11/21) also recommended the use of dual HIV/syphilis RDTs among pregnant women. The majority of policies (n=20/21) recommended the use of dual HIV/syphilis RDT as A1 among pregnant women and only one policy (Lesotho) recommended its use as a triage test (A0) as an interim algorithm while waiting for the results of a national verification study.

The dual HIV/syphilis testing algorithm was not aligned with the national HIV testing algorithm in one country (Zambia): while Determine HIV-1/2 (Abbott Diagnostics Medical Co., Ltd, Japan) and SD Bioline HIV-1/2 3.0 (Abbott Diagnostics Korea Inc, Republic of Korea) products were used as A1 and A2 in the national testing algorithm, Bioline HIV/Syphilis Duo (Abbott Diagnostics Korea Inc, Republic of Korea) and Rapid Test for Antibody to Human Immunodeficiency Virus (HIV) (Colloidal Gold Device) (Beijing Wantai Biological Pharmacy Enterprise Co., Ltd., China) were used as A1 and A2 in the ANC testing algorithm.

In addition to the use in ANC settings, three countries also recommended testing with dual HIV/syphilis RDTs among other populations such as male partners of pregnant women and key populations (Liberia, Madagascar and Uganda). Only 50% of policies (n=11/21) specified the brand name of the dual HIV/syphilis RDT in the testing algorithm, and among the policies mentioning the brand name, Bioline HIV/Syphilis Duo (Abbott Diagnostics Korea Inc, Republic of Korea) was the most employed RDT (100%). Only 57% of policies (n=12/21) mentioned whether syphilis treatment was provided immediately after a reactive syphilis test result.

Use of nucleic acid testing (NAT) to diagnose HIV infection in individuals ≥18 months of age

Twenty-two percent of national policies (n=10) recommended the use of NAT as part of the testing algorithm for the diagnosis of HIV infection in individuals ≥18 months of age. Four policies were from Eastern Africa (Malawi, Mauritius, Mozambique and United Republic of Tanzania); three national policies from Southern Africa (Eswatini, Lesotho and Namibia); two policies from Central Africa (Angola and Sao Tomé et Principe); and one national policy from Western Africa (Guinea Bissau). Two policies employed NAT as the third assay (A3) of the algorithm while the remaining 8 policies recommended NAT to resolve persistent inconclusive/indeterminate test results (referring either to viral load, PCR or DNA-PCR).

DISCUSSION

The current review found a significant improvement in the overall policy uptake of the latest WHO-recommended HIV testing strategies with 62% of countries in the African region adopting WHO guidance in 2021. When directly comparing 32 countries with policies reviewed in 2018 and 2021, [13] policy adoption increased more than 2-fold (20 vs 9). As of the 2021 review, 40% of policies were published before 2019 which underscores the need to update national testing policies at more regular intervals.

Given the rapid changes in HIV testing landscape, this underscores the need of national programmes to update their national HIV testing policies at more regular intervals to keep up with latest WHO guidance. This is particularly critical now as WHO is now updating HTS guidance in 2023 and it will be essential for WHO to continue policy tracking, [10] to allocate sufficient resources, to conduct implementation science research to understand bottlenecks

355 hinder policy change and to support country-led technical working groups to drive policy
356 change [11—12].

357

358 The recommendations with highest adoption were the discontinuation of a tiebreaker (82%)
359 and western blotting (78%) as well as the use of serial testing (64%). Half of countries (51%)
360 also recommend retesting prior to ART initiation. One likely explanation for the higher policy
361 adoption is that these WHO´s recommendations were first published in 2012, 2014 and 2015,
362 respectively (save for discontinuation of western blotting) providing national HIV
363 programmes ample time to incorporate these recommendations into their national HIV
364 testing guidelines. Although moving away from western blotting was recommended more
365 recently in 2019,[14] low resource countries in the WHO African region have been at the
366 forefront of implementing HIV RDTs compared to other WHO regions as a way to rapidly
367 expand and increase access to HIV testing services.[15]

368

369 Having said that, our review found that a dozen of countries, mostly in Western Africa, still
370 use western blotting as part of the HIV testing algorithms, hindering same-day diagnoses and
371 rapid access to ART and pre-exposure prophylaxis (PrEP). Additionally, between 2018 and
372 2021, countries in southern Africa appeared to be the one sub-region which shifted away
373 from serial testing to parallel testing strategies. This shift has likely increased testing costs as
374 it requires more test kits per individual tested. Further follow-up with countries is needed to
375 understand their policies and should continue to promote serial testing.

376

377 Despite observing that many countries recommended retesting prior to ART initiation in 2021,
378 we noted that progress in policy adoption was minimal and that in southern Africa one less

country was implementing when compared to 2018. Retesting prior to ART initiation remains an important quality assurance strategy, however if countries are struggling to adopt this recommendation it may be important for WHO to provide alternative approaches and address implementation challenges.

The WHO´s recommendations with a lower country uptake were the use of a standard 3-test strategy (47%) and the use of dual HIV/syphilis RDT among pregnant women (47%), both recommendations released in 2019.[6] Before 2019, WHO recommended the use of a 2-test strategy in high HIV prevalence settings (≥5%) and the use of a 3-test strategy in low HIV prevalence settings (<5%) to maintain at least a 99% positive predictive value.[1] However, as HTS and ART have been scaled up substantially and fewer people undergoing HTS are HIV-positive, the national HTS positivity has also declined, even in high HIV burden settings.[16] Given these changes in the epidemic, WHO now recommends that all settings move toward using a 3-assay testing strategy to ensure high-quality testing.[16] Specifically, in high HIV burden settings in southern Africa (5 countries) and eastern Africa (6 countries), WHO recommends countries still using a 2-test strategy to prioritize moving toward using a 3-test strategy.[16]

In this review we found that only 18% (n=2/11) of the high-burden countries in the WHO African region has a supportive policy on a 3-assay testing strategy. A likely explanation for this slower policy adoption is programmes needing to prioritise limited resources and efforts to address the COVID-19 pandemic. Many programmes, including those focused on HIV testing, were affected by disruptions during COVID-19 and updates and changes to national policies were delayed. [4] Changing a testing algorithm also takes time and planning to

403 implement, including provider training, quality assurance, procurement, inventory
404 management and budget implications to the national programme.[6,16] Many countries
405 indicated they were in the process of transitioning to a 3-test strategy but needed to complete
406 verification studies, conduct tendering process for products, and garner consensus and
407 support among partners. Some countries have yet to prioritise adopting the 3-test strategy
408 because they were focused on higher yield testing approaches alone. [17—18] Lastly, some
409 countries had delayed the transition due to costs concerns. While countries will need to
410 develop a plan and identify the optimal time for the transition, in terms of costs, a modelling
411 study found that the total cost of the 3-test strategy would have a very limited impact on
412 costs.[19] Further, in Ghana after transitioning to a 3-test strategy they found that while there
413 were lessons learned about logistics and training, the new strategy was found to be feasible,
414 improved testing quality and reduced costs due to preventing misdiagnosis.[20]

415

416 We found that among low-prevalence countries (n=34), a significant proportion (41%) still use
417 a suboptimal testing strategy with 2 assays to make an HIV positive diagnosis, which will
418 increase the likelihood of false-positive diagnosis.[19--21] A retrospective study in Nigeria
419 using household survey data found that the performance of the 2-test strategy in a low
420 prevalence setting of about 1.4% was poor with a PPV of 94% and a false-positive rate of
421 5.5%.[22] This positive predictive value is similar to a modelling study assessing the accuracy
422 of the 2015 and 2019 WHO HIV testing algorithms, which was 95.4% using a 2-assay testing
423 strategy.[19]

424

425 Considering that the recommendation to use dual HIV/syphilis RDTs in ANC was released only
426 in 2019,[6, 23] its adoption has been rapid with 21 national policies recommending its use.

For peer review only - http://bmjopen.bmj.com/site/about/guidelines.xhtml

However, the number of countries adopting dual HIV/syphilis RDTs among pregnant women may be in practice higher considering that preliminary National Commitments and Policy Instrument (NCPI) survey data reported that a total of 26 countries in the WHO African region adopted the use of dual HIV/syphilis RDTs in ANC. [24] The differences likely reflect differences in methodologies (policy reviews versus policy survey). Often annual global policy surveys, like NCPI, may reflect a mixture of policies and anecdotal practices, including unofficial policies not yet within official guidelines. Although the findings of this review likely provide a conservative estimate of policy adoption, they do highlight the need to ensure official national policies are updated accordingly.

Some countries may choose not to introduce dual HIV/syphilis RDT. For example, in Malawi where dual HIV/syphilis RDT introduction has been considered but not adopted, the national programme has opted to retain stand-alone HIV and syphilis RDTs in ANC to prioritize point-of-care test options for HIV and syphilis while maintaining a single HIV testing algorithm in a resource limited context. Dual HIV/syphilis testing is also now recommended for key populations by WHO and this may further increase its use in HIV testing algorithms in national programmes. [25] It is an important option to increase HIV and syphilis detection and treatment and a recent modelling study in Viet Nam shows that it was cost saving or cost-effective.[26]

As countries update their national HIV testing guidelines to incorporate latest WHO guidance and adapt their national HIV testing strategies and algorithms to shift toward using three consecutive reactive serology tests to diagnose HIV, introduce dual HIV/syphilis RDTs among pregnant women and key populations, move away from western blotting, reinforcing

retesting prior to ART initiation as well as design flexible algorithms to address kit shortages, WHO encourages national programmes to conduct a verification study to provide objective evidence, before national scale-up, that a specific combination of products work well together without sharing false-reactivity, thus reducing the risk of misdiagnosis.[6, 9] To assist countries achieve this, WHO has put together a practical toolkit to accelerate policy adoption.[27] To date, the following countries are working directly with WHO to update their testing algorithm: Armenia, Burkina Faso, Cameroon, Chad, Central African Republic, Cote D´Ivoire, Democratic Republic of Congo, eSwatini, Kenya, Lao, Lesotho, Mali, South Sudan and Zambia.

This policy review has strengths as well as weaknesses. Firstly, this review provides a comprehensive review of existing national HTS policies in the African region and was able to identify current policies for 45 countries. While this represents nearly all countries in the region, we were unable to identify policies for two countries. Secondly, unlike other policy surveys this study reviewed official policies for each country that were collected through a robust global search and triangulated data with additional information provided by country-level key informants. Despite this, the review was unable to assess unpublished policies and those in development at the time of the analysis. During the review some countries indicated that they were in the process of updating their guidance, particularly around adoption of a 3-test strategy. We were unable to include this information in the review, and thus our reported level of adoption of WHO recommendations may be an underestimate. Thirdly, this review did not assess policy implementation at the national, subnational, or site level. Thus, it is possible that some practices in country differ than policies reviewed, and level of adoption reported may vary. Lastly, we focused on HIV testing strategies for individuals ≥18 months of

For peer review only - http://bmjopen.bmj.com/site/about/guidelines.xhtml

age. As a result, we were unable to report on the adoption of HIV testing strategy recommendations for children, such as early infant diagnosis.

Conclusions

Adoption of WHO-recommended HIV testing strategies has improved in the African region. While WB was only used in a few countries, concerted efforts are needed to phase out this technology in favour of RDTs. Countries should plan to accelerate their transition to WHO recommendations by streamlining efforts to adopt and implement a 3-asssay testing strategy and dual HIV/syphilis RDTs. Conducting verification of testing algorithms and using appropriate assays can ensure accurate HIV diagnosis in a cost-efficient and time-efficient manner. Greater efforts are needed now to ensure countries implement high-quality testing services as they are essential for the global goal to achieve and maintain low HIV incidence.

Acknowledgements

We would like to express our gratitude to WHO colleagues in the respective countries of the WHO African region for their support in locating updated national policy documents. We would also like to thank staff at the Global Fund (Youssouf Sawadogo, Ghislaine Grasser and Jacqueline Papo), CHAI (Katherine Guerra, Gillian Leitch, Megan Ginivan and Christian Stillson), Evidence Action Group (Anna Konstantinova and Emilie Efroson) and staff from Ministry of Health in Mozambique (Helga Guambe) and Guinea Bissau (David da Silva Té) for their support in identifying latest national HIV testing policies.

Contributors

CJ and AS devised and CJ and RB supervised the review. EF led the study protocol developments, conducted the screening, data extraction and analysis. NB, CM, NSB, FSM, FJL, MASP, LH assisted with acquisition of policies, data collection and results interpretation. CL acted as a second reviewer and CJ as third reviewer. AS, MSJ, MBD and RB assisted with assessing policies and interpreting results. EF led manuscript writing along with CJ, with support from all authors. CL, NB, CM, NSB, FSM, FJL, MASP, LH, MBD, MSJ, AS, RB all reviewed the first draft, provided critical review and input and approved the final version of the manuscript.

Funding statement

This work was supported by the Bill and Melinda Gates Foundation, grant number INV-024432. The funders had no role in study design, data collection and analysis, decision to publish, or preparation of the manuscript. Authors declare no competing interests. The views expressed in this manuscript are those of the authors and do not necessarily represent the official position of the WHO.

Map disclaimer

The depiction of boundaries on the map(s) in this article do not imply the expression of any opinion whatsoever on the part of BMJ (or any member of its group) concerning the legal status of any country, territory, jurisdiction or area or of its authorities. The map(s) are provided without any warranty of any kind, either express or implied.

For peer review only - http://bmjopen.bmj.com/site/about/guidelines.xhtml

Competing interests

The authors declare no competing interests. The contents in this article are those of the authors and do not necessarily reflect the view of the World Health Organization.

Patient consent for publication

Not required.

Ethics approval

This study does not involve human participants and ethical approval was therefore not required.

Data availability statement

All data generated in the study are included in the article or uploaded as supplementary information. National policies may be publicly available. Some policies included in this review may be available through the following websites: (1) https://aidsfree.usaid.gov/resources/guidance-data/hts. (2) http://www.hivpolicywatch.org/database.html. If information on a policy cannot be found through these resources, please contact the authors of this review for additional information.

For peer review only - http://bmjopen.bmj.com/site/about/guidelines.xhtml

REFERENCES

1. Consolidated guidelines on HIV testing services, 2015. Geneva: World Health Organization; 2015. Available from: https://www.who.int/publications/i/item/9789241508926 [cited 2022 Feb 24].

2. Guidelines on when to start antiretroviral therapy and on pre-exposure prophylaxis for HIV, 2015. Geneva: World Health Organization; 2015. Available from: https://www.who.int/publications/i/item/9789241509565 [cited 2022 Feb 24].

3. Frescura L, Godfrey-Faussett P, Feizzadeh A A, El-Sadr W, Syarif O, Ghys PD; on and behalf of the 2025 testing treatment target Working Group. Achieving the 95 95 95 targets for all: A pathway to ending AIDS. PLoS One. 2022 Aug 4;17(8):e0272405.

4. Assessment of HIV testing services and antiretroviral therapy service disruptions in the context of COVID-19: lessons learned and way forward in sub-Saharan Africa. World Health Organization; 2021. Available from: https://www.who.int/publications/i/item/9789240039599 [cited 2022 Feb 24].

5. Fact sheet. Latest global and regional statistics on the status of the AIDS epidemic [internet]. Geneva: United Nations Program on HIV/AIDS (UNAIDS); 2021. Available from: https://www.unaids.org/sites/default/files/media_asset/UNAIDS_FactSheet_en.pdf [cited 2022 Feb 24].

6. Consolidated guidelines on HIV testing services, 2019. Geneva: World Health Organization; 2019. Available from: https://www.who.int/publications/i/item/978-92-4-155058-1 [cited 2022 Feb 24].

7. Service delivery approaches to HIV testing and counselling (HTC): a strategic HTC programme framework, 2012. Geneva: World Health Organization; 2012. Available from: https://apps.who.int/iris/handle/10665/75206 [cited 2022 Oct 10].

8. Tippett Barr BA, Lowrance D, Johnson CC, Baggaley RC, Rogers JH, Balachandra SK, Barker J, Kalua T, Bunga S, Low-Beer D, Payne D, Bulterys MG, Jahn A. Treatment-adjusted prevalence to assess HIV testing programmes. Bull World Health Organ. 2021 Dec 1;99(12):874-882.

9. Optimizing HIV testing algorithms: a generic verification protocol for selecting appropriate HIV serology assays and assessing the level of shared false-reactivity. Geneva: World Health Organization; 2021. Available from: https://www.who.int/publications/i/item/9789240039162 [cited 2022 Feb 24].

10. Flodgren G, Hall AM, Goulding L, Eccles MP, Grimshaw JM, Leng GC, Shepperd S. Tools developed and disseminated by guideline producers to promote the uptake of their guidelines. Cochrane Database Syst Rev. 2016 Aug 22;(8):CD010669. doi: 10.1002/14651858.CD010669.pub2.

11. Nilsen P, Ståhl C, Roback K, Cairney P. Never the twain shall meet?--a comparison of implementation science and policy implementation research. Implement Sci. 2013 Jun 10;8:63. doi: 10.1186/1748-5908-8-63..

12. Oh A, Abazeed A, Chambers DA. Policy Implementation Science to Advance Population Health: The Potential for Learning Health Policy Systems. Front Public Health. 2021 Jun 17;9:681602. doi: 10.3389/fpubh.2021.681602.

13. Fonner VA, Sands A, Figueroa C, Baggaley R, Quinn C, Jamil MS, Johnson C. Country adherence to WHO recommendations to improve the quality of HIV diagnosis: a global policy review. BMJ Glob Health. 2020 May;5(5):e001939.

14. WHO recommends countries move away from the use of western blotting and line immunoassays in HIV testing strategies and algorithms. Geneva: World Health Organization; 2019. Available from: https://www.who.int/publications/i/item/WHO-CDS-HIV-19.30 [cited 2022 Oct 10].

15. Flynn DE, Johnson C, Sands A, Wong V, Figueroa C, Baggaley R. Can trained lay providers perform HIV testing services? A review of national HIV testing policies. BMC Res Notes. 2017 Jan 4;10(1):20. doi: 10.1186/s13104-016-2339-1. PMID: 28057054; PMCID: PMC5216526.

16. WHO encourages countries to adapt HIV testing strategies in response to changing epidemic. Geneva: World Health Organization; 2019. Available from: https://www.who.int/publications/i/item/WHO-CDS-HIV-19.34 [cited 2022 Oct 10].

17. Jubilee M, Park FJ, Chipango K, Pule K, Machinda A, Taruberekera N. HIV index testing to improve HIV positivity rate and linkage to care and treatment of sexual partners, adolescents and children of PLHIV in Lesotho. PLoS One. 2019 Mar 27;14(3):e0212762.

18. Ahmed S, Schwarz M, Flick RJ, Rees CA, Harawa M, Simon K, Robison JA, Kazembe PN, Kim MH. Lost opportunities to identify and treat HIV-positive patients: results from a baseline assessment of provider-initiated HIV testing and counselling (PITC) in Malawi. Trop Med Int Health. 2016 Apr;21(4):479-85.

19. Eaton JW, Sands A, Barr-DiChiara M, Jamil MSJ, Baggaley R, Tippett Barr BA, Kalua T, Jahn A, Maheu-Giroux, Johnson. Accuracy and performance of the WHO 2015 and WHO 2019 HIV testing strategies across epidemic settings. medRxiv 2021.03.31.21254700. Available from: https://www.medrxiv.org/content/10.1101/2021.03.31.21254700v1. [cited 2022 Oct 10].

20. Rowland Adukpo. Experience from Ghana in transitioning to a 3-assay HIV testing strategy. WHO satellite session “Improving quality of HIV diagnosis: do you know how to update your HIV national testing algorithms? At the African Society of Laboratory Medicine Conference, 18 Nov 2021. Available from: https://www.who.int/news-room/events/detail/2021/11/18/default-calendar/improving-quality-of-hiv-diagnosis-toolkit-launch [cited 2022 Oct 10].

21. Johnson CC, Fonner V, Sands A, Ford N, Obermeyer CM, Tsui S, Wong V, Baggaley R. To err is human, to correct is public health: a systematic review examining poor quality testing and misdiagnosis of HIV status. J Int AIDS Soc. 2017 Aug 29;20(Suppl 6):21755.

22. Patel HK, Ikpe S, Bronson M, Birhanu S, Abimiku A, Jahun I, Detorio M, Lupoli K, Yavo D, Bassey OO, Jelpe TD, Kagurusi B, Iriemenam NC, et al. (2022) Performance of HIV rapid testing algorithm in Nigeria: Findings from a household-based Nigeria HIV/AIDS Indicator and Impact Survey (NAIIS). PLOS Glob Public Health 2022 2(7): e0000466.

23. Dual HIV/syphilis rapid diagnostic tests can be used as the first test in antenatal care. Geneva: World Health Organization; 2019. Available from: https://www.who.int/publications/i/item/WHO-CDS-HIV-19.38. [cited 2022 Oct 10].

24. UNAIDS Joint United Nations Programme on HIV/AIDS and WHO. Laws and policies analytics. Available from: https://lawsandpolicies.unaids.org/topicresult?i=13&o=219,397&lan=en. [cited 2022 Oct 10].

25. Consolidated guidelines on HIV, viral hepatitis and STI prevention, diagnosis, treatment and care for key populations. Geneva: World Health Organization; 2022. Available from: https://www.who.int/publications/i/item/9789240052390. [cited 2022 Oct 10].

26. Coomes D, Green D, Barnabas R, Sharma M, Barr-DiChiara M, Jamil MS, Baggaley R, Owiredu MN, Macdonald V, Nguyen VTT, Vo SH, Taylor M, Wi T, Johnson C, Drake AL. Cost-effectiveness of implementing HIV and HIV/syphilis dual testing among key populations in Viet Nam: a modelling analysis. BMJ Open. 2022 Aug 11;12(8):e056887.

27. Toolkit to optimize HIV testing algorithms. Geneva: World Health Organization; 2021. Available from: https://www.who.int/tools/optimizing-hiv-testing-algorithms-toolkit. [cited 2022 Oct 11].

For peer review only - http://bmjopen.bmj.com/site/about/guidelines.xhtml

Figure legends

Figure 1. Countries from the WHO African Region with HIV testing policies identified (in blue) and included in the analysis (n=45/47). The two countries not included in the review are indicated in the map.

Figure 2. Overall adoption of 2019 WHO HIV testing strategies in Africa by subregion in 2021.

Figure 3. Policy adoption in 2021 of six specific WHO HIV testing recommendations in the WHO African Region.

For peer review only - http://bmjopen.bmj.com/site/about/guidelines.xhtml

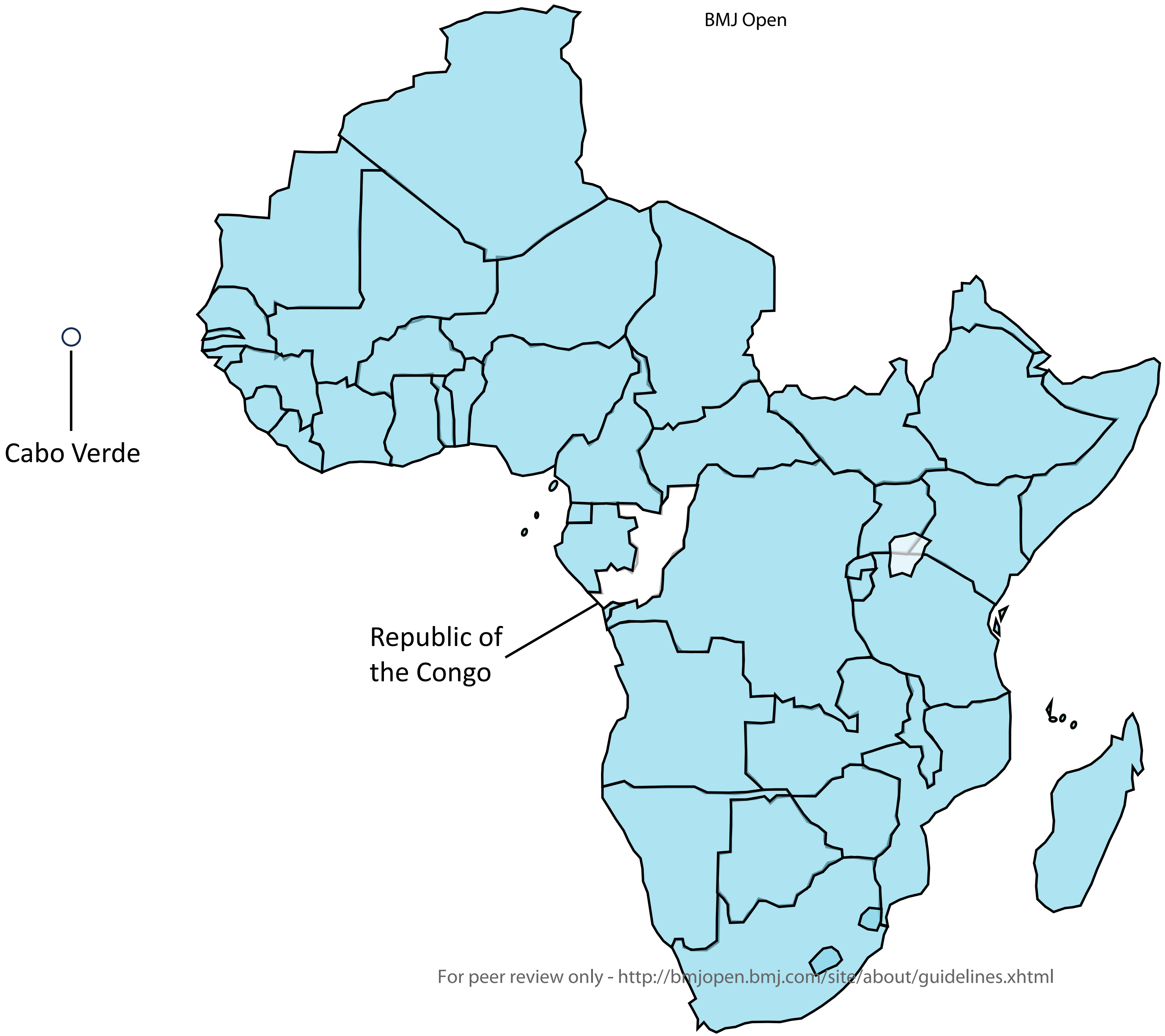
Cabo Verde
Republic of
the Congo

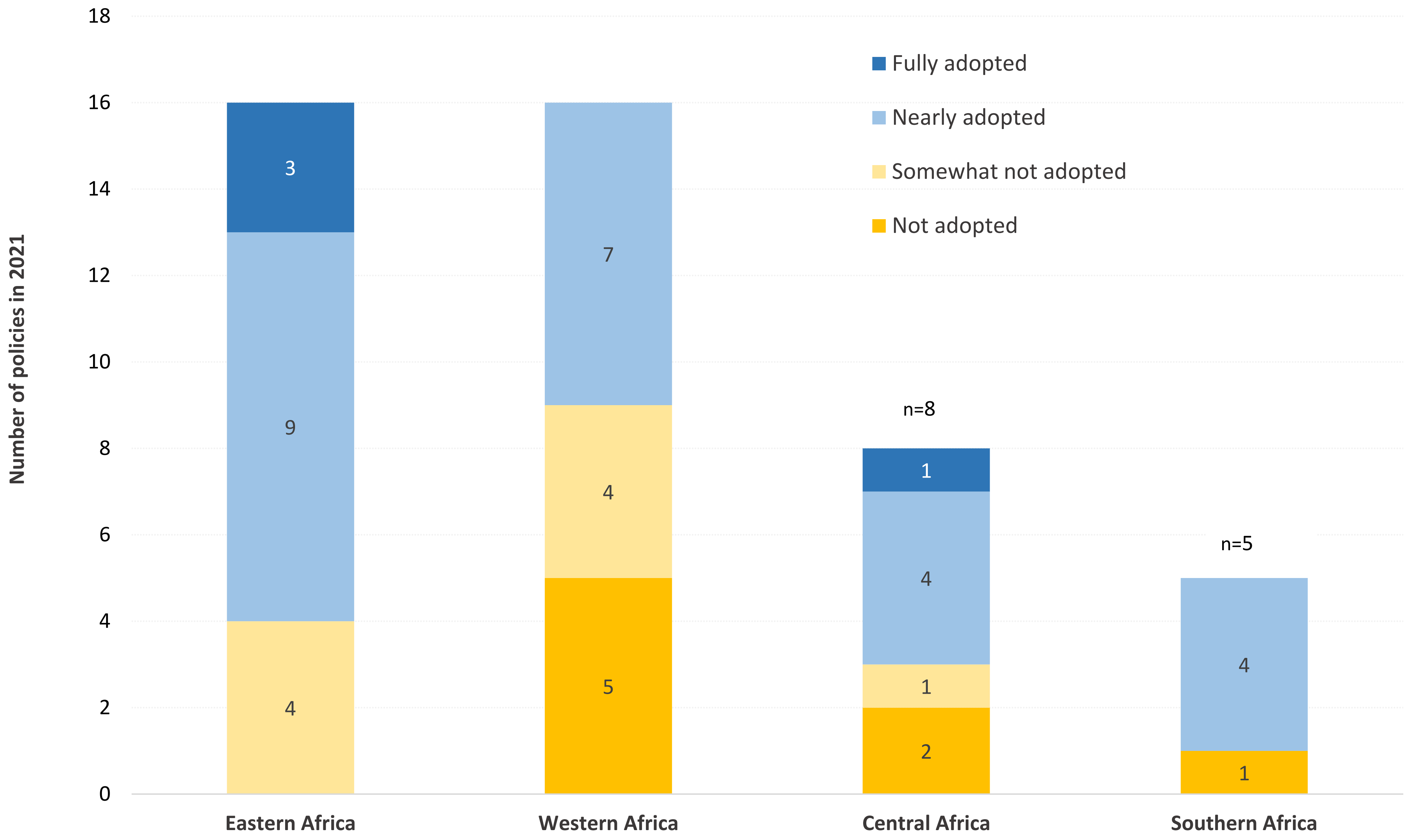
18
16
14
12
10
8
6
4
2
0
Number of policies in 2021
Fully adopted
Nearly adopted
Somewhat not adopted
Not adopted
3
9
4
7
4
5
n=8
1
4
1
2
n=5
4
1
Eastern Africa
Western Africa
Central Africa
Southern Africa

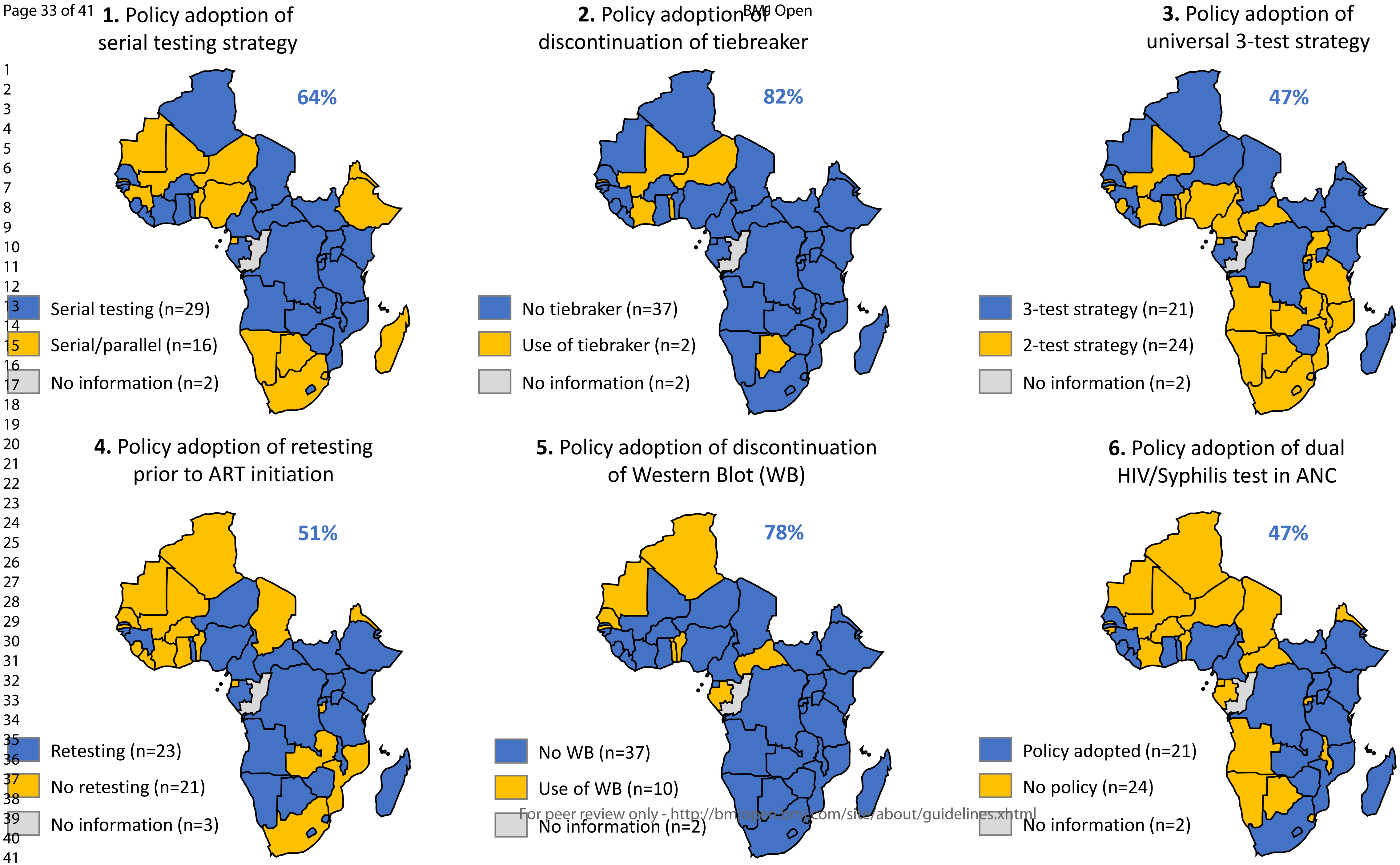
1. Policy adoption of serial testing strategy
64%
Serial testing (n=29)
Serial/parallel (n=16)
No information (n=2)
2. Policy adoption of discontinuation of tiebreaker
82%
No tiebraker (n=37)
Use of tiebraker (n=2)
No information (n=2)
3. Policy adoption of universal 3-test strategy
47%
3-test strategy (n=21)
2-test strategy (n=24)
No information (n=2)
4. Policy adoption of retesting prior to ART initiation
51%
Retesting (n=23)
No retesting (n=21)
No information (n=3)
5. Policy adoption of discontinuation of Western Blot (WB)
78%
No WB (n=37)
Use of WB (n=10)
No information (n=2)
6. Policy adoption of dual HIV/Syphilis test in ANC
47%
Policy adopted (n=21)
No policy (n=24)
No information (n=2)

Supplementary Appendix

Country adoption of WHO 2019 guidance on HIV testing strategies and algorithms: a policy review across the WHO African region

Emmanuel Fajardo,[1] Céline Lastrucci,[1] Nayé Bah,[2] Casimir Manzengo Mingiedi,[3] Ndoungou Salla Ba,[4] Fausta Shakiwa Mosha,[5] Frank John Lule,[6] Margaret Alia Sampson Paul,[6] Lago Hughes,[6] Magdalena Barr-DiChiara,[1] Muhammad Shahid Jamil,[1] Anita Sands,[7] Rachel Baggeley,[1] Cheryl Johnson[1]

Supplementary Figure 1. Countries in the WHO African Region and grouping by subregion

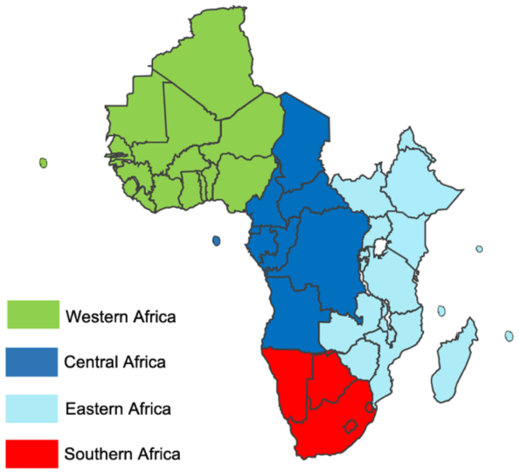

WHO African Region (n=47)			
Western Africa (n=17)	Central Africa (n=9)	Eastern Africa (n=16)	Southern Africa (n=5)
Algeria	Angola	Burundi	Botswana
Cabo Verde	Cameroon	Comoros	eSwatini
Burkina Faso	CAR	Eritrea	Lesotho
Benin	Chad	Ethiopia	Namibia
Ghana	DRC	Kenya	South Africa
Guinea	Equotorial Guinea	Madagascar	
Guinea-Bissau	Gabon	Malawi	
Ivory Coast	Republic of the Congo	Mauritius	
Liberia	Sao Tome and Principe	Mozambique	
Mauritania		Rwanda	
Mali		Seychelles	
Niger		South Sudan	
Nigeria		Tanzania	
Senegal		Uganda	
Sierra Leone		Zambia	
The Gambia		Zimbabwe	
Togo			

For peer review only

Supplementary Table 1. Categories for data extraction

**1. Policy Information**	
▪ Country<br>▪ WHO region<br>▪ HIV testing policy identified?	▪ Year of policy publication<br>▪ Type of policy document<br>▪ National HIV prevalence
**2. HIV testing algorithm (after 18 months of age)**	
▪ HIV testing algorithm provided?<br>▪ Image or text format?<br>▪ Type of scenario in which algorithm is used<br>▪ Number of assays used in algorithm<br>▪ Serial or parallel strategy<br>▪ Use of tiebreaker?<br>▪ Use of Western Blot or LIA?<br>▪ Mention of test order relating to sens/spec?<br>▪ High or low prevalence strategy used?<br>▪ Type of tests mentioned?<br>▪ Brand of tests mentioned?<br>▪ Are assays pre-qualified by WHO?<br>▪ A1 test name<br>▪ A1 type of assay	▪ A1 sens-/spec<br>▪ A2 test name<br>▪ A2 type of assay<br>▪ A2 sens-/spec<br>▪ A3 test name<br>▪ A3 type of assay<br>▪ A3 sens-/spec<br>▪ Source of assay sens/spec<br>▪ Mention of in-country assay validation<br>▪ Alignment with WHO policy?<br>▪ Primary reason(s) algorithm fails to meet WHO recommendations<br>▪ Other notes about testing strategy
**3. HIV testing algorithm among pregnant women using dual HIV/Syphilis RDTs**	
▪ HIV testing algorithm provided?<br>▪ Image or text format?<br>▪ Type of scenario in which algorithm is used<br>▪ Type of scenario in which algorithm should not be used (women on ART, already diagnosed/treated for syphilis, retesting for HIV)<br>▪ What is there syphilis testing strategy for women with HIV?<br>▪ Is dual test used as A1 or A0?<br>▪ Is the dual test algorithm aligned with the national HIV testing algorithm?<br>▪ Is the dual test algorithm aligned with the national syphilis testing algorithm?<br>▪ Brand of dual test mentioned?	▪ Is dual test prequalified by WHO?<br>▪ Dual test name<br>▪ Mention of penicillin treatment for reactive TP?<br>▪ Mention of further testing for reactive TP?<br>▪ Mention of in-country assay validation<br>▪ Alignment with WHO policy?<br>▪ Primary reason(s) algorithm fails to meet WHO recommendations<br>▪ Other notes about testing strategy
**4. Retesting prior to ART initiation**	
▪ Retest required before starting ART?<br>▪ Retest strategy same as national algorithm?<br>▪ Notes about retesting strategy	

For peer review only

Supplementary Table 2. List of national policies on HIV testing services in the WHO African Region collected in 2018 and 2021 by subregion

Subregion	Count	Country	Policy reviewed in 2018?	New policy or algorithm identified?	Publication year	Type of document	Language	Algorithm provided?	National HIV prevalence
Central Africa	1	Angola	Yes, 2015	Yes	2020	HTS	Portuguese	Yes	Low
	2	Cameroon	Yes, 2015	Yes	2019	ART	French	Yes	Low
	3	CAR	Yes, 2010	Yes	2018	ART	French	Yes	Low
	4	Chad	Yes, 2011	Yes	2017	HTS	French	Yes	Low
	5	DRC	Yes, 2017	Yes	2020	ART	French	Yes	Low
	6	Equatorial Guinea	No	Yes	2018	ART	Spanish	Yes	Low
	7	Gabon	No	Yes	2017	HTS	French	Yes	Low
	8	Republic of the Congo	No	**No**	–	–	–	–	–
	9	São Tomé e Principe	No	Yes	2018	Algorithm	Portuguese	Yes	Low
Eastern Africa	10	Burundi	Yes, 2016	Yes	2020	HTS	French	Yes	Low
	11	Comoros	Yes, 2007	Yes	2016	HTS	French	Yes	Low
	12	Eritrea	No	Yes	2019	HTS	English	Yes	Low
	13	Ethiopia	Yes, 2017	Yes	2018	ART	English	Yes	Low
	14	Kenya	Yes, 2017	Yes	2021	HTS	English	Yes	Low
	15	Madagascar	Yes, 2011	Yes	2018	HTS	French	Yes	Low
	16	Malawi*	Yes, 2016	Yes*	2016	HTS	English	Yes	High
	17	Mauritius	No	Yes	2020	HTS	English	Yes	Low
	18	Mozambique	Yes, 2016	Yes	2020	PMTCT	Portuguese	Yes	High
	19	Rwanda	Yes, 2016	Yes	2018	HTS	English	Yes	Low
	20	Seychelles	No	Yes	2019	HTS	English	Yes	Low
	21	South Sudan	Yes, 2017	Yes	2020	ART	English	Yes	Low
	22	Tanzania	Yes, 2017	Yes	2021	HTS	English	Yes	High
	23	Uganda	Yes, 2016	Yes	2020	ART	English	Yes	High
	24	Zambia	Yes, 2018	Yes	2020	HTS	English	Yes	High
	25	Zimbabwe*	Yes, 2016	Yes*	2016	Other	English	Yes	High
Southern Africa	26	Botswana**	Yes, 2016	**No**	2016	ART	English	Yes	High
	27	eSwatini**	Yes, 2018	**No**	2018	ART	English	Yes	High
	28	Lesotho	Yes, 2016	Yes	2021	PMTCT	English	Yes	High

	29	Namibia	Yes, 2016	Yes	2018	HTS	English	Yes	High
	30	South Africa	Yes, 2016	Yes	2020	ART	English	Yes	High
Western Africa	31	Algeria**	Yes, 2013	**No**	2013	HTS	French	Yes	Low
	32	Benin	No	Yes	2017	HTS	French	Yes	Low
	33	Burkina Faso	Yes, 2008	Yes	2021	ART	French	Yes	Low
	34	Cabo Verde	No	**No**	–	–	–	–	–
	35	Ghana	Yes, 2014	Yes	2019	ART	English	Yes	Low
	36	Guinea	No	Yes	2019	HTS	French	Yes	Low
	37	Guinea-Bissau	No	Yes	2021	ART	Portuguese	Yes	Low
	38	Ivory Coast**	Yes, 2016	**No**	2016	HTS	French	Yes	Low
	39	Liberia	Yes, 2015	Yes	2020	HTS	English	Yes	Low
	40	Mauritania	No	Yes	2020	HTS	French	Yes	Low
	41	Mali	No	Yes	2017	HTS	French	Yes	Low
	42	Niger	No	Yes	2020	HTS	French	Yes	Low
	43	Nigeria	Yes, 2016	Yes	2020	ART	English	Yes	Low
	44	Senegal	Yes, 2017	Yes	2018	HTS	French	Yes	Low
	45	Sierra Leone	Yes, 2017	Yes	2020	ART	English	Yes	Low
	46	The Gambia	Yes, 2014	Yes	2019	ART	English	Yes	Low
	47	Togo	No	Yes	2019	HTS	French	Yes	Low

*These countries provided an updated HIV testing algorithm despite having an old HIV testing guideline

** These countries did not provide an update HIV testing guideline but were included in the analysis

For peer review only

Supplementary Table 3. Progress on policy adoption in the WHO African Region from 2018 to 2021

Subregion	Count	Country	Policy reviewed in 2018?	Adherence in 2018	Policy reviewed in 2021	Adherence in 2021
Central Africa	1	Angola	Yes, 2015	Not adopted	2020	Mostly adopted
	2	Cameroon	Yes, 2015	Not adopted	2019	Mostly adopted
	3	CAR	Yes, 2010	Not adopted	2018	Not adopted
	4	Chad	Yes, 2011	Not adopted	2017	Mostly adopted
	5	DRC	Yes, 2017	Adopted	2020	Adopted
	6	Equatorial Guinea	No	–	2018	Not adopted
	7	Gabon	No	–	2017	Mostly adopted
	8	Republic of the Congo	No	–	–	–
	9	São Tomé e Principe	No	–	2018	Not adopted
Eastern Africa	10	Burundi	Yes, 2016	Not adopted	2020	Mostly adopted
	11	Comoros	Yes, 2007	No information	2016	Not adopted
	12	Eritrea	No	–	2019	Not adopted
	13	Ethiopia	Yes, 2017	No information	2018	Mostly adopted
	14	Kenya	Yes, 2017	Mostly adopted	2021	Adopted
	15	Madagascar	Yes, 2011	No information	2018	Mostly adopted
	16	Malawi	Yes, 2016	Not adopted	2016	Mostly adopted
	17	Mauritius	No	–	2020	Not adopted
	18	Mozambique	Yes, 2016	Not adopted	2020	Mostly adopted
	19	Rwanda	Yes, 2016	Not adopted	2018	Mostly adopted
	20	Seychelles	No	–	2019	Not adopted
	21	South Sudan	Yes, 2017	Not adopted	2020	Adopted
	22	Tanzania	Yes, 2017	Not adopted	2021	Mostly adopted
	23	Uganda	Yes, 2016	Mostly adopted	2020	Mostly adopted
	24	Zambia	Yes, 2018	Not adopted	2020	Not adopted
	25	Zimbabwe	Yes, 2016	Adopted	2016	Adopted
Southern Africa	26	Botswana	Yes, 2016	Mostly adopted	2016	Not adopted
	27	eSwatini	Yes, 2018	Not adopted	2018	Not adopted
	28	Lesotho	Yes, 2016	Adopted	2021	Mostly adopted
	29	Namibia	Yes, 2016	No information	2018	Not adopted
	30	South Africa	Yes, 2016	Mostly adopted	2020	Not adopted
Western Africa	31	Algeria	Yes, 2013	Adopted	2013	Not adopted
	32	Benin	No	–	2017	Not adopted
	33	Burkina Faso	Yes, 2008	Not adopted	2021	Mostly adopted
	34	Cabo Verde	No	–	–	–
	35	Ghana	Yes, 2014	Not adopted	2019	Mostly adopted
	36	Guinea	No	–	2019	Mostly adopted
	37	Guinea-Bissau	No	–	2021	Not adopted
	38	Ivory Coast	Yes, 2016	Not adopted	2016	Not adopted
	39	Liberia	Yes, 2015	Not adopted	2020	Mostly adopted
	40	Mauritania	No	–	2020	Not adopted
	41	Mali	No	–	2017	Not adopted
	42	Niger	No	–	2020	Not adopted
	43	Nigeria	Yes, 2016	Not adopted	2020	Mostly adopted
	44	Senegal	Yes, 2017	Mostly adopted	2018	Not adopted
	45	Sierra Leone	Yes, 2017	Not adopted	2020	Not adopted
	46	The Gambia	Yes, 2014	Not adopted	2019	Not adopted
	47	Togo	No	–	2019	Not adherent

A total of 32 countries had policies reviewed in 2018 and 2021. Those highlighted in red are policies reviewed in 2018 that were updated (n=28). Number of policies deemed adopted in 2018 (n=7) and number deemed adopted in 2021 (n=20)

For peer review only - http://bmjopen.bmj.com/site/about/guidelines.xhtml

Supplementary Figure 2. Comparing policy adoption across countries, 2018 vs 2021

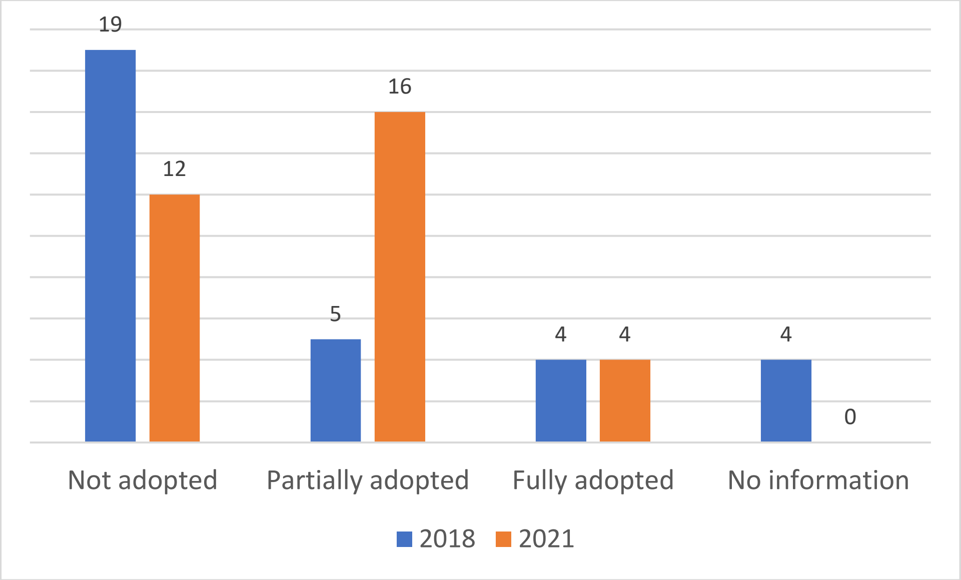

For peer review only

Supplementary Table 4. Policy adoption of 2019 WHO-recommended HIV testing strategies in the WHO African region, 2014-2021

AFRO subregions (countries)	Policies reviewed			Serial testing strategy			Discontinuation of tiebreaker* testing strategy			Retesting prior to ART initiation		Universal 3-test strategy	Use of dual HIV/syphilis test**	No use of Western Blot
Year	2014	2018	2021	2014	2018	2021	2014	2018	2021	2018	2021	2021	2021	2021
All (n=47)	25 (53%)	32 (68%)	45 (96%)	21 (84%)	26 (81%)	29 (64%)	15 (60%)	26 (81%)	37 (82%)	16 (50%)	23 (51%)	21 (47%)	21 (47%)	35 (78%)
Western (n=17)	6 (35%)	9 (53%)	16 (94%)	5 (83%)	7 (78%)	9 (56%)	4 (67%)	5 (56%)	9 (56%)	1 (11%)	5 (31%)	8 (50%)	7 (44%)	10 (63%)
Central (n=9)	4 (44%)	5 (56%)	8 (89%)	4 (100%)	5 (100%)	7 (88%)	3 (75%)	4 (80%)	7 (88%)	1 (20%)	5 (63%)	3 (38%)	2 (25%)	6 (75%)
Eastern (n=16)	11 (69%)	13 (81%)	16 (100%)	10 (91%)	10 (77%)	12 (75%)	5 (45%)	12 (92%)	16 (100%)	9 (69%)	9 (56%)	9 (56%)	9 (56%)	14 (88%)
Southern (n=5)	4 (80%)	5 (100%)	5 (100%)	2 (40%)	4 (80%)	1 (20%)	3 (75%)	5 (100%)	5 (100%)	5 (100%)	4 (80%)	1 (20%)	3 (60%)	5 (100%)

*Use of a third assay to rule-in HIV infection

**Dual HIV/syphilis RDT as first test in ANC

For peer review only - http://bmjopen.bmj.com/site/about/guidelines.xhtml

Supplementary Table 5. Short name of products used as part of the 2-assay or 3-assay HIV testing algorithm in 28 HIV national testing policy documents reviewed in 2021

Strategy	Count	Country	Assay 1	Assay 2	Assay 3
2-assay	1	Angola	Determine	Uni-Gold	–
	2	Cameroon	Determine or Uni-Gold	OraQuick or Shanghai	–
	3	CAR	Determine	Uni-Gold	–
	4	Cote D'Ivoire	Determine	SD Bioline or GenieFast	StatPak or EIA (as tiebreaker)
	5	Equatorial Guinea	Determine	Hexagon	Uni-Gold (for discordants)
	6	eSwatini	Determine	Uni-Gold	–
	7	Gambia	Determine	SD Bioline or First Response	–
	8	Guinea Bissau	Determine	First Response	PCR (as tiebreaker)
	9	Mali	Alere Combo	SD Bioline	First Response (as tiebreaker)
	10	Mozambique	Determine	Uni-Gold	–
	11	Rwanda	Alere Combo	StatPak	–
	12	São Tomé e Principe	Determine	SD Bioline	PCR (as tiebreaker)
	13	Sierra Leone	Determine	SD Bioline	Uni-Gold (as tiebreaker)
	14	Uganda	Determine	StatPak	SD Bioline (for discordants)
	15	Nigeria	Determine<br>Uni-Gold<br>Stat-Pak<br>Double Check Gold<br>Sure Check<br>HIV Quick Check	Determine<br>Uni-Gold<br>Stat-Pak<br>Double Check Gold<br>Sure Check<br>HIV Quick Check	Determine<br>Uni-Gold<br>Stat-Pak<br>Double Check Gold<br>Sure Check<br>HIV Quick Check<br>(as tiebreaker)
	16	Togo	Murex HIV Ag/Ab<br>Apdia HIV Ag/Ab<br>Determine<br>Alere Combo<br>Vikia HIV 1/2<br>Wanta Rapid Test<br>ABON HIV 1/2/0<br>Standard Q<br>Hexagon<br>Genie Fast<br>SD Bioline<br>First Response<br>SD Bioline HIV/Syphilis<br>OraQuick HIV Self-Test	ABON HIV 1/2/0<br>Standard Q<br>SD Bioline<br>First Response<br>HIV Tri-Dot	INNOLIA or Genius (as tiebreaker)
3-assay	17	Burundi	Alere Combo or Determine	SD Bioline or Uni-Gold	Wondfo One Step
	18	Gabon	Alere Combo	Determine	SD Bioline
	19	Ghana	First Response	OraQuick	SD Bioline
	20	Guinea	Determine	SD Bioline	Multisure
	21	Kenya	INSTI	Uni-Gold	SD Bioline
	22	Liberia	Determine	SD Bioline	Uni-Gold
	23	Madagascar	Determine	Uni-Gold	SD Bioline
	24	Mauritius	GenScreen Ultra Ag/Ab	Alere Combo	Western Blot
	25	Malawi	INSTI	Uni-Gold	SD Bioline

For peer review only - http://bmjopen.bmj.com/site/about/guidelines.xhtml

	26	Niger	Alere Combo	Wondfo One Step	SD Bioline
	27	Senegal	Determine	SD Bioline	Multisure
	28	Zimbabwe	Determine	Chembio	INSTI
	29	Burkina Faso	3rd generation:<br>Determine<br>Double check Gold Ultra<br>Onsite HIV1+2 Plus<br>Combo<br>VIKIA HIV 1/2<br>4th generation:<br>Alere Ag/Ab Combo<br>OnSite HIV Ab/Ag (CTK)	HIV TriDot<br>ImmunoFlow<br>OnSite HIV 1/2 Ab Plus (CTK)<br>SD Bioline	Any of those not selected as A1 or A2

For peer review only

For peer review only - http://bmjopen.bmj.com/site/about/guidelines.xhtml

BMJ Open

Country adoption of WHO 2019 guidance on HIV testing strategies and algorithms: a policy review across the WHO African region

Journal:	*BMJ Open*
Manuscript ID	bmjopen-2022-071198.R2
Article Type:	Original research
Date Submitted by the Author:	31-Oct-2023

Complete List of Authors:	Fajardo, Emmanuel; World Health Organization, Global HIV, Hepatitis, and STI Programmes<br>Lastrucci, Céline; WHO, Global HIV, Hepatitis, and STI Programmes<br>Bah, Nayé; World Health Organization Regional Office for Africa<br>Mingiedi, Casimir Manzengo; World Health Organization Regional Office for Africa, Inter-country support team for Central Africa<br>Ba, Ndoungou Salla; World Health Organization Regional Office for Africa, Inter-country support team for Western and Central Africa<br>Mosha, Fausta; World Health Organization Regional Office for Africa, Inter-country support team for Eastern and Southern Africa<br>Lule, Frank John; World Health Organization Regional Office for Africa<br>Paul, Margaret Alia Sampson; World Health Organization Regional Office for Africa<br>Hughes, Lago; World Health Organization Regional Office for Africa<br>Barr-DiChiara, Magdalena; World Health Organization, Department of Global Programmes of HIV, Hepatitis and HIV<br>Jamil, Muhammad; World Health Organization, Global HIV, Hepatitis, and STI Programmes<br>Sands, Anita ; World Health Organization, Regulation and Prequalification<br>Baggaley, R; World Health Organization<br>Johnson, Cheryl; WHO

**Primary Subject Heading**:	Health policy
Secondary Subject Heading:	HIV/AIDS
Keywords:	Health policy < HEALTH SERVICES ADMINISTRATION & MANAGEMENT, HIV & AIDS < INFECTIOUS DISEASES, Diagnostic microbiology < INFECTIOUS DISEASES

SCHOLARONE™
Manuscripts

I, the Submitting Author has the right to grant and does grant on behalf of all authors of the Work (as defined in the below author licence), an exclusive licence and/or a non-exclusive licence for contributions from authors who are: i) UK Crown employees; ii) where BMJ has agreed a CC-BY licence shall apply, and/or iii) in accordance with the terms applicable for US Federal Government officers or employees acting as part of their official duties; on a worldwide, perpetual, irrevocable, royalty-free basis to BMJ Publishing Group Ltd ("BMJ") its licensees and where the relevant Journal is co-owned by BMJ to the co-owners of the Journal, to publish the Work in this journal and any other BMJ products and to exploit all rights, as set out in our licence.

The Submitting Author accepts and understands that any supply made under these terms is made by BMJ to the Submitting Author unless you are acting as an employee on behalf of your employer or a postgraduate student of an affiliated institution which is paying any applicable article publishing charge ("APC") for Open Access articles. Where the Submitting Author wishes to make the Work available on an Open Access basis (and intends to pay the relevant APC), the terms of reuse of such Open Access shall be governed by a Creative Commons licence – details of these licences and which Creative Commons licence will apply to this Work are set out in our licence referred to above.

Other than as permitted in any relevant BMJ Author's Self Archiving Policies, I confirm this Work has not been accepted for publication elsewhere, is not being considered for publication elsewhere and does not duplicate material already published. I confirm all authors consent to publication of this Work and authorise the granting of this licence.

For peer review only

Title: Country adoption of WHO 2019 guidance on HIV testing strategies and algorithms: a policy review across the WHO African region

Authors: Emmanuel Fajardo,[1] Céline Lastrucci,[1] Nayé Bah,[2] Casimir Manzengo Mingiedi,[3] Ndoungou Salla Ba,[4] Fausta Shakiwa Mosha,[5] Frank John Lule,[6] Margaret Alia Samson Paul,[6] Lago Hughes,[6] Magdalena Barr-DiChiara,[1] Muhammad Shahid Jamil,[1] Anita Sands,[7] Rachel Baggaley,[1] Cheryl Johnson[1]

Authors affiliations:

[1]Global HIV, Hepatitis and STI Programmes, WHO, Switzerland

[2]Regional Office for Africa, WHO Country Office, Mali

[3]Regional Office for Africa, Inter-country support team for Central Africa, WHO, Gabon

[4]Regional Office for Africa, Inter-country support team for Western and Central Africa, WHO, Burkina Faso

[5]Regional Office for Africa, Inter-country support team for Eastern and Southern Africa, WHO Zimbabwe

[6]Regional Office for Africa, WHO Congo Brazzaville

[7]Incidents and Substandard/Falsified Medical Products Team, Regulation and Prequalification Department, WHO, Switzerland

Corresponding author: Cheryl Johnson, Global HIV, Hepatitis and STI Programmes, WHO, Avenue Appia 20, 1211 Geneva, Switzerland. johnsonc@who.int

For peer review only - http://bmjopen.bmj.com/site/about/guidelines.xhtml

ABSTRACT

Objectives: In 2019, the World Health Organization (WHO) released guidelines on HIV testing services (HTS). We aim to assess adoption of six of these recommendations on HIV testing strategies among African countries.

Design: Policy review

Setting: 47 countries within the WHO African region.

Participants: National HTS policies from the WHO African region as of December 2021.

Primary and secondary outcome measures: Uptake of WHO recommendations across national HTS policies including the standard 3-test strategy; discontinuation of a tie-breaker test to rule in HIV infection; discontinuation of western blotting (WB) for HIV diagnosis; retesting prior to ART initiation; and the use of dual HIV/syphilis rapid diagnostic tests (RDTs) in antenatal care (ANC). Country policy adoption was assessed on a continuum, based on varying levels of complete adoption.

Results: National policies were reviewed for 96% (n=45/47) of countries in the WHO African region, 38% (n=18) were published before 2019, and 60% (n=28) adopted WHO guidance. Among countries that had not fully adopted WHO guidance, not yet adopting a 3-test strategy was the most common reason for misalignment (45%, 21/47); of which 31% and 22% were in low- (<5%) and high-prevalence (≥5%) countries respectively. Ten policies (21%) recommended the use of WB and 49% (n=23) recommended retesting before ART initiation. Dual HIV/syphilis RDTs were recommended in 45% (n=21/47) of policies.

Conclusions: Many countries in the African region have adopted WHO-recommended HIV testing strategies, however efforts are still needed to fully adopt WHO guidance. Countries should accelerate their efforts to adopt and implement a 3-test strategy, retesting prior to ART initiation and the use of dual HIV/syphilis RDTs.

Keywords: HIV, diagnosis, rapid test, dual HIV/syphilis, policy, guideline, Africa, algorithm

Strengths and limitations of this study

- This study provided a comprehensive review of existing national HTS policies in the African region and was able to identify current policies for 45 countries.
- Data was collected using a robust search and global repository developed by WHO, as well as a standardised extraction tool to synthesise national policy information.
- The review focused on published policies and did not assess country implementation at the national, subnational, or site level.
- The review focused on WHO recommendations related to HIV testing strategies for individuals ≥18 months of age.
- The review identified African countries which reported that they were in the process of adopting WHO recommendations – particularly the use of 3-test strategy and dual HIV/syphilis RDTs.

INTRODUCTION

HIV Testing Services (HTS) is the critical gateway to accessing HIV-related care and treatment for those diagnosed as HIV-positive and as a means to accessing prevention services for those who test HIV-negative.[1, 2] Despite considerable progress, reaching the UNAIDS 95-95-95 targets by 2025 will require increased testing uptake, particularly among populations with testing gaps such as key population, youth and men.[3] Further, the global emergency caused by the COVID-19 pandemic in 2020 has disrupted many health services including HIV testing services, and supply chains of key commodities such as rapid diagnostic tests (RDTs) have

been stretched.[4] In 2021, it was estimated that 85% of people living with HIV globally were aware of their serostatus.[5]

In 2019, the World Health Organization (WHO) published consolidated guidelines on HIV testing services (HTS),[6] bringing together existing and new recommendations specific to testing strategies and algorithms. This includes existing guidance on the use of serial testing rather than parallel testing (WHO, 2012),[7] discontinuation of a tiebreaker strategy to rule-in HIV infection (WHO, 2015),[1] and retesting prior to antiretroviral treatment (ART) initiation, recommended since 2014.[1] As well as three new recommendations on: (1) the use of a standard 3-test strategy which uses three consecutive HIV-reactive tests to provide and HIV-positive diagnosis, (2) the discontinuation of western blot (WB) and line immunoassays (LIA) and, (3) the use of dual HIV/syphilis RDTs as first assay in HTS in antenatal care (ANC). In light of these testing modalities, ensuring that HIV testing is accessible and adheres to the "5 C's" including voluntary consent, confidentiality, counselling, correct results, and linkage to care, is of upmost importance.

These recommendations have been prioritised because they are essential to achieving global 95-95-95 goals by making testing accurate, affordable and high impact. [3,6] By moving away from testing services with WB, programmes will no longer have long turnaround times which delay the ability to offer same day ART or PrEP initiation.[6] Adoption of innovative tools such as the dual HIV/syphilis RDTs will enable more people to be tested and treated for syphilis, which is essential for achieving triple elimination goals.[6] Additionally, many countries still use the 2015 WHO recommendation of using the national HIV prevalence to determine whether a two-assay (≥5%) or three-assay testing strategy (<5%) should be used.[1] However, as ART coverage has expanded HIV positivity and the proportion of people with HIV who are

For peer review only - http://bmjopen.bmj.com/site/about/guidelines.xhtml

undiagnosed and not in care will continue to decline. As a result, the positive predictive value of previous testing strategies will also decline and lead to an increase in false positive diagnoses. In relation to the guideline development process, WHO now recommends all countries use a standard 3-test strategy.[8] WHO also recommends that countries planning to update their HIV testing algorithms undertake a verification study to select appropriate HIV serology products and ensure they do not cross-react in order to minimize the risk of misdiagnosis.[9]

Box 1. WHO recommendations for HIV testing strategies for individuals ≥18 months of age, 2019

- Western blotting and line immunoassays should not be used in national HIV testing strategies and algorithms.
- Dual HIV/syphilis RDTs can be the first test in HIV testing strategies and algorithms in ANC settings.
- In response to changes in the HIV epidemic, WHO recommends countries use three consecutive reactive tests to provide an HIV-positive diagnosis.
- Testing strategies should use tests serially, not in parallel, and should not use a tiebreaker to rule-in HIV infection. Instead, those with discrepant test results should be ruled inconclusive and referred for further testing in 14 days to rule-in or rule-out seroconversion.
- WHO recommends that all HIV testing algorithms use a combination of RDTs and/or EIAs to achieve at least 99% positive predictive value and use a combination of tests with ≥99% sensitivity and ≥98% specificity. WHO does not recommend the use of NAT techniques within HIV testing algorithms for individuals ≥18 months of age.

For peer review only - http://bmjopen.bmj.com/site/about/guidelines.xhtml

- The first test in an HIV testing strategy and algorithm should have the highest sensitivity, followed by a second and third test of the highest specificity. Algorithms should be validated and verified to ensure high quality and accurate testing.
- All people newly diagnosed with HIV should be retested to verify their HIV status prior to starting ART, using the same testing strategy and algorithm as the initial test. To minimize the risk of misdiagnosis, this approach should be maintained in settings in which rapid ART initiation is being implemented.

Source: WHO 2019, [6]

To support the implementation of the 2019 guidelines, WHO launched and disseminated the guidance at the Africa Society of Laboratory Medicine and the International Conference on AIDS and STIs in Africa. Following this, WHO also provided detailed country support to adopt the guidelines along with developing an application to access the guidelines more easily. In addition to dissemination and country support, [10] it is critical to monitor and track the implementation of WHO policy uptake to understand policy priorities and challenges overtime. Such tracking not only provides valuable insights but can help guide revisions to future guidance and can help target country policy support [11—12].

A global review of national HIV testing policies was conducted by WHO in 2018 to assess adoption of its testing recommendations and policies.[13] Of 91 policies reviewed only 24 (25%) adhered to WHO guidance. This policy review follows on from the previous review and seeks to assess country uptake of six 2019 WHO recommendations related to HIV testing strategies. Here we focus on the WHO African region, which hosts several countries with a high HIV burden and with large HIV testing programmes. The same policy review across the

For peer review only - http://bmjopen.bmj.com/site/about/guidelines.xhtml

remaining five WHO regions is underway as part of the update to the 2023 WHO HTS guidelines.

METHODS

Search strategy

We carried out a comprehensive desk review of national HIV testing policies in 47 Member States in the WHO African region (online supplementary figure 1) using a policy repository maintained by WHO, scanning government websites, and by contacting government officials or other relevant informants through December 2021. The most current policy documents containing information on the HTS policy were included, but when this was unavailable, we included the most recent HIV testing algorithm, but if that was also unavailable, we opted to include previous national policies. Documents of all languages were included. Other supporting documents related to algorithm validation and PMTCT guidelines were identified, when possible, either through references provided in the national HTS policy or through contact with key informants. The full protocol for the review was previously developed and published in 2018. [13]

Data extraction

Data were extracted from each policy document by one reviewer (EF) into standardised coding forms on policy information, HIV testing strategy/algorithm (>18 months of age), dual HIV/syphilis testing strategy/algorithm, and retesting prior to ART initiation (online supplementary table 1). When reported the order, type of test kits and name brand of diagnostics used within the HIV testing algorithm was summarised descriptively, as well as information on whether the algorithm was verified or validated.

A second reviewer (CL) carried out crosschecking of the data. Differences between coders were resolved through a third reviewer (CJ). To prevent misclassification, items were marked as 'unclear' during data extraction when lack of information prevented complete understanding. Reviewers than worked to contact key informant to provide further detail and clarity wherever possible.

Analysis

We assessed national adoption of WHO HTS guidance using six specific recommendations related to testing strategies and algorithms for those ≥18 months of age set forth in the WHO 2019 HIV testing guidelines, namely: (1) use of serial testing, (2) use of a 3-test strategy, (3) discontinuation of a tie-breaker to rule in HIV infection, (4) discontinuation of WB/LIA, (5) retesting prior to ART initiation, and (6) use of dual HIV/syphilis RDT in ANC. Additional qualitative details about the national testing algorithm, such as the order of and name brand of test kits used in testing algorithms, were also reviewed when reported and assessed according to WHO recommendations (see Box 1).

Based on the number of recommendations adopted, national policies were categorized as: adopted (6); nearly adopted (5 to 4); somewhat not adopted: (3); and not adopted: (2 or less). We then provide simplified reporting (adopted, partially adopted and not adopted) for national policies which reviewed in 2018 and 2021 to assess changes overtime. Notably, these categories were initially developed as part of the 2018 policy review and maintained to assist with future updates and policy tracking.

For peer review only - http://bmjopen.bmj.com/site/about/guidelines.xhtml

Descriptive analyses were then stratified by subregions (Western, Central, Eastern and Southern Africa; online supplementary figure 1) to determine rates of adherence. All analyses were conducted in Microsoft Excel. Countries which had policies reviewed in 2018 and 2021 were also compared to assess changes in alignment with WHO recommendations over time.

Patient and public involvement

Patients and/or the public were not involved in the design, conduct, reporting, or dissemination plans of this study.

RESULTS

In total, we were able to identify policy documents from 96% of countries from the WHO African region (n=45/47); 71% of countries had a policy reviewed in both 2018 and 2021 (n=32/45). Two countries, Republic of the Congo and Cabo Verde, were not included because we were unable to obtain their national policies and sufficient information (figure 1). Out of all these policies, 91% (41/45) had updated their national policies as of December 2021. Policy publication dates ranged from 2013 to 2021. Out of the six country policies that had not been fully updated since 2018 (Algeria, Botswana, eSwatini, Cote d'Ivoire, Malawi, and Zimbabwe), one country (Zimbabwe) provided a new HIV testing algorithm that was included in the review.

Of the 45 policies providing information on HIV testing strategies, 16 were from Western Africa (36%), 16 were from Eastern Africa (36%), 8 were from Central Africa (18%), and 5 were from Southern Africa (11%). Policies were published in English (n=22), French (n=18), Portuguese (n=4) and Spanish (n=1). Based on the most recent national HIV prevalence

reported by UNAIDS [1], 34 countries (76%) had a low HIV prevalence (<5%) and 11 countries (24%) had a high HIV prevalence (≥5%) (online supplementary table 2).

Overall adoption of WHO-recommended HIV testing strategies

In 2021, 59% of national testing strategies (n=28/45) were either fully or partially aligned with WHO 2019 HTS recommendations and 36% (n=17/47) had not adopted the recommendations (table 1). When the analysis was restricted to a subset of countries with policies in both the 2018 and 2021 review (n=32), the proportion adopting WHO guidance in 2018 was 28% (n=9/32) *vs* 62% (n=20/32) in the current (2021) review (see online supplementary table 3; supplementary figure 2). Across the sub-regions, Southern (80%) and Eastern (75%) Africa had the greatest level of alignment, whilst Central Africa (63%) and Western Africa (44%) had the lowest adoption rates (table 1; figure 2).

In low-prevalence countries (n=34), 53% of policies (n=18/34) had fully or mostly adopted the guidance and 47% (n=16/34) had generally not adopted guidance. The most common reasons for non-adoption were not using a dual HIV/syphilis RDT in ANC (n=15/34), no retesting prior to ART initiation (n=14/34) and using only two assays to make an HIV-positive diagnosis (n=10/34). In high-prevalence countries (n=11), 91% of policies (n=10/11) had fully or nearly adopted WHO guidance and 9% had not adopted WHO guidance (1/11). The most common reasons for not fully adopting WHO guidance were continuing to use a 2-test strategy to make an HIV-positive diagnosis (n=10/11), employing both serial and parallel testing strategies (n=4/11), not adopting dual HIV/syphilis RDTs in ANC (n=4/11) and not implementing retesting prior to ART initiation (n=3/11). Overall, only one out of eleven high HIV-burden countries, Zimbabwe, recommended the use of a 3-assay testing strategy (18%).

Table 1. Policy adoption of 2019 WHO-recommended HIV testing strategies in the WHO African region, 2021

AFRO subregions (countries)	Policies reviewed	Serial testing strategy	Discontinuation of tiebreaker* testing strategy	Retesting prior to ART initiation	Recommended 3-test strategy**	Use of dual HIV/syphilis test***	No use of Western Blot
**All (n=47)**	45 (96%)	29 (62%)	37 (79%)	23 (49%)	21 (45%)	21 (45%)	35 (74%)
**Western (n=17)**	16 (94%)	9 (53%)	9 (53%)	5 (29%)	8 (47%)	7 (41%)	10 (59%)
**Central (n=9)**	8 (89%)	7 (78%)	7 (78%)	5 (56%)	3 (33%)	2 (22%)	6 (67%)
**Eastern (n=16)**	16 (100%)	12 (75%)	16 (100%)	9 (56%)	9 (56%)	9 (56%)	14 (88%)
**Southern (n=5)**	5 (100%)	1 (20%)	5 (100%)	4 (80%)	1 (20%)	3 (60%)	5 (100%)

*Use of a third assay to rule-in HIV infection

**At the time of the review some countries noted that they were in the process of transitioning to a 3-test strategy, however they were not included as in compliance because their national guidance and testing strategy was not yet finalized.

**Dual HIV/syphilis RDT as first test in ANC. Note that at the time of the review, five countries were using test outside of WHO guidance at the time. Two countries had non-compliant algorithms (Lesotho and Zambia) and three countries (Liberia, Madagascar and Uganda) were using the test outside pregnant women and among key populations and partners of pregnant women. However, as of 2023, use of the dual test is recommended among key populations and those with high ongoing risk.

For peer review only - http://bmjopen.bmj.com/site/about/guidelines.xhtml

Adoption of serial testing strategy

In 2021, 62% of national testing strategies (n=29/47) adopted the use of serial testing and 36% (n=16/47) recommended the mix use of serial and parallel testing (figure 3_1). The subregions with the highest adoption were Central (78%) and Eastern Africa (75%), followed by Western Africa (38%); Southern Africa had the lowest adoption rate (20%) (table 1). However, fewer countries in southern Africa recommended serial testing in 2021 (n=1) than in 2018 (n=4) (supplementary table 4). Most policies recommending serial or parallel testing (n=15/16) conducted simultaneous testing of assay 1 (A1) and assay 2 (A2) in case of discrepant test results, and the Namibian policy recommended parallel testing of A2 and A3 after a reactive A1.

Only 43% of policies (n=20/47) provided guidance on the assay order in relation to their sensitivity and specificity. The proportion of HIV testing strategies/algorithms using two consecutive HIV-reactive tests to make an HIV-positive diagnosis was 51% (n=24/47) whereas 45% (n=21/47) recommended the use 3 consecutive HIV-reactive tests.

Sixty-four percent of the HIV testing strategies reviewed (n=29/45) included information on the testing algorithms, i.e., contained specific product names and all of them included WHO prequalified products. Determine HIV 1/2 (Abbott Diagnostics Medical Co., Ltd, Japan) was the most common product used as A1 and Bioline HIV 1/2 3.0 (Abbott Diagnostics Korea Inc., Republic of Korea) or Uni-Gold HIV (Trinity Biotech, Ireland) as A2 or A3 (see online supplementary table 5). Only 31% of reviewed policies (n=14/45) mentioned the need to locally verify or validate the HIV testing algorithm.

Discontinuation of a tiebreaker testing strategy

The discontinuation of a third assay to rule in HIV infection (i.e., tiebreaker) was recommended in 78% of national policies (n=37/47) (figure 3_2). The subregions with the highest adoption were in Southern Africa and Eastern Africa (100%, each) followed by Central Africa (78%), whereas Western Africa had the lowest adoption (53%) (table 1).

Of the eight policies recommending a tiebreaker testing strategy, seven were from Western Africa and one from Central Africa. Seven policies specified the type of A3 to be used, for example, RDT (n=4), RDT or EIA (n=1), nucleic acid testing (NAT: n=2) and LIA (n=1).

Adoption of WHO standard 3-assay testing strategy

The use of three consecutive HIV-reactive tests for the diagnosis of HIV was recommended by 45% of the policies (n=21/47) (figure 3_3). The adoption of this WHO recommendation was highest in Eastern Africa (56%) and Western Africa (47%) and lowest in Southern Africa (20%) (table 1). Most policies recommending a 3-test strategy (n=20/21) were from low HIV-prevalence countries. Among high HIV-prevalence countries, only one out of eleven countries (9%) officially recommended the use of a 3-test strategy, which was Zimbabwe.

Overall, across countries which had policies reviewed in both 2018 and 2021, 9 policies that previously recommended a 2-test strategy transitioned to a 3-test strategy as of 2021 (Burkina Faso, Burundi, Chad, Ethiopia, Ghana, Kenya, Lesotho, Madagascar, and Zimbabwe, respectively).

For peer review only - http://bmjopen.bmj.com/site/about/guidelines.xhtml

Further, we identified that twenty-one percent of national policies (n=10) recommended the use of NAT as part of the testing algorithm for the diagnosis of HIV infection in individuals ≥18 months of age. Four policies were from Eastern Africa (Malawi, Mauritius, Mozambique and United Republic of Tanzania); three national policies from Southern Africa (Eswatini, Lesotho and Namibia); two policies from Central Africa (Angola and Sao Tomé et Principe); and one national policy from Western Africa (Guinea Bissau). While generally not in alignment with WHO guidance, two policies employed NAT as the third assay (A3) of the algorithm while the remaining eight policies recommended NAT to resolve persistent inconclusive/indeterminate test results (referring either to viral load, PCR or DNA-PCR).

Adoption of retesting to verify positive status prior to ART initiation

In 2021, 49% of national strategies (n=23/47) recommended retesting prior to ART initiation (figure 3_4). The regions with the highest level of policy adoption were in Southern Africa (80%) followed by Central (56%) and Eastern Africa (56%) (table 1). However, fewer countries in southern Africa recommended retesting before ART initiation in 2021 (n=4) than in 2018 (n=5) (supplementary table 5). Sixty-five percent of the retesting policies (n=15/23) recommended retesting using the same national HIV testing algorithm in a new specimen run by a different operator in a different testing site; 26% (n=6/23) did not specify how retesting was carried out and 9% of policies (n=2/23) utilized retesting strategies that differed from the national HIV testing algorithm, which included parallel testing of A1/A2 in Botswana and Malawi.

Discontinuation of Western Blotting

The proportion of national policies not including WB or LIA as part of the HIV testing algorithm was 74% (n=35/47) (figure 3_5). This proportion was highest in Southern Africa (100%) and

Eastern Africa (88%) followed by Central Africa (67%) and Western Africa (59%) (table 1). Most countries with a supporting policy in favour of WB or LIA (n=9/10) recommended its use to manage persistent inconclusive/indeterminate test results and one policy used WB as A3 (Mauritius).

Adoption of dual HIV/syphilis RDT in ANC settings

Forty-five percent (n=21/47) of policies reviewed supported the use of dual HIV/syphilis RDTs among pregnant women in ANC as recommended by WHO (figure 3_6). The region with the highest policy uptake was Southern Africa (60%), followed by Eastern (56%), Western (41%) and Central Africa (22%) (table 1). And of policies supporting a 3-test strategy, only 53% (n=11/21) also recommended the use of dual HIV/syphilis RDTs among pregnant women. The majority of policies (n=20/21) recommended the use of dual HIV/syphilis RDT as A1 among pregnant women and only one policy (Lesotho) recommended its use as a triage test (A0) as an interim algorithm while waiting for the results of a national verification study.

The dual HIV/syphilis testing algorithm was not aligned with the national HIV testing algorithm in one country (Zambia): while Determine HIV-1/2 (Abbott Diagnostics Medical Co., Ltd, Japan) and SD Bioline HIV-1/2 3.0 (Abbott Diagnostics Korea Inc, Republic of Korea) products were used as A1 and A2 in the national testing algorithm, Bioline HIV/Syphilis Duo (Abbott Diagnostics Korea Inc, Republic of Korea) and Rapid Test for Antibody to Human Immunodeficiency Virus (HIV) (Colloidal Gold Device) (Beijing Wantai Biological Pharmacy Enterprise Co., Ltd., China) were used as A1 and A2 in the ANC testing algorithm.

In addition to the use in ANC settings as recommended by WHO in 2019, three countries also recommended testing with dual HIV/syphilis RDTs among other populations such as male partners of pregnant women and key populations (Liberia, Madagascar and Uganda). Only 50% of policies (n=11/21) specified the brand name of the dual HIV/syphilis RDT in the testing algorithm, and among the policies mentioning the brand name, Bioline HIV/Syphilis Duo (Abbott Diagnostics Korea Inc, Republic of Korea) was the most employed RDT (100%). Only 57% of policies (n=12/21) mentioned whether syphilis treatment was provided immediately after a reactive syphilis test result.

DISCUSSION

The current review found a significant improvement in the overall policy uptake of the latest WHO-recommended HIV testing strategies with 60% of countries in the African region adopting WHO guidance in 2021. When directly comparing 32 countries with policies reviewed in 2018 and 2021, [13] policy adoption increased more than 2-fold (20 vs 9). As of the 2021 review, 38% of policies were published before 2019 which underscores the need to update national testing policies at more regular intervals.

Given the rapid changes in HIV testing landscape, greater efforts are needed to support national programmes to update their national HIV testing policies at more regular intervals to keep up with latest WHO guidance. This is particularly critical now as WHO is now updating HTS guidance in 2023 and it will be essential for WHO to continue policy tracking, [10] to allocate sufficient resources, to conduct implementation science research to understand bottlenecks hinder policy change and to support country-led technical working groups to drive policy change [11—12].

For peer review only - http://bmjopen.bmj.com/site/about/guidelines.xhtml

The recommendations with highest adoption were the discontinuation of a tiebreaker (79%) and western blotting (74%) as well as the use of serial testing (62%). One likely explanation for the higher policy adoption is that these WHO´s recommendations were first published between 2012 and 2015, with the exception of the discontinuation of western blotting which was recommended in 2019. Thus, national HIV programmes have had more time to fully incorporate these recommendations into their national guidelines. Although moving away from western blotting was recommended more recently, [14] resource-limited countries in the WHO African region have been at the forefront of implementing HIV RDTs compared to other WHO regions as a way to rapidly expand and increase access to HIV testing services.[15]

Having said that, our review found that a dozen of countries, mostly in Western Africa, still use western blotting as part of the HIV testing algorithms, hindering same-day diagnoses and rapid access to ART and pre-exposure prophylaxis (PrEP). Additionally, between 2018 and 2021, countries in southern Africa appeared to be the one sub-region which shifted away from serial testing to parallel testing strategies. This shift has likely increased testing costs as it requires more test kits per individual tested. Further follow-up with countries is needed to understand their policies and should continue to promote serial testing.

Despite observing that many countries recommended retesting prior to ART initiation in 2021, we noted that progress in policy adoption was minimal and that in southern Africa one less country was implementing when compared to 2018. And only half of countries (49%) had adopted retesting prior to ART initiation in their policies. Retesting prior to ART initiation remains an important quality assurance strategy, however if countries are struggling to adopt

For peer review only - http://bmjopen.bmj.com/site/about/guidelines.xhtml

this recommendation it may be important for WHO to provide alternative quality assurance approaches and address implementation challenges.

The WHO´s recommendations with the lowest uptake were the use of a standard 3-test strategy (45%) and the use of dual HIV/syphilis RDT among pregnant women (45%), both recommendations released in 2019.[6] Before 2019, WHO recommended the use of a 2-test strategy in high HIV prevalence settings (≥5%) and the use of a 3-test strategy in low HIV prevalence settings (<5%) to maintain at least a 99% positive predictive value.[1] However, as HTS and ART have been scaled up substantially and fewer people undergoing HTS are HIV-positive, the national HTS positivity has also declined, even in high HIV burden settings.[16] Given these changes in the epidemic, WHO now recommends that all settings move toward using a 3-test strategy to ensure high-quality testing.[16]

Transition to a 3-test strategy should be a priority, particularly for the high HIV burden countries that were using a 2-test strategy at the time of this review. This review found that only 9% (n=1/11) of the high-burden countries in the WHO African region has a supportive policy on a 3-assay testing strategy. A likely explanation for this slower policy adoption is programmes needing to prioritise limited resources and efforts to address the COVID-19 pandemic. Many programmes, including those focused on HIV testing, were affected by disruptions during COVID-19 and updates and changes to national policies were delayed.[4] Changing a testing algorithm also takes time and planning to implement, including provider training, quality assurance, procurement, inventory management and budget implications to the national programme.[6,16] Many countries indicated they were in the process of transitioning to a 3-test strategy but needed to finalize guidance, complete verification

For peer review only - http://bmjopen.bmj.com/site/about/guidelines.xhtml

studies, conduct tendering process for products, and garner consensus and support among partners. For example, after the review was completed, Malawi updated their guidance and currently recommends a 3-test strategy. However, some countries have yet to prioritise adopting the 3-test strategy because they were focused on higher yield testing approaches alone, [17—18] or delayed the transition due to costs concerns. While countries will need to develop a plan and identify the optimal time for the transition, in terms of costs, a modelling study found that the total cost of the 3-test strategy would have a very limited impact on costs.[19] Further, in Ghana after transitioning to a 3-test strategy they found that while there were lessons learned about logistics and training, the new strategy was found to be feasible, improved testing quality and reduced costs due to preventing misdiagnosis.[20]

We found that among low-prevalence countries (n=34), a significant proportion (41%) still use a suboptimal testing strategy with 2 assays to make an HIV positive diagnosis, which will increase the likelihood of false-positive diagnosis.[19—21] A retrospective study in Nigeria using household survey data found that the performance of the 2-test strategy in a low prevalence setting of about 1.4% was poor with a PPV of 94% and a false-positive rate of 5.5%.[22] This positive predictive value is similar to a modelling study assessing the accuracy of the 2015 and 2019 WHO HIV testing algorithms, which was 95.4% using a 2-assay testing strategy.[19]

Considering that the recommendation to use dual HIV/syphilis RDTs in ANC was released only in 2019,[6, 23] its adoption by 21 countries has been encouraging. The number of countries adopting dual HIV/syphilis RDTs among pregnant women continues to increase and may be in practice higher considering that preliminary National Commitments and Policy Instrument

For peer review only - http://bmjopen.bmj.com/site/about/guidelines.xhtml

(NCPI) survey data reported that a total of 26 countries in the WHO African region adopted the use of dual HIV/syphilis RDTs in ANC. [24] The differences likely reflect district methodologies (policy reviews versus policy survey). Often annual global policy surveys, like NCPI, may reflect a mixture of policies and anecdotal practices, including unofficial policies not yet within published guidelines. Although the findings of this review likely provide a conservative estimate of policy adoption, they do highlight the need to ensure official national policies are updated accordingly.

Some countries may choose not to introduce dual HIV/syphilis RDT for pregnant women. For example, in Malawi where dual HIV/syphilis RDT introduction has been considered but not adopted, the national programme has opted to retain stand-alone HIV and syphilis RDTs in ANC to prioritize point-of-care test options for HIV and syphilis while maintaining a single HIV testing algorithm in a resource limited context. At the time of this review, three countries also had policies using the dual test outside of pregnant women, including key populations and male partners of pregnant women. As of 2023, WHO now recommends dual HIV/syphilis testing for key populations and those with high ongoing HIV risk and this may further increase its use in HIV testing algorithms in national programmes. [25] It is an important option to increase HIV and syphilis detection and treatment and a recent modelling study in Viet Nam shows that it was cost saving or cost-effective.[26]

As countries update their national HIV testing guidelines to incorporate WHO guidance and adapt their national HIV testing strategies and algorithms to shift toward using three consecutive reactive serology tests to diagnose HIV, introduce dual HIV/syphilis RDTs among pregnant women and key populations, move away from western blotting, reinforcing

retesting prior to ART initiation as well as design flexible algorithms to address kit shortages, WHO encourages national programmes to conduct a verification study to provide objective evidence, before national scale-up, that a specific combination of products work well together without sharing false-reactivity, thus reducing the risk of misdiagnosis.[6, 9] To assist countries, WHO has put together a practical toolkit to accelerate policy adoption.[27] To date, the following African countries worked directly with WHO to update their testing algorithm: Burkina Faso, Cameroon, Chad, Central African Republic, Cote D´Ivoire, Democratic Republic of Congo, eSwatini, Kenya, Lesotho, Malawi, Mali, South Sudan and Zambia.

This policy review has strengths as well as weaknesses. Firstly, this review provides a comprehensive review of existing national HTS policies in the African region and was able to identify current policies for 45 countries. While this represents nearly all countries in the region, we were unable to identify policies for two countries. Secondly, unlike other policy surveys this study reviewed official policies for each country that were collected through a robust global search and triangulated data with additional information provided by country-level key informants. Despite this, the review was unable to assess unpublished policies and those in development at the time of the analysis. During the review some countries indicated that they were in the process of updating their guidance, particularly around adoption of a 3-test strategy. We were unable to fully include this information in the review, and thus our reported level of adoption of WHO recommendations may be an underestimate. Thirdly, this review did not assess policy implementation at the national, subnational, or site level. Thus, it is possible that some practices in country differ than policies reviewed, and level of adoption reported may vary. Lastly, we focused on HIV testing strategies for individuals ≥18 months of

age. As a result, we were unable to report on the adoption of HIV testing strategy recommendations for children, such as early infant diagnosis.

Conclusions

Adoption of WHO-recommended HIV testing strategies has improved in the African region. While WB was only used in a few countries, concerted efforts are still needed to phase out this technology in favour of RDTs. Countries should plan to accelerate their transition to WHO recommendations by streamlining efforts to adopt and implement a 3-asssay testing strategy, retesting before ART initiation and use of dual HIV/syphilis RDTs. Conducting verification of testing algorithms and using appropriate assays can ensure accurate HIV diagnosis in a cost-efficient and time-efficient manner. Greater efforts are needed now to ensure countries implement high-quality testing services as they are essential for the global goal to achieve and maintain low HIV incidence.

Acknowledgements

We would like to express our gratitude to WHO colleagues in the respective countries of the WHO African region for their support in locating updated national policy documents. We would also like to thank staff at the Global Fund (Youssouf Sawadogo, Ghislaine Grasser and Jacqueline Papo), CHAI (Katherine Guerra, Gillian Leitch, Megan Ginivan and Christian Stillson), Evidence Action Group (Anna Konstantinova and Emilie Efroson) and staff from Ministry of Health in Mozambique (Helga Guambe) and Guinea Bissau (David da Silva Té) for their support in identifying latest national HIV testing policies.

For peer review only - http://bmjopen.bmj.com/site/about/guidelines.xhtml

Contributors

CJ and AS devised and CJ and RB supervised the review. EF led the study protocol developments, conducted the screening, data extraction and analysis. NB, CM, NSB, FSM, FJL, MASP, LH assisted with acquisition of policies, data collection and results interpretation. CL acted as a second reviewer and CJ as third reviewer. AS, MSJ, MBD and RB assisted with assessing policies and interpreting results. EF led manuscript writing along with CJ, with support from all authors. CL, NB, CM, NSB, FSM, FJL, MASP, LH, MBD, MSJ, AS, RB all reviewed the first draft, provided critical review and input and approved the final version of the manuscript.

Funding statement

This work was supported by the Bill and Melinda Gates Foundation, grant number INV-024432. The funders had no role in study design, data collection and analysis, decision to publish, or preparation of the manuscript. Authors declare no competing interests. The views expressed in this manuscript are those of the authors and do not necessarily represent the official position of the WHO.

Map disclaimer

The depiction of boundaries on the map(s) in this article do not imply the expression of any opinion whatsoever on the part of BMJ (or any member of its group) concerning the legal status of any country, territory, jurisdiction or area or of its authorities. The map(s) are provided without any warranty of any kind, either express or implied.

Competing interests

The authors declare no competing interests. The contents in this article are those of the authors and do not necessarily reflect the view of the World Health Organization.

Patient consent for publication

Not required.

Ethics approval

This study does not involve human participants and ethical approval was therefore not required.

Data availability statement

All data generated in the study are included in the article or uploaded as supplementary information. National policies may be publicly available. Some policies included in this review may be available through the following websites: (1) https://aidsfree.usaid.gov/resources/guidance-data/hts. (2) http://www.hivpolicywatch.org/database.html. If information on a policy cannot be found through these resources, please contact the authors of this review for additional information.

REFERENCES

1. Consolidated guidelines on HIV testing services, 2015. Geneva: World Health Organization; 2015. Available from: https://www.who.int/publications/i/item/9789241508926 [cited 2022 Feb 24].

2. Guidelines on when to start antiretroviral therapy and on pre-exposure prophylaxis for HIV, 2015. Geneva: World Health Organization; 2015. Available from: https://www.who.int/publications/i/item/9789241509565 [cited 2022 Feb 24].

3. Frescura L, Godfrey-Faussett P, Feizzadeh A A, El-Sadr W, Syarif O, Ghys PD; on and behalf of the 2025 testing treatment target Working Group. Achieving the 95 95 95 targets for all: A pathway to ending AIDS. PLoS One. 2022 Aug 4;17(8):e0272405.

4. Assessment of HIV testing services and antiretroviral therapy service disruptions in the context of COVID-19: lessons learned and way forward in sub-Saharan Africa. World Health Organization; 2021. Available from: https://www.who.int/publications/i/item/9789240039599 [cited 2022 Feb 24].

5. Fact sheet. Latest global and regional statistics on the status of the AIDS epidemic [internet]. Geneva: United Nations Program on HIV/AIDS (UNAIDS); 2021. Available from: https://www.unaids.org/sites/default/files/media_asset/UNAIDS_FactSheet_en.pdf [cited 2022 Feb 24].

6. Consolidated guidelines on HIV testing services, 2019. Geneva: World Health Organization; 2019. Available from: https://www.who.int/publications/i/item/978-92-4-155058-1 [cited 2022 Feb 24].

7. Service delivery approaches to HIV testing and counselling (HTC): a strategic HTC programme framework, 2012. Geneva: World Health Organization; 2012. Available from: https://apps.who.int/iris/handle/10665/75206 [cited 2022 Oct 10].

8. Tippett Barr BA, Lowrance D, Johnson CC, Baggaley RC, Rogers JH, Balachandra SK, Barker J, Kalua T, Bunga S, Low-Beer D, Payne D, Bulterys MG, Jahn A. Treatment-adjusted prevalence to assess HIV testing programmes. Bull World Health Organ. 2021 Dec 1;99(12):874-882.

9. Optimizing HIV testing algorithms: a generic verification protocol for selecting appropriate HIV serology assays and assessing the level of shared false-reactivity. Geneva: World Health Organization; 2021. Available from: https://www.who.int/publications/i/item/9789240039162 [cited 2022 Feb 24].

10. Flodgren G, Hall AM, Goulding L, Eccles MP, Grimshaw JM, Leng GC, Shepperd S. Tools developed and disseminated by guideline producers to promote the uptake of their guidelines. Cochrane Database Syst Rev. 2016 Aug 22;(8):CD010669. doi: 10.1002/14651858.CD010669.pub2.

11. Nilsen P, Ståhl C, Roback K, Cairney P. Never the twain shall meet?--a comparison of implementation science and policy implementation research. Implement Sci. 2013 Jun 10;8:63. doi: 10.1186/1748-5908-8-63..

12. Oh A, Abazeed A, Chambers DA. Policy Implementation Science to Advance Population Health: The Potential for Learning Health Policy Systems. Front Public Health. 2021 Jun 17;9:681602. doi: 10.3389/fpubh.2021.681602.

13. Fonner VA, Sands A, Figueroa C, Baggaley R, Quinn C, Jamil MS, Johnson C. Country adherence to WHO recommendations to improve the quality of HIV diagnosis: a global policy review. BMJ Glob Health. 2020 May;5(5):e001939.

14. WHO recommends countries move away from the use of western blotting and line immunoassays in HIV testing strategies and algorithms. Geneva: World Health Organization; 2019. Available from: https://www.who.int/publications/i/item/WHO-CDS-HIV-19.30 [cited 2022 Oct 10].

15. Flynn DE, Johnson C, Sands A, Wong V, Figueroa C, Baggaley R. Can trained lay providers perform HIV testing services? A review of national HIV testing policies. BMC Res Notes. 2017 Jan 4;10(1):20. doi: 10.1186/s13104-016-2339-1. PMID: 28057054; PMCID: PMC5216526.

16. WHO encourages countries to adapt HIV testing strategies in response to changing epidemic. Geneva: World Health Organization; 2019. Available from: https://www.who.int/publications/i/item/WHO-CDS-HIV-19.34 [cited 2022 Oct 10].

17. Jubilee M, Park FJ, Chipango K, Pule K, Machinda A, Taruberekera N. HIV index testing to improve HIV positivity rate and linkage to care and treatment of sexual partners, adolescents and children of PLHIV in Lesotho. PLoS One. 2019 Mar 27;14(3):e0212762.

18. Ahmed S, Schwarz M, Flick RJ, Rees CA, Harawa M, Simon K, Robison JA, Kazembe PN, Kim MH. Lost opportunities to identify and treat HIV-positive patients: results from a baseline assessment of provider-initiated HIV testing and counselling (PITC) in Malawi. Trop Med Int Health. 2016 Apr;21(4):479-85.

19. Eaton JW, Sands A, Barr-DiChiara M, Jamil MSJ, Baggaley R, Tippett Barr BA, Kalua T, Jahn A, Maheu-Giroux, Johnson. Accuracy and performance of the WHO 2015 and WHO 2019 HIV testing strategies across epidemic settings. medRxiv 2021.03.31.21254700. Available from: https://www.medrxiv.org/content/10.1101/2021.03.31.21254700v1. [cited 2022 Oct 10].

20. Rowland Adukpo. Experience from Ghana in transitioning to a 3-assay HIV testing strategy. WHO satellite session "Improving quality of HIV diagnosis: do you know how to update your HIV national testing algorithms? At the African Society of Laboratory Medicine Conference, 18 Nov 2021. Available from: https://www.who.int/news-room/events/detail/2021/11/18/default-calendar/improving-quality-of-hiv-diagnosis-toolkit-launch [cited 2022 Oct 10].

21. Johnson CC, Fonner V, Sands A, Ford N, Obermeyer CM, Tsui S, Wong V, Baggaley R. To err is human, to correct is public health: a systematic review examining poor quality testing and misdiagnosis of HIV status. J Int AIDS Soc. 2017 Aug 29;20(Suppl 6):21755.

22. Patel HK, Ikpe S, Bronson M, Birhanu S, Abimiku A, Jahun I, Detorio M, Lupoli K, Yavo D, Bassey OO, Jelpe TD, Kagurusi B, Iriemenam NC, et al. (2022) Performance of HIV rapid testing algorithm in Nigeria: Findings from a household-based Nigeria HIV/AIDS Indicator and Impact Survey (NAIIS). PLOS Glob Public Health 2022 2(7): e0000466.

23. Dual HIV/syphilis rapid diagnostic tests can be used as the first test in antenatal care. Geneva: World Health Organization; 2019. Available from: https://www.who.int/publications/i/item/WHO-CDS-HIV-19.38. [cited 2022 Oct 10].

24. UNAIDS Joint United Nations Programme on HIV/AIDS and WHO. Laws and policies analytics. Available from: https://lawsandpolicies.unaids.org/topicresult?i=13&o=219,397&lan=en. [cited 2022 Oct 10].

25. Consolidated guidelines on HIV, viral hepatitis and STI prevention, diagnosis, treatment and care for key populations. Geneva: World Health Organization; 2022. Available from: https://www.who.int/publications/i/item/9789240052390. [cited 2022 Oct 10].

26. Coomes D, Green D, Barnabas R, Sharma M, Barr-DiChiara M, Jamil MS, Baggaley R, Owiredu MN, Macdonald V, Nguyen VTT, Vo SH, Taylor M, Wi T, Johnson C, Drake AL. Cost-effectiveness of implementing HIV and HIV/syphilis dual testing among key populations in Viet Nam: a modelling analysis. BMJ Open. 2022 Aug 11;12(8):e056887.

27. Toolkit to optimize HIV testing algorithms. Geneva: World Health Organization; 2021. Available from: https://www.who.int/tools/optimizing-hiv-testing-algorithms-toolkit. [cited 2022 Oct 11].

For peer review only - http://bmjopen.bmj.com/site/about/guidelines.xhtml

Figure legends

Figure 1. Countries from the WHO African Region with HIV testing policies identified (in blue) and included in the analysis (n=45/47). The two countries not included in the review are indicated in the map.

Figure 2. Overall adoption of 2019 WHO HIV testing strategies in Africa by subregion in 2021.

Figure 3. Policy adoption in 2021 of six specific WHO HIV testing recommendations in the WHO African Region.

For peer review only - http://bmjopen.bmj.com/site/about/guidelines.xhtml

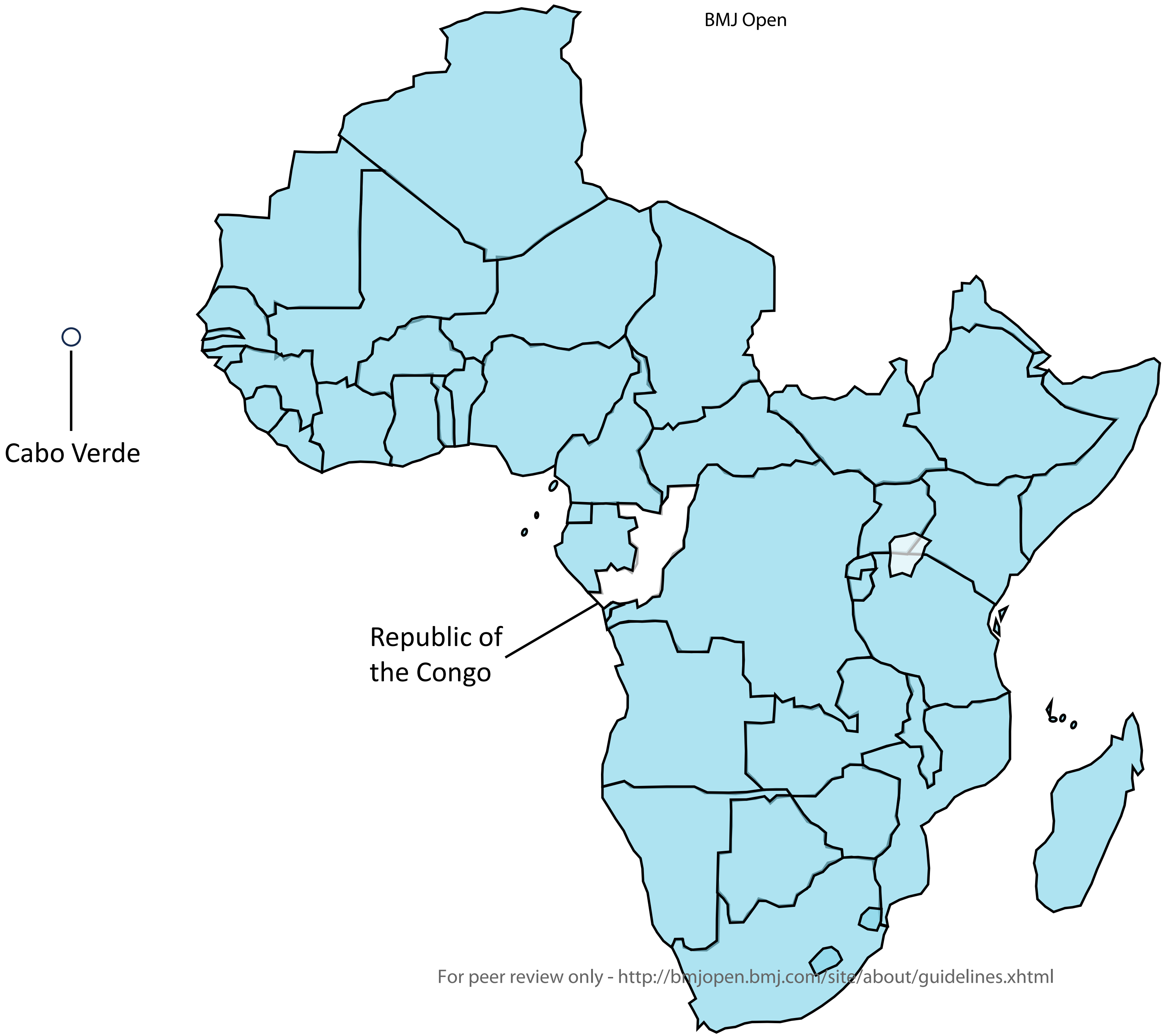
Cabo Verde
Republic of the Congo

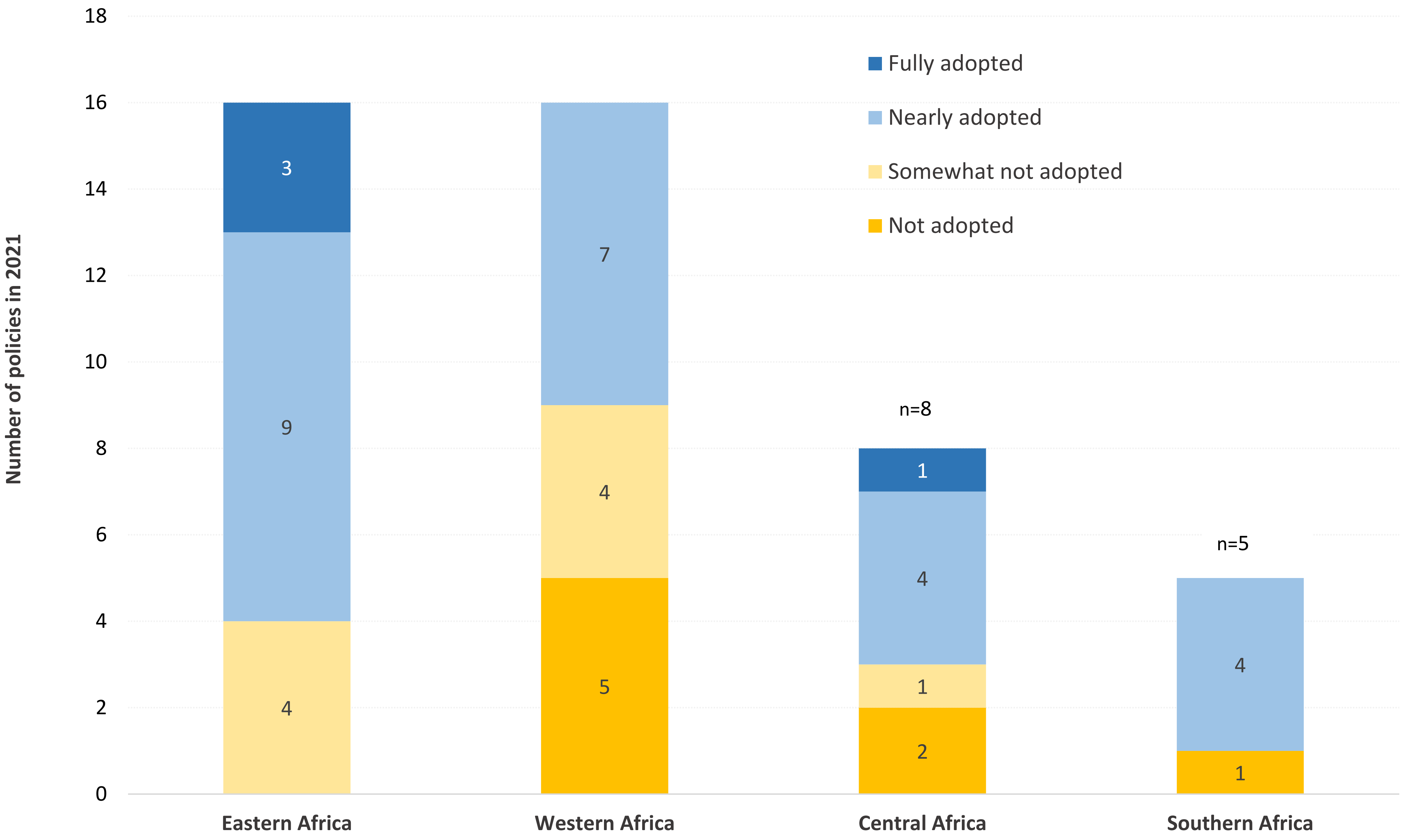
Number of policies in 2021
18
16
14
12
10
8
6
4
2
0
Fully adopted
Nearly adopted
Somewhat not adopted
Not adopted
3
9
4
7
4
5
n=8
1
4
1
2
n=5
4
1
Eastern Africa
Western Africa
Central Africa
Southern Africa

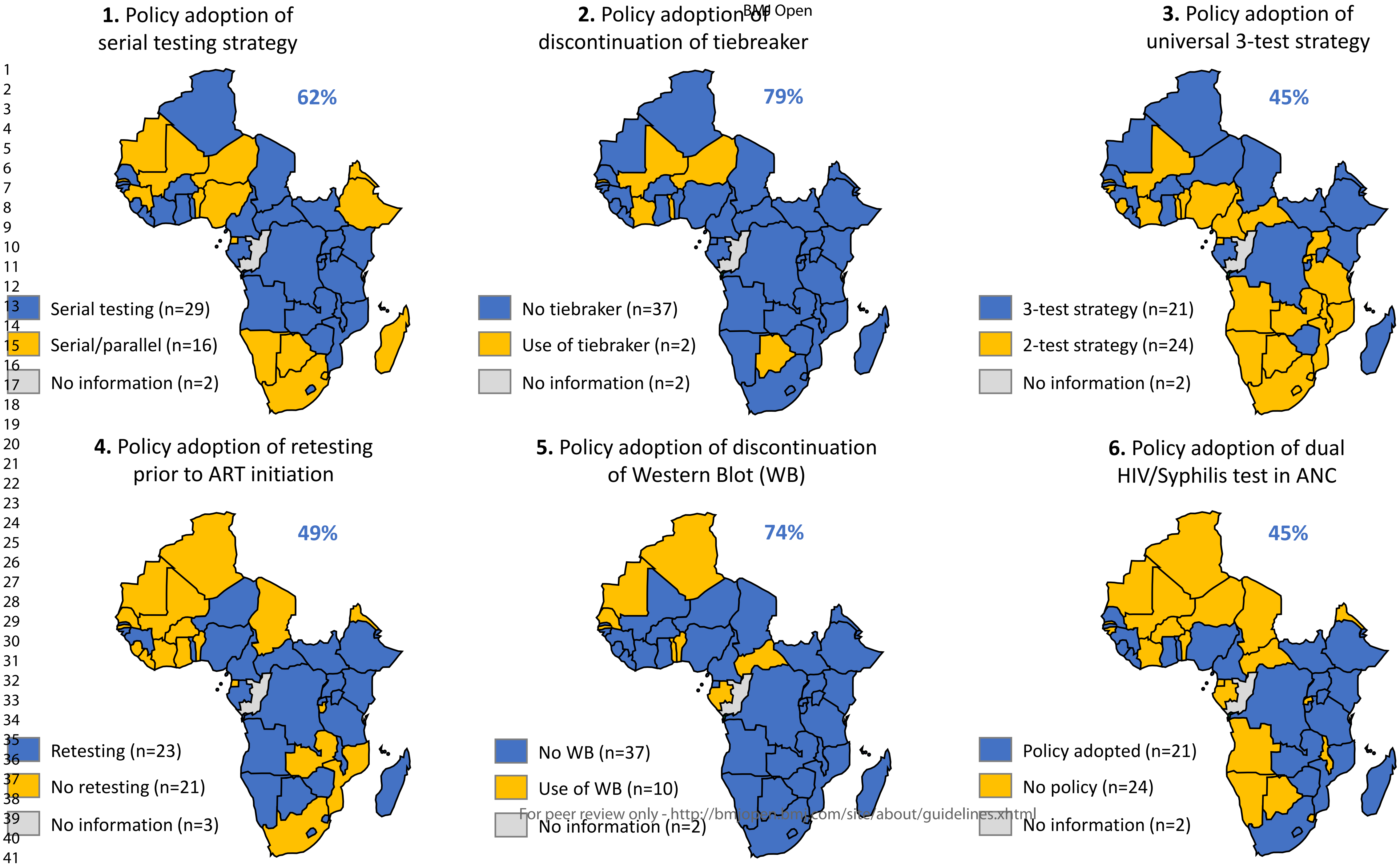
1. Policy adoption of serial testing strategy
62%
Serial testing (n=29)
Serial/parallel (n=16)
No information (n=2)
2. Policy adoption of discontinuation of tiebreaker
79%
No tiebraker (n=37)
Use of tiebraker (n=2)
No information (n=2)
3. Policy adoption of universal 3-test strategy
45%
3-test strategy (n=21)
2-test strategy (n=24)
No information (n=2)
4. Policy adoption of retesting prior to ART initiation
49%
Retesting (n=23)
No retesting (n=21)
No information (n=3)
5. Policy adoption of discontinuation of Western Blot (WB)
74%
No WB (n=37)
Use of WB (n=10)
No information (n=2)
6. Policy adoption of dual HIV/Syphilis test in ANC
45%
Policy adopted (n=21)
No policy (n=24)
No information (n=2)

Supplementary Appendix

Country adoption of WHO 2019 guidance on HIV testing strategies and algorithms: a policy review across the WHO African region

Emmanuel Fajardo,[1] Céline Lastrucci,[1] Nayé Bah,[2] Casimir Manzengo Mingiedi,[3] Ndoungou Salla Ba,[4] Fausta Shakiwa Mosha,[5] Frank John Lule,[6] Margaret Alia Sampson Paul,[6] Lago Hughes,[6] Magdalena Barr-DiChiara,[1] Muhammad Shahid Jamil,[1] Anita Sands,[7] Rachel Baggeley,[1] Cheryl Johnson[1]

Supplementary Figure 1. Countries in the WHO African Region and grouping by subregion

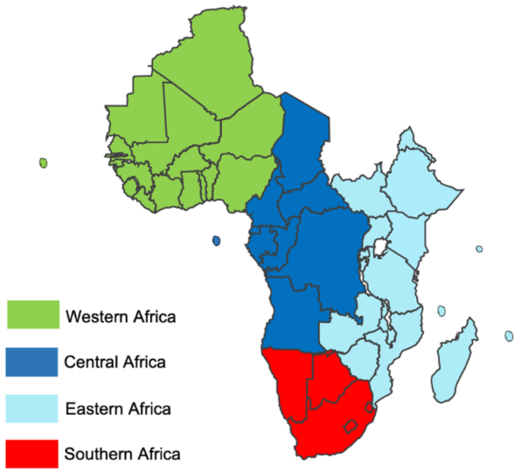

WHO African Region (n=47)			
Western Africa (n=17)	Central Africa (n=9)	Eastern Africa (n=16)	Southern Africa (n=5)
Algeria	Angola	Burundi	Botswana
Cabo Verde	Cameroon	Comoros	eSwatini
Burkina Faso	CAR	Eritrea	Lesotho
Benin	Chad	Ethiopia	Namibia
Ghana	DRC	Kenya	South Africa
Guinea	Equotorial Guinea	Madagascar	
Guinea-Bissau	Gabon	Malawi	
Ivory Coast	Republic of the Congo	Mauritius	
Liberia	Sao Tome and Principe	Mozambique	
Mauritania		Rwanda	
Mali		Seychelles	
Niger		South Sudan	
Nigeria		Tanzania	
Senegal		Uganda	
Sierra Leone		Zambia	
The Gambia		Zimbabwe	
Togo			

For peer review only

Supplementary Table 1. Categories for data extraction

**1. Policy Information**	
▪ Country<br>▪ WHO region<br>▪ HIV testing policy identified?	▪ Year of policy publication<br>▪ Type of policy document<br>▪ National HIV prevalence
**2. HIV testing algorithm (after 18 months of age)**	
▪ HIV testing algorithm provided?<br>▪ Image or text format?<br>▪ Type of scenario in which algorithm is used<br>▪ Number of assays used in algorithm<br>▪ Serial or parallel strategy<br>▪ Use of tiebreaker?<br>▪ Use of Western Blot or LIA?<br>▪ Mention of test order relating to sens/spec?<br>▪ High or low prevalence strategy used?<br>▪ Type of tests mentioned?<br>▪ Brand of tests mentioned?<br>▪ Are assays pre-qualified by WHO?<br>▪ A1 test name<br>▪ A1 type of assay	▪ A1 sens-/spec<br>▪ A2 test name<br>▪ A2 type of assay<br>▪ A2 sens-/spec<br>▪ A3 test name<br>▪ A3 type of assay<br>▪ A3 sens-/spec<br>▪ Source of assay sens/spec<br>▪ Mention of in-country assay validation<br>▪ Alignment with WHO policy?<br>▪ Primary reason(s) algorithm fails to meet WHO recommendations<br>▪ Other notes about testing strategy
**3. HIV testing algorithm among pregnant women using dual HIV/Syphilis RDTs**	
▪ HIV testing algorithm provided?<br>▪ Image or text format?<br>▪ Type of scenario in which algorithm is used<br>▪ Type of scenario in which algorithm should not be used (women on ART, already diagnosed/treated for syphilis, retesting for HIV)<br>▪ What is there syphilis testing strategy for women with HIV?<br>▪ Is dual test used as A1 or A0?<br>▪ Is the dual test algorithm aligned with the national HIV testing algorithm?<br>▪ Is the dual test algorithm aligned with the national syphilis testing algorithm?<br>▪ Brand of dual test mentioned?	▪ Is dual test prequalified by WHO?<br>▪ Dual test name<br>▪ Mention of penicillin treatment for reactive TP?<br>▪ Mention of further testing for reactive TP?<br>▪ Mention of in-country assay validation<br>▪ Alignment with WHO policy?<br>▪ Primary reason(s) algorithm fails to meet WHO recommendations<br>▪ Other notes about testing strategy
**4. Retesting prior to ART initiation**	
▪ Retest required before starting ART?<br>▪ Retest strategy same as national algorithm?<br>▪ Notes about retesting strategy	

For peer review only - http://bmjopen.bmj.com/site/about/guidelines.xhtml

Supplementary Table 2. List of national policies on HIV testing services in the WHO African Region collected in 2018 and 2021 by subregion

Subregion	Count	Country	Policy reviewed in 2018?	New policy or algorithm identified?	Publication year	Type of document	Language	Algorithm provided?	National HIV prevalence
Central Africa	1	Angola	Yes, 2015	Yes	2020	HTS	Portuguese	Yes	Low
	2	Cameroon	Yes, 2015	Yes	2019	ART	French	Yes	Low
	3	CAR	Yes, 2010	Yes	2018	ART	French	Yes	Low
	4	Chad	Yes, 2011	Yes	2017	HTS	French	Yes	Low
	5	DRC	Yes, 2017	Yes	2020	ART	French	Yes	Low
	6	Equatorial Guinea	No	Yes	2018	ART	Spanish	Yes	Low
	7	Gabon	No	Yes	2017	HTS	French	Yes	Low
	8	Republic of the Congo	No	**No**	–	–	–	–	–
	9	São Tomé e Principe	No	Yes	2018	Algorithm	Portuguese	Yes	Low
Eastern Africa	10	Burundi	Yes, 2016	Yes	2020	HTS	French	Yes	Low
	11	Comoros	Yes, 2007	Yes	2016	HTS	French	Yes	Low
	12	Eritrea	No	Yes	2019	HTS	English	Yes	Low
	13	Ethiopia	Yes, 2017	Yes	2018	ART	English	Yes	Low
	14	Kenya	Yes, 2017	Yes	2021	HTS	English	Yes	Low
	15	Madagascar	Yes, 2011	Yes	2018	HTS	French	Yes	Low
	16	Malawi*	Yes, 2016	Yes*	2016	HTS	English	Yes	High
	17	Mauritius	No	Yes	2020	HTS	English	Yes	Low
	18	Mozambique	Yes, 2016	Yes	2020	PMTCT	Portuguese	Yes	High
	19	Rwanda	Yes, 2016	Yes	2018	HTS	English	Yes	Low
	20	Seychelles	No	Yes	2019	HTS	English	Yes	Low
	21	South Sudan	Yes, 2017	Yes	2020	ART	English	Yes	Low
	22	Tanzania	Yes, 2017	Yes	2021	HTS	English	Yes	High
	23	Uganda	Yes, 2016	Yes	2020	ART	English	Yes	High
	24	Zambia	Yes, 2018	Yes	2020	HTS	English	Yes	High
	25	Zimbabwe*	Yes, 2016	Yes*	2016	Other	English	Yes	High
Southern Africa	26	Botswana**	Yes, 2016	**No**	2016	ART	English	Yes	High
	27	eSwatini**	Yes, 2018	**No**	2018	ART	English	Yes	High
	28	Lesotho	Yes, 2016	Yes	2021	PMTCT	English	Yes	High

For peer review only - http://bmjopen.bmj.com/site/about/guidelines.xhtml

	29	Namibia	Yes, 2016	Yes	2018	HTS	English	Yes	High
	30	South Africa	Yes, 2016	Yes	2020	ART	English	Yes	High
Western Africa	31	Algeria**	Yes, 2013	**No**	2013	HTS	French	Yes	Low
	32	Benin	No	Yes	2017	HTS	French	Yes	Low
	33	Burkina Faso	Yes, 2008	Yes	2021	ART	French	Yes	Low
	34	Cabo Verde	No	**No**	–	–	–	–	–
	35	Ghana	Yes, 2014	Yes	2019	ART	English	Yes	Low
	36	Guinea	No	Yes	2019	HTS	French	Yes	Low
	37	Guinea-Bissau	No	Yes	2021	ART	Portuguese	Yes	Low
	38	Ivory Coast**	Yes, 2016	**No**	2016	HTS	French	Yes	Low
	39	Liberia	Yes, 2015	Yes	2020	HTS	English	Yes	Low
	40	Mauritania	No	Yes	2020	HTS	French	Yes	Low
	41	Mali	No	Yes	2017	HTS	French	Yes	Low
	42	Niger	No	Yes	2020	HTS	French	Yes	Low
	43	Nigeria	Yes, 2016	Yes	2020	ART	English	Yes	Low
	44	Senegal	Yes, 2017	Yes	2018	HTS	French	Yes	Low
	45	Sierra Leone	Yes, 2017	Yes	2020	ART	English	Yes	Low
	46	The Gambia	Yes, 2014	Yes	2019	ART	English	Yes	Low
	47	Togo	No	Yes	2019	HTS	French	Yes	Low

*These countries provided an updated HIV testing algorithm despite having an old HIV testing guideline

** These countries did not provide an update HIV testing guideline but were included in the analysis

For peer review only - http://bmjopen.bmj.com/site/about/guidelines.xhtml

Supplementary Table 3. Progress on policy adoption in the WHO African Region from 2018 to 2021

Subregion	Count	Country	Policy reviewed in 2018?	Adherence in 2018	Policy reviewed in 2021	Adherence in 2021
Central Africa	1	Angola	Yes, 2015	Not adopted	2020	Mostly adopted
	2	Cameroon	Yes, 2015	Not adopted	2019	Mostly adopted
	3	CAR	Yes, 2010	Not adopted	2018	Not adopted
	4	Chad	Yes, 2011	Not adopted	2017	Mostly adopted
	5	DRC	Yes, 2017	Adopted	2020	Adopted
	6	Equatorial Guinea	No	–	2018	Not adopted
	7	Gabon	No	–	2017	Mostly adopted
	8	Republic of the Congo	No	–	–	–
	9	São Tomé e Principe	No	–	2018	Not adopted
Eastern Africa	10	Burundi	Yes, 2016	Not adopted	2020	Mostly adopted
	11	Comoros	Yes, 2007	No information	2016	Not adopted
	12	Eritrea	No	–	2019	Not adopted
	13	Ethiopia	Yes, 2017	No information	2018	Mostly adopted
	14	Kenya	Yes, 2017	Mostly adopted	2021	Adopted
	15	Madagascar	Yes, 2011	No information	2018	Mostly adopted
	16	Malawi	Yes, 2016	Not adopted	2016	Mostly adopted
	17	Mauritius	No	–	2020	Not adopted
	18	Mozambique	Yes, 2016	Not adopted	2020	Mostly adopted
	19	Rwanda	Yes, 2016	Not adopted	2018	Mostly adopted
	20	Seychelles	No	–	2019	Not adopted
	21	South Sudan	Yes, 2017	Not adopted	2020	Adopted
	22	Tanzania	Yes, 2017	Not adopted	2021	Mostly adopted
	23	Uganda	Yes, 2016	Mostly adopted	2020	Mostly adopted
	24	Zambia	Yes, 2018	Not adopted	2020	Not adopted
	25	Zimbabwe	Yes, 2016	Adopted	2016	Adopted
Southern Africa	26	Botswana	Yes, 2016	Mostly adopted	2016	Not adopted
	27	eSwatini	Yes, 2018	Not adopted	2018	Not adopted
	28	Lesotho	Yes, 2016	Adopted	2021	Mostly adopted
	29	Namibia	Yes, 2016	No information	2018	Not adopted
	30	South Africa	Yes, 2016	Mostly adopted	2020	Not adopted
Western Africa	31	Algeria	Yes, 2013	Adopted	2013	Not adopted
	32	Benin	No	–	2017	Not adopted
	33	Burkina Faso	Yes, 2008	Not adopted	2021	Mostly adopted
	34	Cabo Verde	No	–	–	–
	35	Ghana	Yes, 2014	Not adopted	2019	Mostly adopted
	36	Guinea	No	–	2019	Mostly adopted
	37	Guinea-Bissau	No	–	2021	Not adopted
	38	Ivory Coast	Yes, 2016	Not adopted	2016	Not adopted
	39	Liberia	Yes, 2015	Not adopted	2020	Mostly adopted
	40	Mauritania	No	–	2020	Not adopted
	41	Mali	No	–	2017	Not adopted
	42	Niger	No	–	2020	Not adopted
	43	Nigeria	Yes, 2016	Not adopted	2020	Mostly adopted
	44	Senegal	Yes, 2017	Mostly adopted	2018	Not adopted
	45	Sierra Leone	Yes, 2017	Not adopted	2020	Not adopted
	46	The Gambia	Yes, 2014	Not adopted	2019	Not adopted
	47	Togo	No	–	2019	Not adherent

A total of 32 countries had policies reviewed in 2018 and 2021. Those highlighted in red are policies reviewed in 2018 that were updated (n=28). Number of policies deemed adopted in 2018 (n=7) and number deemed adopted in 2021 (n=20)

For peer review only - http://bmjopen.bmj.com/site/about/guidelines.xhtml

Supplementary Figure 2. Comparing policy adoption across countries, 2018 vs 2021

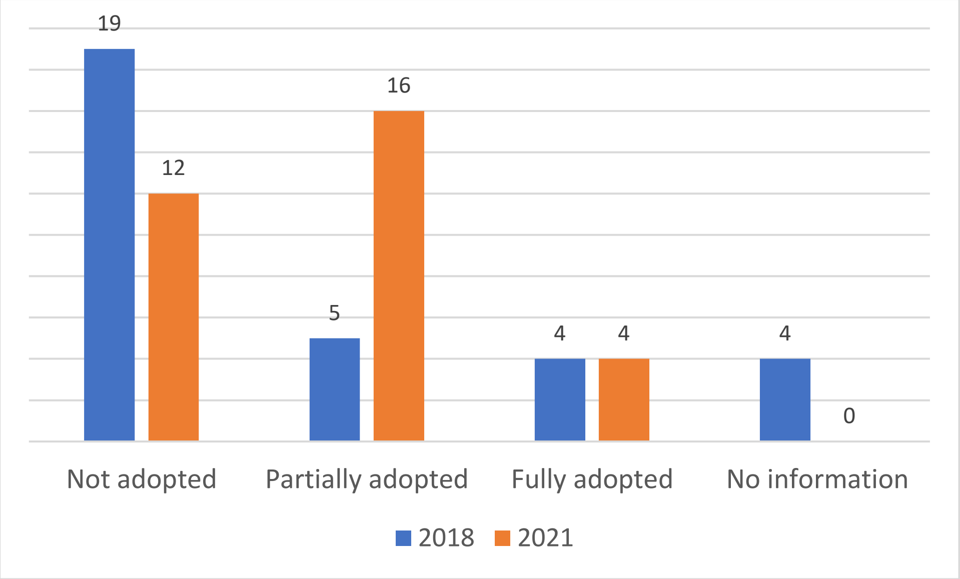

For peer review only

Supplementary Table 4. Policy adoption of 2019 WHO-recommended HIV testing strategies in the WHO African region, 2014-2021

**AFRO subregions (countries)**	**Policies reviewed**			**Serial testing strategy**			**Discontinuation of tiebreaker* testing strategy**			**Retesting prior to ART initiation**		**Universal 3-test strategy**	**Use of dual HIV/syphilis test****	**No use of Western Blot**
Year	2014	2018	2021	2014	2018	2021	2014	2018	2021	2018	2021	2021	2021	2021
All (n=47)	25 (53%)	32 (68%)	45 (96%)	21 (84%)	26 (81%)	29 (64%)	15 (60%)	26 (81%)	37 (82%)	16 (50%)	23 (51%)	21 (47%)	21 (47%)	35 (78%)
Western (n=17)	6 (35%)	9 (53%)	16 (94%)	5 (83%)	7 (78%)	9 (56%)	4 (67%)	5 (56%)	9 (56%)	1 (11%)	5 (31%)	8 (50%)	7 (44%)	10 (63%)
Central (n=9)	4 (44%)	5 (56%)	8 (89%)	4 (100%)	5 (100%)	7 (88%)	3 (75%)	4 (80%)	7 (88%)	1 (20%)	5 (63%)	3 (38%)	2 (25%)	6 (75%)
Eastern (n=16)	11 (69%)	13 (81%)	16 (100%)	10 (91%)	10 (77%)	12 (75%)	5 (45%)	12 (92%)	16 (100%)	9 (69%)	9 (56%)	9 (56%)	9 (56%)	14 (88%)
Southern (n=5)	4 (80%)	5 (100%)	5 (100%)	2 (40%)	4 (80%)	1 (20%)	3 (75%)	5 (100%)	5 (100%)	5 (100%)	4 (80%)	1 (20%)	3 (60%)	5 (100%)

*Use of a third assay to rule-in HIV infection

**Dual HIV/syphilis RDT as first test in ANC

For peer review only - http://bmjopen.bmj.com/site/about/guidelines.xhtml

Supplementary Table 5. Short name of products used as part of the 2-assay or 3-assay HIV testing algorithm in 28 HIV national testing policy documents reviewed in 2021

Strategy	Count	Country	Assay 1	Assay 2	Assay 3
2-assay	1	Angola	Determine	Uni-Gold	–
	2	Cameroon	Determine or Uni-Gold	OraQuick or Shanghai	–
	3	CAR	Determine	Uni-Gold	–
	4	Cote D'Ivoire	Determine	SD Bioline or GenieFast	StatPak or EIA<br>(as tiebreaker)
	5	Equatorial Guinea	Determine	Hexagon	Uni-Gold<br>(for discordants)
	6	eSwatini	Determine	Uni-Gold	–
	7	Gambia	Determine	SD Bioline or First Response	–
	8	Guinea Bissau	Determine	First Response	PCR<br>(as tiebreaker)
	9	Mali	Alere Combo	SD Bioline	First Response<br>(as tiebreaker)
	10	Mozambique	Determine	Uni-Gold	–
	11	Rwanda	Alere Combo	StatPak	–
	12	São Tomé e Principe	Determine	SD Bioline	PCR<br>(as tiebreaker)
	13	Sierra Leone	Determine	SD Bioline	Uni-Gold<br>(as tiebreaker)
	14	Uganda	Determine	StatPak	SD Bioline<br>(for discordants)
	15	Nigeria	Determine<br>Uni-Gold<br>Stat-Pak<br>Double Check Gold<br>Sure Check<br>HIV Quick Check	Determine<br>Uni-Gold<br>Stat-Pak<br>Double Check Gold<br>Sure Check<br>HIV Quick Check	Determine<br>Uni-Gold<br>Stat-Pak<br>Double Check Gold<br>Sure Check<br>HIV Quick Check<br>(as tiebreaker)
	16	Togo	Murex HIV Ag/Ab<br>Apdia HIV Ag/Ab<br>Determine<br>Alere Combo<br>Vikia HIV 1/2<br>Wanta Rapid Test<br>ABON HIV 1/2/0<br>Standard Q<br>Hexagon<br>Genie Fast<br>SD Bioline<br>First Response<br>SD Bioline HIV/Syphilis<br>OraQuick HIV Self-Test	ABON HIV 1/2/0<br>Standard Q<br>SD Bioline<br>First Response<br>HIV Tri-Dot	INNOLIA or Genius<br>(as tiebreaker)
3-assay	17	Burundi	Alere Combo or<br>Determine	SD Bioline or Uni-Gold	Wondfo One Step
	18	Gabon	Alere Combo	Determine	SD Bioline
	19	Ghana	First Response	OraQuick	SD Bioline
	20	Guinea	Determine	SD Bioline	Multisure
	21	Kenya	INSTI	Uni-Gold	SD Bioline
	22	Liberia	Determine	SD Bioline	Uni-Gold
	23	Madagascar	Determine	Uni-Gold	SD Bioline
	24	Mauritius	GenScreen Ultra Ag/Ab	Alere Combo	Western Blot
	25	Malawi	INSTI	Uni-Gold	SD Bioline

For peer review only - http://bmjopen.bmj.com/site/about/guidelines.xhtml

	26	Niger	Alere Combo	Wondfo One Step	SD Bioline
	27	Senegal	Determine	SD Bioline	Multisure
	28	Zimbabwe	Determine	Chembio	INSTI
	29	Burkina Faso	3rd generation:<br>Determine<br>Double check Gold Ultra<br>Onsite HIV1+2 Plus<br>Combo<br>VIKIA HIV 1/2<br>4th generation:<br>Alere Ag/Ab Combo<br>OnSite HIV Ab/Ag (CTK)	HIV TriDot<br>ImmunoFlow<br>OnSite HIV 1/2 Ab Plus (CTK)<br>SD Bioline	Any of those not selected as A1 or A2

For peer review only

For peer review only - http://bmjopen.bmj.com/site/about/guidelines.xhtml
